# Neuron-derived SPP1 instructs microglia to limit degeneration

**DOI:** 10.1126/sciadv.aee4940

**Published:** 2026-08-28

**Authors:** Song Li, Tatjana C. Jakobs

**Affiliations:** ^1^ Department of Ophthalmology, Harvard Medical School, Boston, MA 02114, USA.; ^2^ Schepens Eye Research Institute, Massachusetts Eye and Ear, Boston, MA 02114, USA.

## Abstract

Neurons actively shape immune responses that maintain central nervous system integrity. We identify SPP1 (secreted phosphoprotein 1) as a neuron-derived signal that reprograms microglia into a neuroprotective, homeostatic state after injury and during neurodegeneration. In mouse models of glaucoma and optic nerve damage, neuronal SPP1 enhances microglial autophagy, debris clearance, and anti-inflammatory activity, preserving neuronal survival and visual function. SPP1 is elevated in neurons of human and primate glaucomatous retinas, where SPP1^+^ cells show increased resilience. In Alzheimer’s disease brain, neuronal SPP1 correlates with neuronal survival, while microglia around Aβ plaques display defective autophagy. In human iPSC co-cultures, SPP1 enhances microglial Aβ clearance and prevents neurodegeneration. Thus, SPP1 defines a protective neuron–microglia axis in glaucoma and possibly other neurodegenerative diseases.

## INTRODUCTION

Communication between neurons and microglia is one of the factors determining whether immune activation protects or harms the CNS (*1*). Microglia respond to cues released by neurons, such as FGF-2 or fractalkine, which have been described as “help me” signals (*2*–*4*), or mediators like IL34 that upregulate phagocytosis and debris clearance (*5*).

The retina provides an approachable model for investigating neuron–glia communication in the context of injury (*6*). Among retinal neurons, retinal ganglion cells (RGCs) are projection neurons analogous to those in the brain (*7*). Glaucoma, a leading cause of irreversible blindness, is characterized by progressive RGC loss and serves as a model of CNS neurodegeneration (*8*). RGCs have ∼40 subtypes defined by distinct morphological, transcriptional, and electrophysiological characteristics (*9*–*11*). RGC subtypes differ in susceptibility to injury (*12*, *13*), with the large αRGCs being notably resilient (*14*). Alpha cells stain for the high-molecular-weight neurofilament subunit and are the only retinal neurons that constitutively express secreted phosphoprotein 1 (SPP1, also known as osteopontin) (*15*, *16*). SPP1 expression correlates with neuronal resilience and axonal regeneration after injury (*17*, *18*), yet its function in neurons remains unresolved. SPP1^+^ neurons are also found in the spinal cord and multiple brain regions such as the sensorimotor cortex and the developing or injured cerebellum (*19*–*22*), suggesting a conserved role across the CNS.

We recently demonstrated that astrocyte-derived SPP1 protects RGCs after optic nerve injury: conditional deletion of Spp1 in astrocytes accelerates degeneration, whereas overexpression preserves RGCs (*23*, *24*). However, this still left the question of what the function of the SPP1 expressed in αRGCs and other CNS neurons may be.

Here, we propose that neuronal SPP1 is a part of an intrinsic neuroimmune defense mechanism. This hypothesis stems from two observations: αRGC-specific deletion of Spp1 results in increased vulnerability of all retinal ganglion cells (not just the intrinsically Spp1^+^ ones) to injury, and microglia show heightened activation in Spp1 cKO retinas. In this view, αRGCs act as guardians against injury to the whole ganglion cell population. Insult to αRGC axons induces the secretion of SPP1 from αRGCs. SPP1 acts on microglial ItgαV receptors to instruct microglia to assume a more neuroprotective phenotype. The production of neuroprotective factors by microglia benefits both αRGCs and non-αRGCs, thereby closing the defensive loop. We present evidence for each step: SPP1 is upregulated in αRGCs after injury, and its conditional deletion not only affects the αRGCs themselves but also the intrinsically SPP1-negative non-αRGCs. Furthermore, SPP1 suppresses the morphological and transcriptional activation of microglia, as well as neuroinflammation, and stimulates the expression of neuroprotective factors, such as interleukin-10. This neuroprotective phenotype depends on enhancing microglial autophagy and debris clearance. These mechanisms are translated into human iPSC-derived neuron–microglia co-cultures and are evident in brain sections from patients with Alzheimer’s disease and in glaucomatous retinas from humans and nonhuman primates. Together, our findings reveal that neurons actively direct microglial protective states through SPP1 signaling, defining a conserved neuroimmune axis that preserves neuronal integrity across species and neurodegenerative contexts.

## RESULTS

### SPP1 is upregulated in αRGCs after injury

**Fig. 1.**
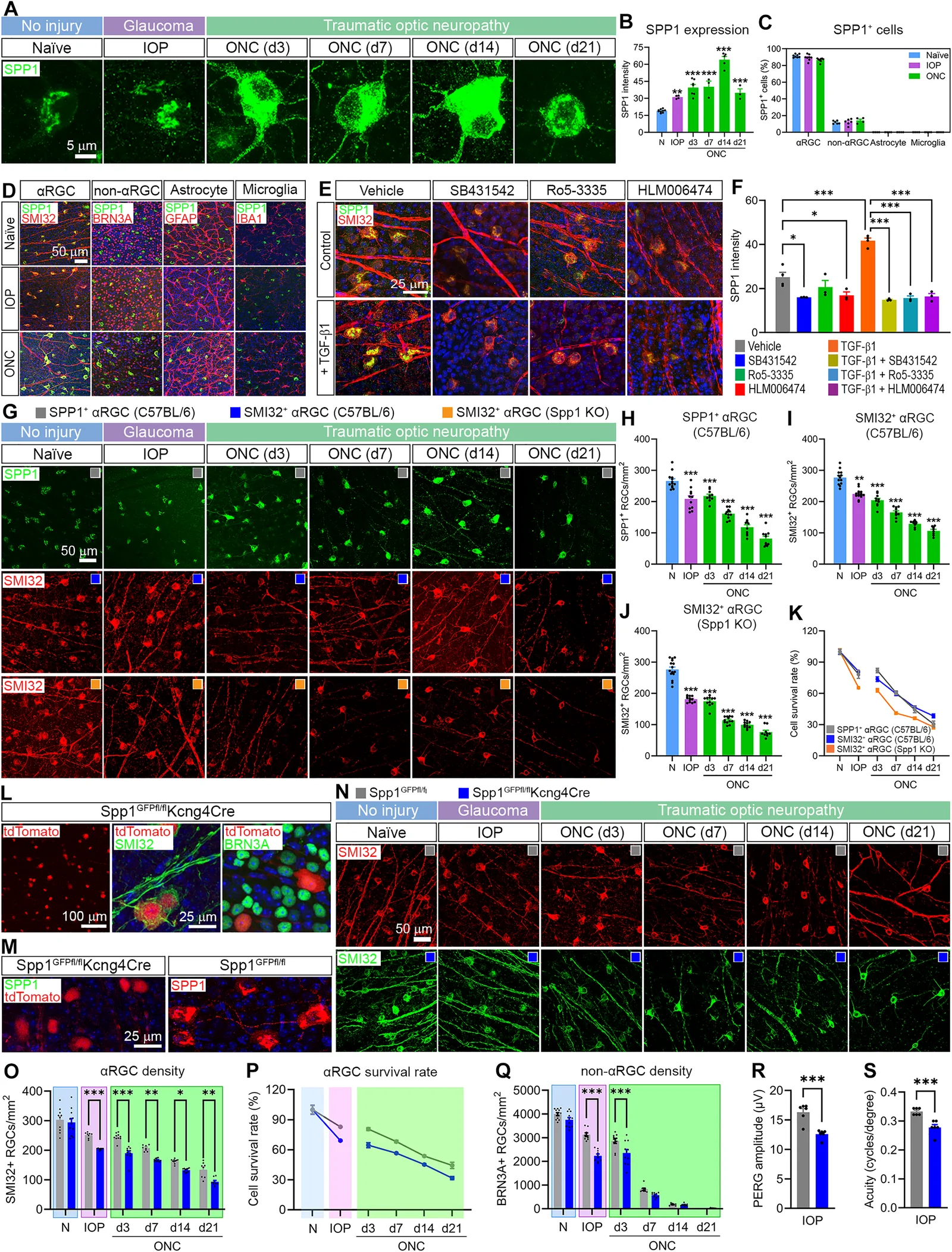
αRGC-derived SPP1 protects against neurodegeneration in glaucoma and optic nerve injury. (**A** and **B**) SPP1 expression (A) in C57BL/6 retinas under naïve conditions, 4 weeks after intraocular pressure (IOP) elevation, and at days 3–21 after optic nerve crush (ONC), with quantification (B) (*n* = 3–9 mice). (**C** and **D**) Cell-type distribution of SPP1 with SPP1^+^ cells percentages among SMI32^+^ αRGCs, BRN3A^+^ non-αRGCs, GFAP^+^ astrocytes, and IBA1^+^ microglia (C), and co-staining images (D) (*n* = 4–8 mice). (**E** and **F**) Regulation of SPP1 by TGF-β signaling and transcriptional modulators, with representative images (E) and quantification (F) following treatment with TGF-β1, TGF-β1 receptor inhibitor SB431542, RUNX1 antagonist Ro5–3335, E2F1 antagonist HLM006474, or combinations (*n* = 3–4 mice). (**G** to **K**) SPP1 and SMI32 labeling in C57BL/6 (WT) and global Spp1 knockout (Spp1 KO) mice across naïve, IOP, and ONC conditions (G), with quantification of SPP1^+^ αRGCs (H) and SMI32^+^ αRGCs (I) in WT mice, SMI32^+^ αRGCs in Spp1 KO mice (J), and survival rates (K) (*n* = 8–15 mice). (**L** and **M**) Validation of conditional Spp1 knockout mice (Spp1 cKO; Spp1^GFPfl/fl^Kcng4Cre), showing tdTomato co-localization with SMI32 but not BRN3A (L) and confirmation of Spp1 deletion (M) (*n* = 3–4 mice). (**N** to **Q**) αRGC and non-αRGC analysis in Spp1 cKO and control mice under naïve, IOP, and ONC conditions, including representative images (N) and quantification of αRGC density (O), survival rate (P), and non-αRGC density (Q) (*n* = 8–12 mice). (**R** and **S**) Pattern electroretinography (PERG) amplitude (R) and visual acuity (S) at 4 weeks after IOP elevation (n = 6 mice). Statistical analyses: one-way ANOVA [(B), (F), and (H) to (J), two-way ANOVA [(O) and (Q)], and unpaired two-tailed t-test [(R) and (S)]. *P < 0.05, **P < 0.01, ***P < 0.001. Data are mean ± SEM.

SPP1 is constitutively expressed in αRGCs, with the immunoreactivity restricted to the perinuclear region under baseline conditions. After an elevation of intraocular pressure (IOP) or optic nerve crush (ONC), SPP1 expression was significantly upregulated and distributed throughout the soma, axon, and proximal dendrites (Fig. 1, A and B). In addition to SPP1, αRGCs also stain positive for neurofilament heavy chain (SMI32), the voltage-gated potassium channel Kcng4 (fig. S1, A and B), and weakly for the general ganglion cell marker Brn3a (*17*, *25*–*27*). By this staining pattern, SMI32^high^/Brn3a^low^ cells accounted for >90% of SPP1^+^ positive cells in the retina (Fig. 1, C and D). Optic nerve injury did not induce SPP1 expression in non-alpha cells or other retinal cells such as Müller cells, astrocytes, and microglia (Fig. 1, C and D and fig. S1, C to F). This contrasted with the optic nerve, where injury induces robust upregulation of SPP1 in astrocytes (*23*).

Expression of TGFβ1 is increased in glaucoma (*28*), and TGFβ1 was described as a positive regulator of SPP1 expression in other cell types (*23*, *29*). We tested whether this is also the case for αRGCs by intravitreal injection of TGFβ1 with and without the TGFβ1 receptor inhibitor SB431542. The TGFβ1-induced upregulation of SPP1 in αRGCs was completely blocked by the receptor inhibitor and by inhibition of the downstream transcription factors Runx1 and E2F1 using Ro5–3335 and HLM006474, respectively (Fig. 1, E and F).

### Conditional deletion of Spp1 in αRGCs exacerbates RGC loss in glaucoma and traumatic nerve damage

After glaucomatous or traumatic damage to the optic nerve, αRGCs degenerate and die (Fig. 1, G to I). In a global Spp1 knockout (Spp1 KO), the αRGC loss was more pronounced than that in wild-type mice (Fig. 1, J and K). This does not yet prove that it is specifically αRGC-derived SPP1 that is causative, as SPP1 can also be secreted by optic nerve astrocytes (*23*). We therefore created an αRGC-specific deletion of Spp1 strain (Spp1^GFPfl/fl^Kcng4Cre, Spp1 cKO) by crossing Spp1^GFPfl/fl^ mice with B6.Kcng4-cre mice (fig. S2A). In this strain, deletion of the Spp1 gene also activates the transcription of a tdTomato reporter gene, so that αRGCs after recombination can be identified by their red fluorescence in addition to the high expression of neurofilament H (Fig. 1, L and M and fig. S2B). Elevated IOP and traumatic optic nerve damage in αRGC-specific Spp1 cKO mice lead to increased RGC loss (Fig. 1, N to Q). Of note, the increased cell loss was not limited to the αRGCs but was observed for Brn3a^high^ RGCs as well (Fig. 1Q and fig. S2C). Increased IOP caused an expected reduction in pattern electroretinogram (pERG) amplitude and visual acuity, reflecting a functional loss of RGC activity. In the Spp1 cKO strain, pERG and visual acuity were more severely affected than in the wild-type controls (Fig. 1, R and S).

The observation that Spp1 deletion from the endogenously SPP1^+^ αRGCs makes these cells more susceptible to damage is consistent with a cell-autonomous protective effect of SPP1 on the cells that express it (*18*). However, SPP1 was also protective of other ganglion cell types, suggesting that SPP1 secreted from αRGCs has a neuroprotective effect on neighboring ganglion cells, either directly or mediated by another cell type.

### SPP1 deficiency exacerbates microglia activation after injury

**Fig. 2.**
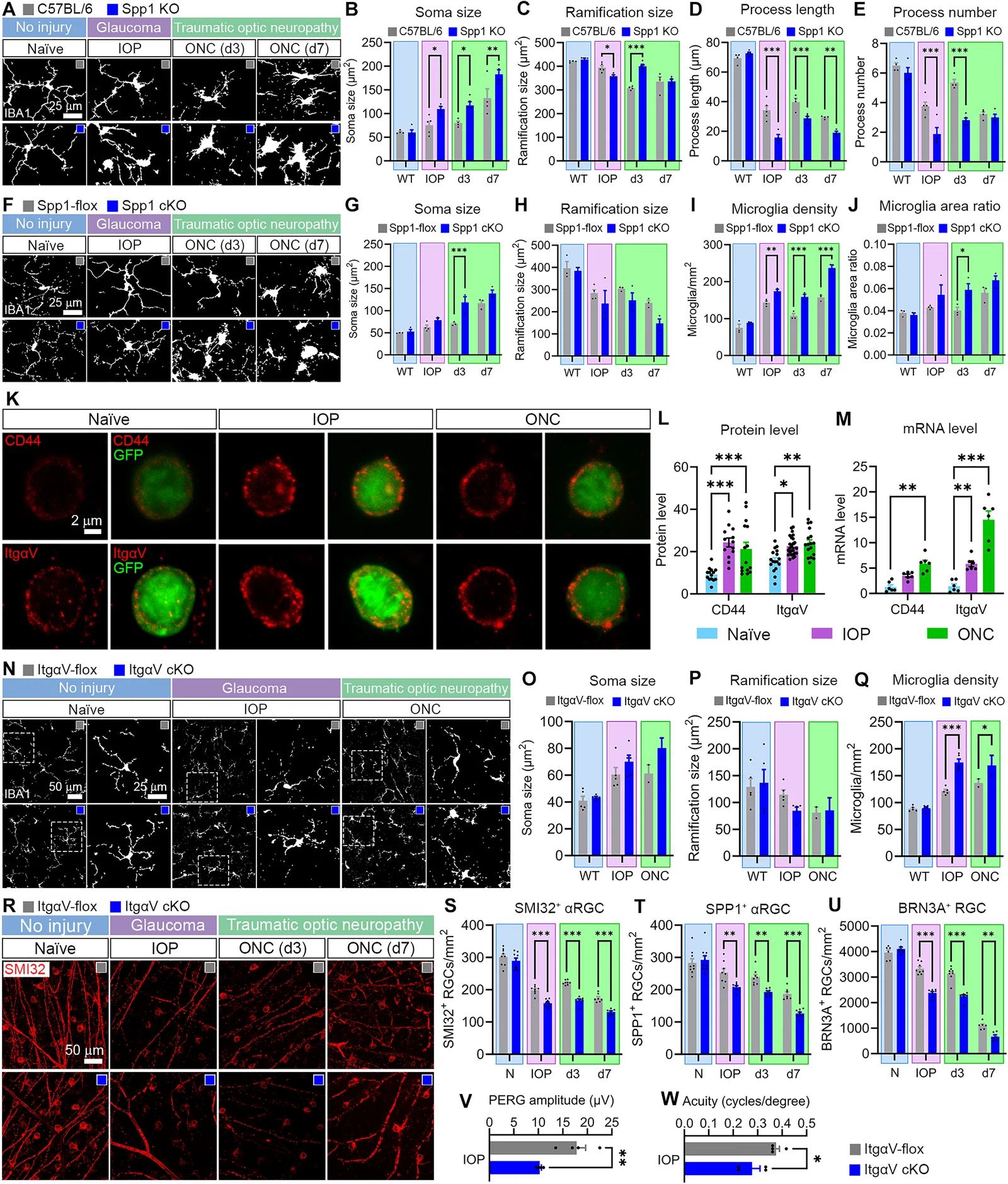
Spp1 deletion or microglial ItgαV loss exacerbates microglial activation in glaucoma and optic nerve injury. (**A**) IBA1 immunostaining of microglial morphology in WT and Spp1 KO retinas under naïve, 4 weeks after IOP elevation, and at days 3 and 7 after ONC. Note: These are magnified views of the dashed white box regions in fig. S3A. (**B** to **E**) Microglial soma size (B), ramification size (C), process length (D), and process number (E) in WT and Spp1 KO retinas (*n* = 4–5 mice). (**F**) IBA1^+^ microglia in Spp1-flox and Spp1 cKO retinas under naïve, IOP, and ONC conditions. Note: These are magnified views of the dashed white box regions in fig. S4A. (**G** to **J**) Microglial soma size (G), ramification size (H), density (I), and area ratio (J) in Spp1-flox and Spp1 cKO retinas (*n* = 3–4 mice). (**K** to **M**) FACS-sorted GFP^+^ microglia from CX3CR1-GFP mice for SPP1 receptors, CD44, and ItgαV at 4 weeks after IOP and day 7 after ONC (K), with quantification of protein (L) (*n* = 13–25 cells) and mRNA levels (M) (*n* = 6 mice). (**N** to **Q**) IBA1^+^ microglia (N) and quantification of soma size (O), ramification size (P), and density (Q) in ItgαV-flox and microglia-specific ItgαV cKO retinas after IOP and ONC (*n* = 2–5 mice). (**R** to **U**) SMI32 labeling (R) and quantification of SMI32^+^ αRGCs (S), SPP1^+^ αRGCs (T), and BRN3A^+^ non-αRGCs (U) in ItgαV-flox and ItgαV cKO retinas (*n* = 6–9 mice). (**V** and **W**) PERG amplitude (V) and visual acuity (W) at 4 weeks after IOP elevation (*n* = 4 mice). Statistical analyses: two-way ANOVA [(B) to (E), (G) to (J), (L) and (M), (O) to (Q), and (S) to (U)] and unpaired two-tailed t-test [(V and (W)]. *P < 0.05, **P < 0.01, ***P < 0.001. Data are mean ± SEM.

We found that retinal microglia in Spp1 KO mice are prone to increased reactivity. Microglia reactivity, characterized by an increase in soma size, retraction of the higher-order processes, and an “amoeboid” shape of the cells, is expected after injury to the retina or optic nerve (*30*, *31*). SPP1 was not detectable in retinal microglia at baseline or after injury. There was no morphological difference between microglia from wild-type and Spp1 KO retinas under physiological conditions. However, after glaucomatous and traumatic optic nerve injury, Spp1 KO microglia showed increased signs of activation (Fig. 2A and fig. S3), which were apparent in soma size, ramification size, and the length and number of processes (Fig. 2, B to E). The same held for the αRGCs-specific cKO mice (Fig. 2, F to J and fig. S4). However, the global Spp1 KO strain and the αRGCs-specific cKO showed increased overall ganglion cell loss, which presumably would lead to a greater accumulation of cellular debris. The increase in microglial reactivity may, therefore, reflect a response to ganglion cell death rather than an SPP1 effect on the microglial cells themselves. We sought to distinguish between these possibilities by manipulating SPP1 receptor expression on microglia.

### Microglial deletion of ItgαV also increases RGC vulnerability

SPP1 binds to integrin receptors, most commonly containing Integrin-αV (ItgαV, also known as CD51) and Integrin-β3, and to the glycoprotein CD44 (*32*, *33*). We tested which of the SPP1 receptors is expressed in retinal microglia using single-cell immunostaining and FACS (fig. S5, A and B). Most microglia expressed ItgαV; about 7% were also positive for CD44 (Fig. 2K) in FACS-sorted single microglia. ItgαV and CD44 (in the cells that express CD44) were upregulated on the protein and mRNA level after an elevation of IOP and optic nerve crush injury (Fig. 2, L and M). As the major SPP1 receptor on microglia was ItgαV, we created a microglia-specific deletion of ItgαV strain (ItgαV cKO) by crossing ItgαV-flox mice with Cx3cr1-cre. Compared to the cre^−^ controls, ItgαV cKO microglia showed increased morphological signs of activation, though this effect was significant for only some of the parameters we measured (Fig. 2, N to Q and fig. S5, C to I). Importantly, ItgαV cKO mice lost more αRGCs and non-αRGCs after injury than wild-type controls (Fig. 2, R to U and fig. S5, J and K). The increased vulnerability was reflected in worse functional outcomes in pERG and visual acuity (Fig. 2, V and W), suggesting that microglia increase their responsiveness to SPP1 during injury by up-regulating ItgαV.

Together, these findings support the hypothesis that increased SPP1 expression in αRGCs after injury serves as a defense mechanism that benefits not only the αRGCs themselves but also other non-αRGCs in the retina.

### Autophagy and debris clearance pathways are downregulated in Spp1 KO microglia

**Fig. 3.**
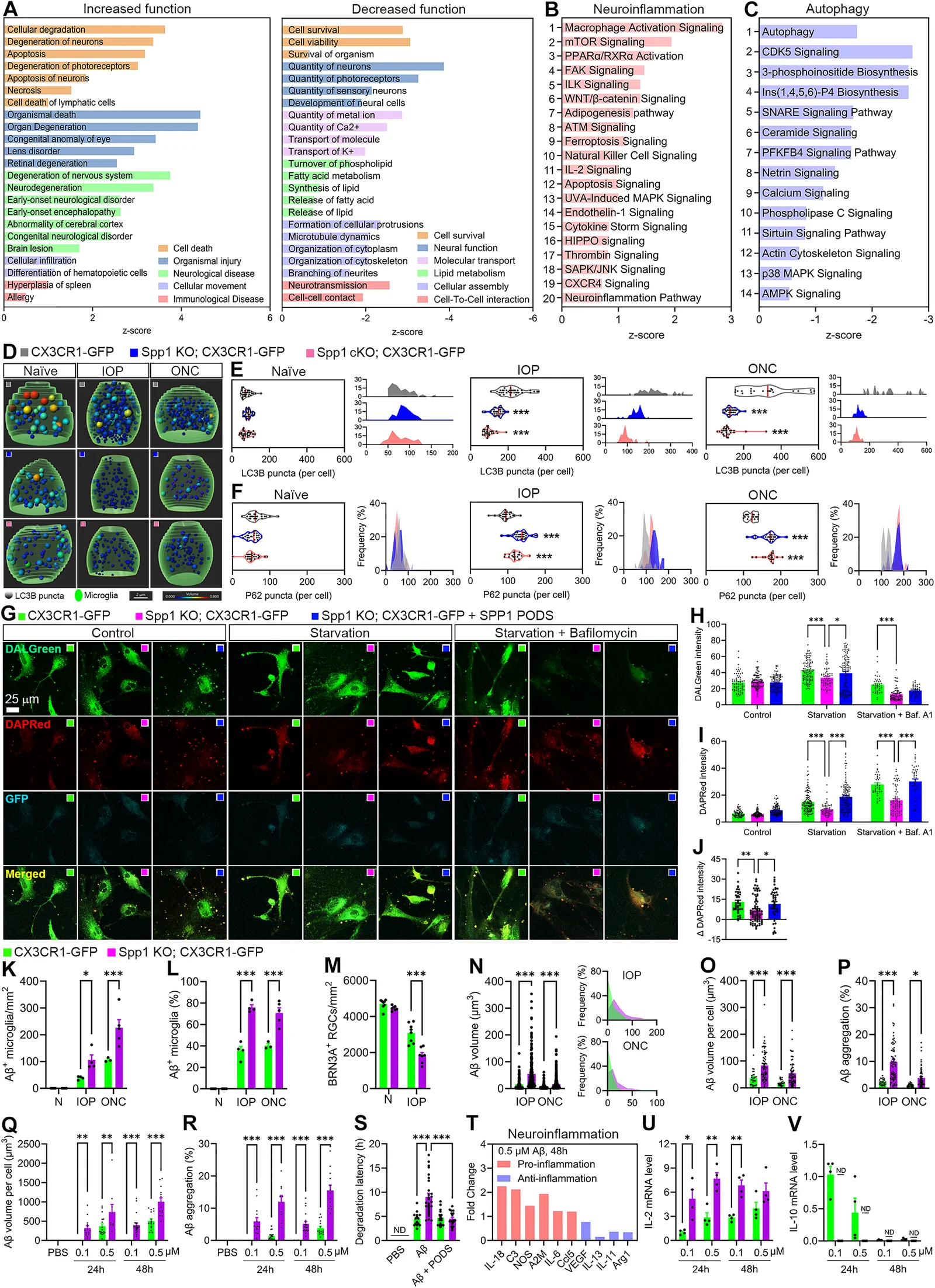
Spp1 deletion disrupts microglial autophagy and impairs debris clearance. (**A**) RNA-seq of retinal microglia from Spp1 KO; CX3CR1-GFP versus CX3CR1-GFP mice at 4 weeks after IOP elevation. (**B** and **C**) Gene Ontology analysis showing increased neuroinflammatory pathways (B) and reduced autophagy pathways (C). (**D**) LC3B immunostaining in FACS-sorted GFP^+^ microglia from WT, Spp1 KO, and Spp1 cKO mice under naïve, IOP, and day 7 ONC conditions, with Imaris-based reconstruction. (**E** and **F**) Quantification of LC3B (E) and P62 puncta (F) per microglia (*n* = 18–36 cells). (**G**) Autophagic flux imaging in WT and Spp1 KO microglia ± SPP1 PODS (50 ng/ml) using DALGreen/DAPRed probe system under basal, starvation, and starvation plus bafilomycin A1 conditions. (**H** to **J**) Quantification of DALGreen (H), DAPRed intensities (I), and autophagic flux index (Δ DAPRed) (J) (*n* = 39–126 cells). (**K** to **P**) Aβ^+^ microglial density (K) and percentage (L) (*n* = 3–5 mice), BRN3A^+^ RGC density (M) (*n* = 7–8 mice), intracellular Aβ volume (N) (*n* = 135–1001 puncta), and total Aβ per microglia (O) and aggregation (P) (*n* = 25–70 cells) in WT and Spp1 KO microglia across naïve, IOP, and ONC conditions. (**Q** to **R**) In vitro Aβ uptake (Q) and aggregation (R) in WT and Spp1 KO microglia (0.1 and 0.5 μM; 24 and 48 h; *n* = 9–21 cells). (**S**) Live-cell imaging of Aβ degradation latency in WT and Spp1 KO microglia ± SPP1 PODS (*n* = 15–28 cells). (**T** to **V**) Cytokine profiling (T) and qPCR of IL-2 (U) and IL-10 (V) expression after Aβ exposure (*n* = 4). Statistical analyses: one-way ANOVA [(E), (F), (J), and (S)] and two-way ANOVA [(H), (I), (K) to (P), (Q), (R), (U), and (V)]. *P < 0.05, **P < 0.01, ***P < 0.001. Data are mean ± SEM.

To explore the functional consequences of SPP1 loss on microglia, we crossed mice heterozygous for the expression of Cx3cr1-GFP with Spp1 KO animals. Elevated IOP was induced by microbead injection, and microglial cells were collected for RNA-seq from the retinas of KO and wild-type (with respect to Spp1) animals by FACS. Cell death and neuronal degeneration were prominent amongst the cellular functions predicted to be upregulated. In contrast, cell survival, lipid metabolism, cellular assembly, and cell interaction pathways were downregulated in Spp1 KO microglia (Fig. 3A). In addition, neuroinflammatory pathways and genes (e.g., Relb, Slc6a, Ptgs2, Pla2g6, Syk, Ccl5) were strongly activated in the Spp1 KO, indicating a neurotoxic phenotype of the Spp1 KO microglia (Fig. 3B and fig. S6, A to C). On the other hand, pathways related to autophagy and debris clearance were inhibited (Fig. 3C). We then asked whether the downregulation of these pathways may be secondary to a defect in phagocytosis. There was no indication that SPP1 regulates phagocytosis in microglia on the transcriptional level (fig. S6D). We then directly tested phagocytic activity in cultured wild-type and Spp1 KO microglia (fig. S6, E to H). Microglia from both groups showed equally robust phagocytic activity when challenged with pHrodo bioparticles (fig. S6, I and J). This suggests that Spp1 deficiency specifically inhibits microglial autophagy, but not phagocytosis.

### Direct evidence that autophagy and debris clearance are impaired in Spp1 KO microglia

To validate autophagy deficiency, we used FACS to sort freshly dissociated retinal microglia from wild-type, Spp1 KO, and αRGC-specific Spp1 cKO retinas after IOP elevation and ONC and stained autophagosomes using the markers LC3B and p62 (Sqstm1). We performed 3D reconstruction of microglia and their intracellular autophagosome structures using Imaris (Fig. 3D, for original images see fig. S7). LC3B is associated with autophagosomes, whereas p62 is a marker for impaired or defective autophagosomes (*34*, *35*). Under baseline conditions (no injury), microglia from all three genotypes contained approximately the same numbers of LC3B^+^ puncta per cell. However, after IOP elevation and optic nerve crush injury, the number of LC3B^+^ puncta was significantly increased in the wild-type, but this increase was blocked in the global Spp1 KO and the cKO cells (Fig. 3E and fig. S7A). The volume of single LC3B^+^ puncta and their total volume in each microglia were also smaller in the Spp1 KO and Spp1 cKO microglia and occupied a smaller fraction of the total cell volume (fig. S7, B to D). In contrast, p62^+^ puncta increased more strongly after injury in the Spp1 KO and Spp1 cKO microglia (Fig. 3F). Though more numerous, the p62^+^ puncta tended to be smaller in the KO cells than in the wild-type (fig. S7, E to H). The same effect was observed in a microglia-specific knockout of the SPP1 receptor ItgαV (fig. S7, I and J). Taken together, these data suggest that SPP1, though apparently not essential for the formation of autophagosomes under baseline conditions, is important for the upregulation and maintenance of autophagy after injury. Hence, the deletion of Spp1 from αRGCs or the deletion of the SPP1 receptor ItgαV from microglia can lead to impaired autophagy in glaucoma.

### SPP1 stimulates microglial autophagic flux

To functionally validate the role of SPP1 in microglial autophagy (Fig. 3G), we assessed autophagic flux using the DAPRed/DALGreen dual-fluorescent probe system (*36*, *37*). We compared three experimental conditions: normal condition (control), autophagy induction (starvation), and lysosomal inhibition (starvation plus bafilomycin A1). Under starvation-induced stress, WT microglia exhibited a robust increase in both DAPRed (marking autophagosomes and autolysosomes) and DALGreen (selectively marking acidified autolysosomes) puncta, indicating active biogenesis and successful lysosomal fusion (Fig. 3, H and I). In contrast, Spp1 KO microglia showed a significant reduction in both markers under starvation (Fig. 3, H and I), consistent with a deficit in autophagic induction.

To precisely quantify the rate of autophagosome biogenesis, we measured the accumulation of DAPRed puncta following treatment with bafilomycin to block lysosomal degradation (Fig. 3G). While WT microglia showed a massive accumulation of DAPRed-positive structures upon bafilomycin treatment, this response was markedly blunted in Spp1 KO microglia (Fig. 3I). This resulted in a significantly lower ∆ DAPRed (the net difference between bafilomycin-treated and untreated starvation conditions), confirming that the reduction in autophagic markers in KO cells is due to impaired formation (Fig. 3J). Importantly, treatment of Spp1 KO microglia with SPP1 POD significantly restored DAPRed intensity and effectively rescued the ∆ DAPRed flux index to levels comparable to WT (Fig. 3J). Together, these data demonstrate that SPP1 is important for the initiation of microglial autophagic flux and that exogenous SPP1 supplementation rescues the biogenesis defect.

### Accumulation of cellular debris in Spp1 KO microglia

After glaucomatous or traumatic damage to the optic nerve, amyloid-beta (Aβ) accumulates in the ganglion cell and nerve fiber layers of the retina (*38*, *39*). As the autophagy process can clear cellular aggregates (*40*, *41*), and Spp1 deletion causes autophagy deficiency, we then tested whether extracellular debris accumulates in microglia from Spp1 KO animals after optic nerve injury in vivo. In the retinas of naïve mice of either genotype, virtually no microglia with intracellular Aβ accumulations were found. After elevation of IOP or an optic nerve crush injury, the number of Aβ^+^ microglia cells and the percentage of all microglia that contained Aβ inclusions were significantly elevated in the Spp1 KO retinas compared to the wild-type (Fig. 3, K and L and fig. S8A), while RGC density was decreased (Fig. 3M and fig. S8B), suggesting that microglia with Aβ aggregation may be neurotoxic and harmful to RGC survival. In addition, the individual Aβ aggregates were significantly larger in the Spp1 KO microglia (Fig. 3, N and O) and occupied a larger fraction of the cell volume. (Fig 3P). In addition to dysfunctional Aβ clearance, Spp1 KO microglia also exhibited impaired clearance of apoptotic cell debris. We generated Spp1 cKO; CX3CR1-GFP mice by crossing αRGC-specific Spp1 cKO mice to CX3CR1-GFP; Spp1-flox mice, resulting in red labeling of αRGCs and green labeling of microglia. We then imaged microglia after IOP elevation and found that the percentage of microglia containing tdTomato^+^ debris from apoptotic cells was increased in Spp1 cKO microglia (fig. S9, A and B). We also tested the aggregation of α-synuclein and synapsin in single FACS-sorted microglia (fig. S9C). We did not detect mRNA expression of APP, α-synuclein, and synapsin in microglia, confirming that the protein aggregates originated from the extracellular microenvironment (fig. S9D). Finally, we verified that almost all genes involved in Aβ production, including APP, APP-binding proteins, and the secretases, were not altered in microglia in either genotype (fig. S9E). Taken together, these experiments indicate that αRGC-derived SPP1 is not involved in microglial phagocytosis but rather regulates a later step in debris clearance.

### In vitro demonstration of impaired microglial debris clearance

We treated microglia cultures with Aβ for 24 and 48 hours. As in the intact retina, cultured microglia from Spp1 KO mice showed increased accumulation of Aβ aggregates (Fig. 3, Q and R and fig. S8C). We then used live-cell imaging to study the time course of Aβ degradation in cultured microglia from wild-type and Spp1 KO mice. The cells were cultured in 12-well plates with medium containing 0.5 μM Aβ and imaged at 10-minute intervals for 24 hours. We observed cells phagocytosing Aβ aggregates and measured the degradation latency, i.e., the time required for the aggregate to completely disappear (movies S1 to S6). In Spp1 KO microglia, the degradation latency was 9.06 ± 0.73 hours, a two-fold increase over the 4.45 ± 0.36 hours in the wild-type (Fig. 3S and movies S3 and S4). Exogenous SPP1 protein (50 ng/ml SPP1 PODS) normalized the degradation latency in Spp1 KO microglia (4.51 ± 0.36 hours) but did not further increase the degradation latency in wild-type cells (Fig. 3S and movies S5 and S6).

### Aβ-treatment leads to increased production of neurotoxic factors in Spp1 KO microglia

We screened for expression of pro- and anti-inflammatory markers in cultured wild-type microglia after a challenge with Aβ (Fig. 3T). Several pro-inflammatory genes were moderately upregulated (e.g., IL-18 and C3), and some anti-inflammatory genes were downregulated (e.g., IL-13 and IL-11, Fig. 3T). However, the most significant effects were observed on IL-2 and IL-10, which were then analyzed in more detail. The pro-inflammatory factor IL-2 was found to be upregulated in Aβ-challenged wild-type microglia, and even more so in the Spp1 KO (Fig. 3U). In contrast, the anti-inflammatory factor IL-10 was strongly inhibited by Aβ in wild-type microglia and was completely undetectable in the Spp1 KO microglia (Fig. 3V). These data suggest that dysfunctional debris clearance leads to microglial debris accumulation, which in turn is associated with an inhibition of neuroprotective factor expression and an increase in neurotoxic factor expression.

To determine whether the observed transcriptional changes translated into altered protein expression, we employed a proteome profiler mouse cytokine array to simultaneously screen 40 cytokines across experimental groups (fig. S10A). In response to Aβ treatment, WT microglia exhibited a robust inflammatory profile, characterized by the presence of multiple pro-inflammatory mediators, including ICAM-1, IL-1α, IL-1β, CXCL10, CXCL1, M-CSF, CCL2, CXCL2, CCL5, and TIMP-1 (fig. S10, A and B). In contrast, Spp1 KO microglia displayed an upregulated inflammatory signature compared to WT cells under Aβ-challenged conditions (fig. S10, A and B). Supplementation with exogenous SPP1 PODS partially restored the cytokine profile in Spp1 KO microglia toward that of WT cells, supporting that SPP1 is a key regulator of the microglial inflammatory response. Despite the overall robustness of the cytokine array, IL-2 and IL-10 are not reliably detected across conditions (*42*–*44*). Therefore, we assessed their expression separately by immunostaining in cultured microglia, which revealed detectable levels of both cytokines (fig. S10, C to F). IL-2 expression was minimal under basal conditions but was upregulated in WT microglia following Aβ treatment, with a further increase observed in Spp1 KO cells (fig. S10, C and D). In contrast, IL-10 levels were significantly reduced in Spp1 KO microglia compared to WT controls (fig. S10, E and F). Collectively, these findings demonstrate that SPP1 broadly regulates microglial cytokine signaling in response to Aβ.

### Microglia with impaired debris clearance drive RGC degeneration

**Fig. 4.**
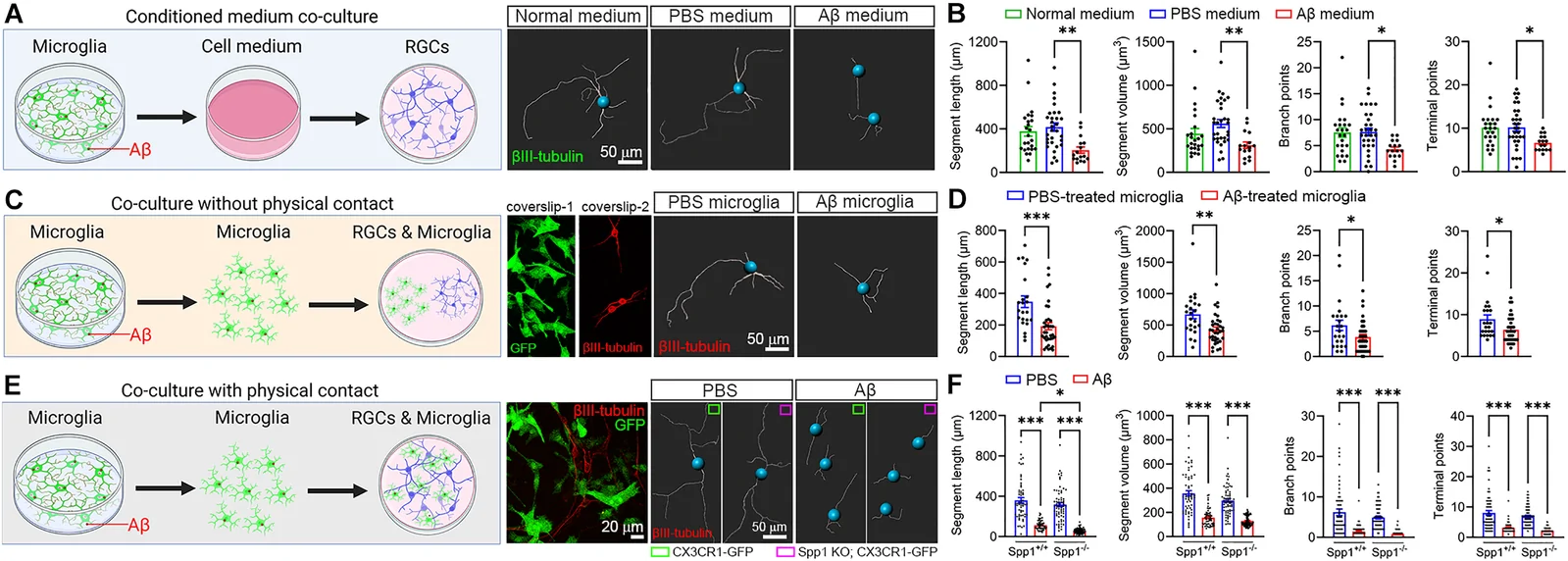
Defective microglial debris clearance induces neurotoxicity. (**A**) Schematic of conditioned medium co-culture of Aβ-treated microglia with primary RGCs. (**B**) Quantification showing reduced RGC neurite length, volume, branch points, and terminal points when cultured in medium from Aβ-treated microglia compared to PBS controls (*n* = 16–31 cells). (**C**) Indirect microglia–RGC co-culture without physical contact. (**D**) RGCs co-cultured with Aβ-treated microglia exhibited reduced neurite complexity compared with controls (*n* = 23–35 cells). (**E**) Direct co-culture of microglia and RGCs with physical contact. (**F**) Aβ-treated WT (Spp1^+^/^+^) microglia significantly reduced RGC neurite complexity, and Spp1 KO (Spp1^−^/^−^) microglia caused even greater neurite loss (*n* = 54–98 cells). Statistical analyses: one-way ANOVA [(B) and (F)] and unpaired two-tailed t-test (D); *P < 0.05, **P < 0.01, ***P < 0.001. Data are mean ± SEM. Diagram (A) was created in BioRender. Li, S. (2026) https://BioRender.com/0u56lw7. Diagram (C) was created in BioRender. Li, S. (2026) https://BioRender.com/kuqoumr. Diagram (E) was created in BioRender. Li, S. (2026) https://BioRender.com/yeftaab.

We cultured wild-type microglia, challenged them with 0.5 μM Aβ for 48 h, and tested their neurotoxic potential on cultured RGCs. Three different paradigms were used. In the first step, we transferred microglia supernatant (1 mL) to the wells containing RGCs (Fig. 4, A and B). In the second, microglia were grown on a glass coverslip, challenged with Aβ, and then the coverslip was transferred to the RGC culture. In this setup, no direct cell-to-cell contact between microglia and RGCs occurred (Fig. 4, C and D). Thirdly, cultured microglia were challenged with Aβ, trypsinized, and co-cultured with direct contact to RGCs (Fig. 4, E and F). The morphological complexity of ganglion cells’ neurites, including neurite length, volume, branch point number, and terminal point number, was evaluated microscopically and quantified using Imaris. In all cases, but most significantly in the co-cultures with direct contact between microglia and RGCs, Aβ-treated microglia significantly inhibited the growth and ramification of RGC neurites (Fig. 4, B, D, and F).

We then tested whether microglia from Spp1 KO mice would have an inhibitory effect on RGC neurite growth, in addition to that observed in wild-type cells. As the strongest effect was observed in the co-culture with direct cell-to-cell contacts, we chose this paradigm to compare wild-type to Spp1 KO microglia (Fig. 4E). Under these conditions, RGC neurite length was decreased more significantly compared to the wild-type microglia, and the other parameters (neurite volume, number of branch points, and terminal points) showed a downward trend (Fig. 4F).

### SPP1 deletion decreases selective autophagy receptors

We next turned to the mechanism of SPP1 activity in microglial debris clearance. We used RNA-seq expression data from wild-type and Spp1 KO microglia to identify processes influenced by SPP1. Scavenger receptors, TLRs, and NLRs, several of which are potential Aβ binding proteins, were not affected by Spp1 KO, and neither were genes involved in extracellular proteolytic degradation (fig. S11, A and B). There are two major cellular pathways for the removal of cellular components and debris: the ubiquitin-proteasome system and autophagy. The ubiquitin-proteasome system was not affected by Spp1 deletion (fig. S11C). Autophagy removes unnecessary or dysfunctional cellular components via a lysosome-dependent mechanism (*40*, *41*). Autophagy consists of several sequential steps: (1) induction, (2) nucleation and phagophore formation, (3) elongation and autophagosome formation, (4) fusion and autolysosome formation, and (5) degradation of cargo in the autolysosome (*45*, *46*). There are three types of autophagy: macroautophagy, microautophagy, and chaperone-mediated autophagy (CMA) (*47*). Even though some genes (St13, Hsp90ab1, and Dnajb1) involved in chaperone-mediated autophagy were altered in Spp1 KO microglia (fig. S11D), Aβ is not expected to go through degradation via chaperone-mediated autophagy due to the lack of the necessary KFERQ motif (*48*, *49*). In addition, microautophagy in microglia was not affected by Spp1 deletion (fig. S11E).

**Fig. 5.**
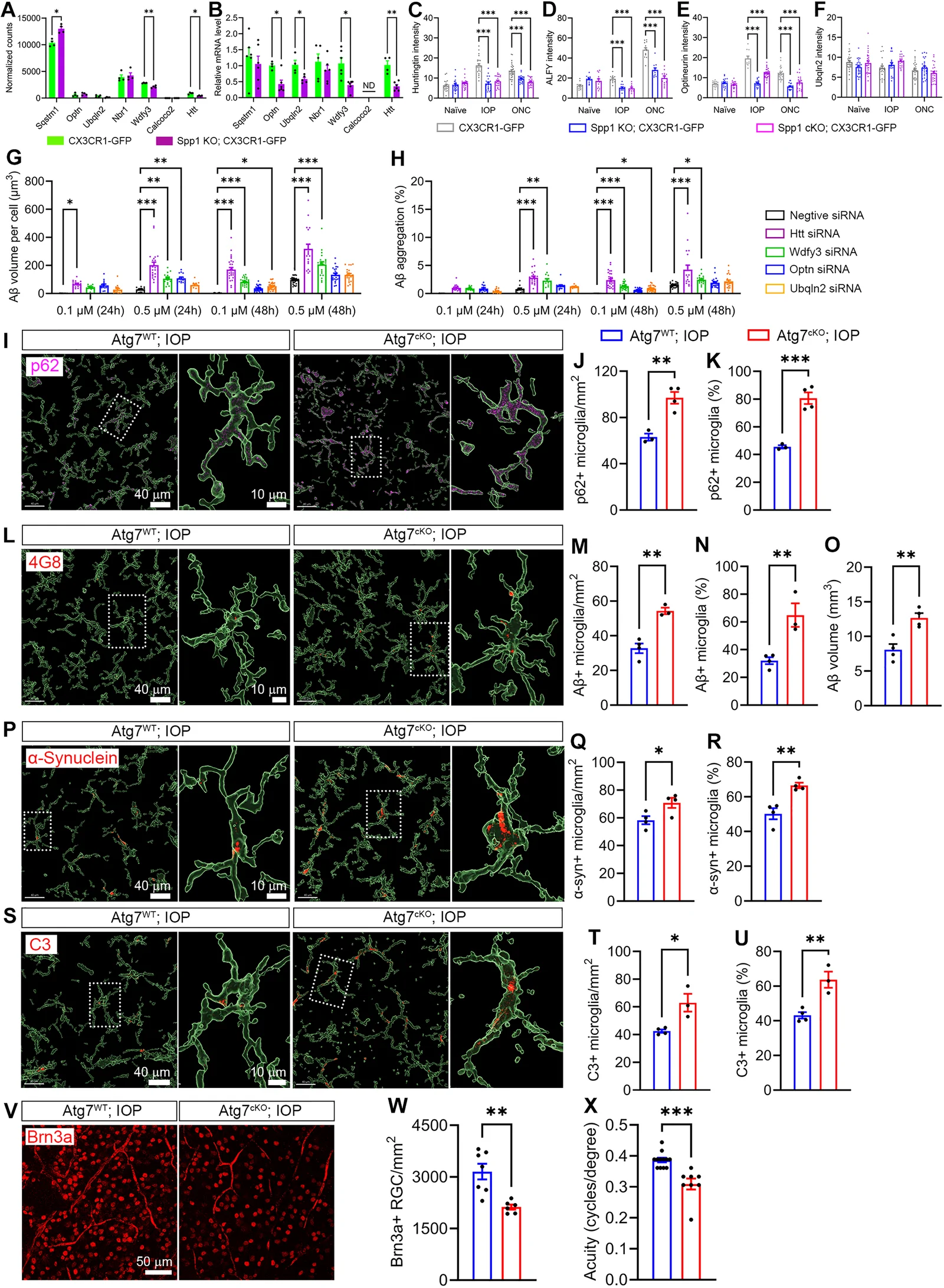
Microglial autophagy deficiency drives abnormal debris clearance, RGC degeneration, and vision loss. (**A**) Gene expression of autophagy-selective receptors in retinal microglia from CX3CR1-GFP and Spp1 KO; CX3CR1-GFP mice (*n* = 4 mice). (**B**) qPCR validation in FACS-sorted retinal microglia (*n* = 4–6 mice). (**C** to **F**) Huntingtin (Htt) (C), ALFY (Wdfy3) (D), optineurin (Optn) (E), and Ubqln2 (F) protein levels in FACS-sorted microglia from CX3CR1-GFP, Spp1 KO; CX3CR1-GFP, and Spp1 cKO; CX3CR1-GFP mice (naïve, IOP, and ONC; Huntingtin *n* = 30–34 cells; ALFY 11–14; optineurin 11–32; Ubqln2 20–30). (**G** and **H**) Intracellular Aβ volume (G) and aggregation (H) in WT microglia treated with Htt, Wdfy3, Optn, or Ubqln2 siRNAs and Aβ_1–42_-555 (*n* = 11–30 cells). (**I**) 3D reconstructions of IBA1^+^ microglia with p62 accumulation in Atg7-flox (Atg7^WT^; IOP) and Atg7^cKO^ mice (Atg7^cKO^; IOP) after IOP. (**J** and **K**) p62^+^ microglia density (J) and proportion (K) in Atg7^WT^; IOP and Atg7^cKO^; IOP retinas (*n* = 3–4 mice). (**L** to **O**) Immunostaining for Aβ (L), Aβ^+^ microglia density (M), percentage (N), and intracellular Aβ volume (O) in Atg7^WT^ and Atg7^cKO^ mice after IOP (*n* = 3–4 mice). (**P** to **R**) Immunostaining for α-synuclein (α-syn) (P), α-syn^+^ microglia density (Q), and percentage (R) in Atg7^WT^ and Atg7^cKO^ retinas (*n* = 4 mice). (**S** to **U**) Immunostaining for C3 in IBA1^+^ microglia (S), C3^+^ microglia density (T), and percentage (U) in Atg7^WT^ and Atg7^cKO^ retinas (*n* = 3–4 mice). (**V**) RGCs in Atg7^WT^ and Atg7^cKO^ mice after IOP. (**W**) RGC density (*n* = 6–7 mice). (**X**) Visual acuity (*n* = 8–12 mice). Statistical analyses: multiple unpaired t-test (B), two-way ANOVA [(C) to (E), (G), and (H)], and unpaired two-tailed t-test [(J), (K), (M to O), (Q), (R), (T), (U), (W), and (X)]. *P < 0.05, **P < 0.01, ***P < 0.001. Data are mean ± SEM.

However, via screening the five steps of macroautophagy, we found a clear effect of Spp1 deletion on selective autophagy receptors (Fig. 5A and fig. S11, F to M). Selective autophagy receptors help protein aggregates target and deliver into the autophagosome (*50*). Among these receptors, Htt (huntingtin), Wdfy3 (WD repeat and FYVE domain-containing 3, encoding the large scaffolding protein ALFY), Optn (optineurin), and Ubqln2 (ubiquilin-2) were downregulated on the mRNA level (Fig. 5B). For Htt, ALFY, and Optn, the downregulation was also apparent in immunostaining of isolated microglia from wild-type and Spp1 KO mice (Fig. 5, C to F and fig. S12, A to D). A similar result was also observed in ItgαV cKO microglia after IOP elevation (fig. S12, E to L). When autophagy deficiency was induced by inhibiting these selective receptors using siRNA (fig. S13, A and B), Aβ accumulated in cultured microglia (Fig. 5, G and H and fig. S13C). The effect was most pronounced for Htt, followed by Wdfy3. Taken together, these data indicate that Spp1 deletion specifically affects the selective autophagy receptors.

### Microglial autophagy deficiency drives RGC degeneration

We reasoned that if the inhibition of microglial autophagy that we observed in the Spp1 KO mice was indeed responsible for debris clearance deficiency and RGC loss, any manipulation that affects microglial autophagy and leads to the intracellular accumulation of debris should have a similar effect. We therefore deleted the essential autophagy gene Atg7 (*51*) in microglia by crossing Atg7-floxed mice with Cx3cr1-cre and elevated the IOP by microbead injection. Confocal image stacks of retinal microglia were taken and reconstructed using Imaris. As expected, after IOP elevation, Atg7 cKO microglia showed increased intracellular accumulation of p62 (indicative of autophagy deficiency), Aβ, α-synuclein, and complement C3 (a neurotoxic marker) compared to Atg7^WT^ littermates (Fig. 5, I to U). Atg7 cKO mice lost significantly more retinal ganglion cells after an elevation of IOP, which was reflected in lower visual acuity (Fig. 5, V to X). These data suggest that microglial autophagy deficiency drives microglial debris accumulation and RGC degeneration in glaucoma.

### Spp1 overexpression resolves microglial debris accumulation and prevents neurodegeneration

**Fig. 6.**
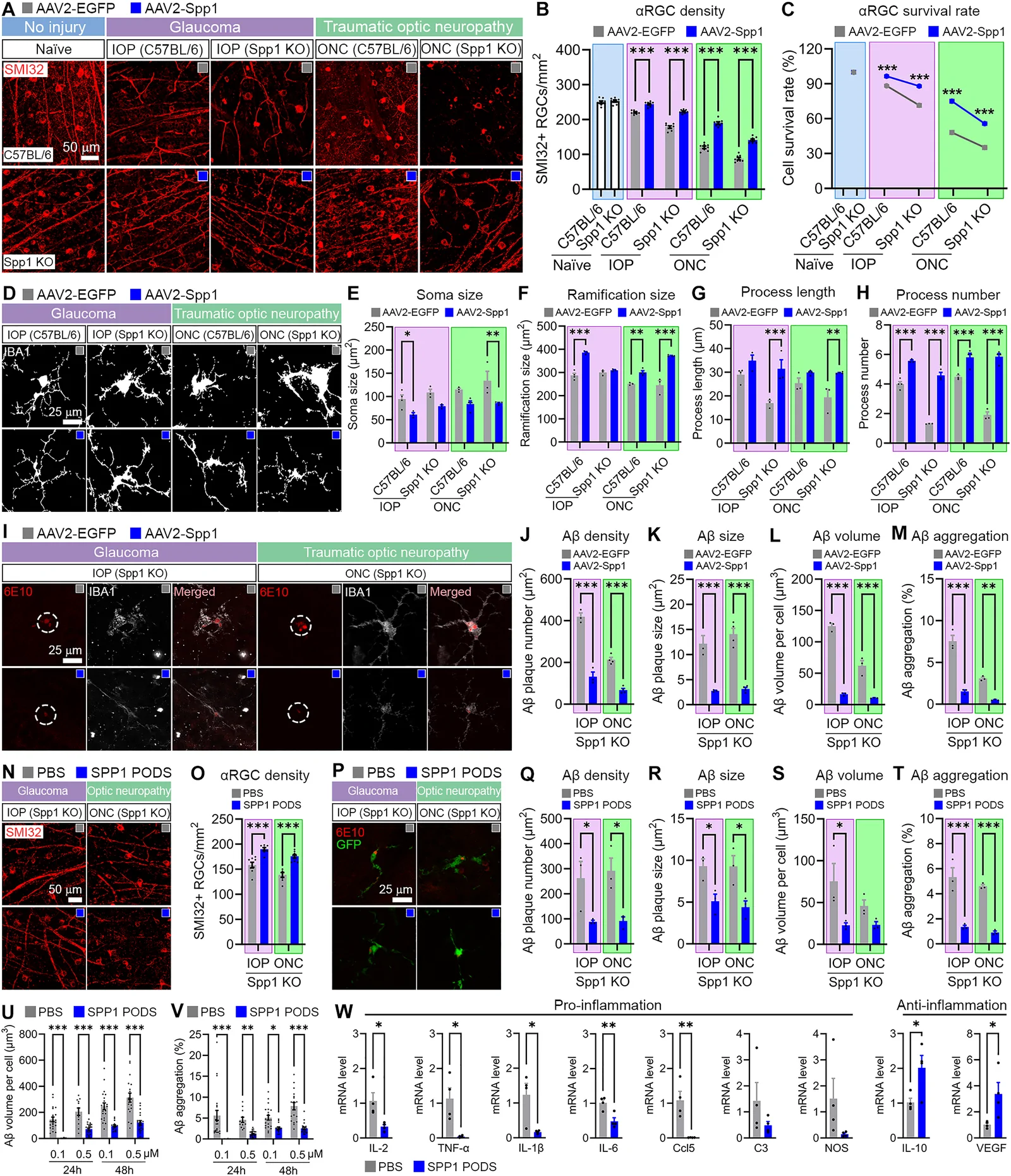
SPP1 overexpression restores microglial homeostasis and prevents RGC degeneration. (**A**) SMI32 staining showing αRGC protection in WT and Spp1 KO mice treated with AAV2-SPP1 or control AAV2-EGFP after IOP elevation or ONC. (**B** and **C**) SMI32^+^ αRGC density (B) and survival rate (C) (*n* = 8 mice). (**D**) IBA1 immunostaining of microglia after AAV2-SPP1 treatment. (**E** to **H**) Microglial soma size (E), ramification size (F), process length (G), and process number (H) in WT and Spp1 KO mice treated with AAV2-Spp1 or AAV2-EGFP (*n* = 3–4 mice). (**I**) 6E10-labeled Aβ in microglia following AAV2-SPP1 treatment. (**J** to **M**) Aβ density (J), size (K), volume (L), and aggregation (M) in WT and Spp1 KO mice treated with AAV2-SPP1 or AAV2-EGFP (*n* = 3–4 mice). (**N** and **O**) SMI32-labeled αRGCs (N) and density (O) in Spp1 KO mice treated with SPP1 PODS or PBS after IOP elevation or ONC (*n* = 8 mice). (**P** to **T**) 6E10-labeled Aβ (P), Aβ density (Q), size (R), volume (S), and aggregation (T) in microglia from Spp1 KO; CX3CR1-GFP mice treated with SPP1 PODS after IOP elevation or ONC (*n* = 3 mice). (**U** and **V**) Aβ volume (U) and aggregation (V) in cultured Spp1 KO microglia treated with SPP1 PODS and Aβ_1–42_-555 (*n* = 12–29 cells). (**W**) qPCR analysis of pro- and anti-inflammatory factors in Spp1 KO microglia treated with SPP1 PODS and Aβ (*n* = 4). Statistical analyses: two-way ANOVA [(B), (C), (E) to (H), (J) to (M), (O), (Q) to (T), (U), and (V)] and unpaired two-tailed t-test (W). *P < 0.05, **P < 0.01, ***P < 0.001. Data are mean ± SEM.

Our data indicate that deletion of Spp1 from RGCs (or its receptor ItgαV from microglia) leads to increased vulnerability of ganglion cells to injury and that this effect is mediated by a decrease in autophagy in the retinal microglia. We next tested whether the opposite would also be true and supplied SPP1 either by AAV-mediated transfection or by administration of a slow-release form of the protein (SPP1 PODS). AAV-GFP (without the Spp1 gene) or PBS (without PODS) served as controls. AAV-mediated SPP1 overexpression in wild-type retinas improved αRGC survival in the microbead model and after optic nerve crush (Fig. 6, A to C). To ensure that the effect was due to the exogenous SPP1 rather than endogenous SPP1 secretion from RGCs or glial cells, we also used Spp1 KO mice (Fig. 6, A to C). Spp1 KO mice lost more αRGCs than wild-type C57bl/6 mice in both injury models, but SPP1 overexpression was significantly protective of αRGC survival in both strains (Fig. 6, B and C). In the same retinas, morphological signs of microglia activation were attenuated. This was apparent in soma size, ramification size, process length, process number, and absolute cell numbers (Fig. 6, D to H and fig. S14, A to C).

We then tested whether SPP1 overexpression reduced the accumulation of Aβ in microglia after IOP elevation or optic nerve crush (Fig. 6I and fig. S14, D and E). AAV-mediated overexpression of SPP1 resulted in a significant reduction of the number and size of Aβ aggregates found in microglia (Fig. 6, J to M), consistent with the interpretation that SPP1 increases the efficiency of autophagy and debris clearance. A similar effect was observed with the SPP1 protein (SPP1 PODS), which also decreased αRGC loss (Fig. 6, N and O) and Aβ accumulation in microglia (Fig. 6, P to V and fig. S14, F to H). The effect was receptor-mediated as it was inhibited by ItgαV siRNA (fig. S14, I to M). By qPCR, exogenous SPP1 reduced the expression of pro-inflammatory genes such as IL-2, TNF-α, and IL-1β, and it stimulated the expression of the anti-inflammatory genes VEGF and IL-10 (Fig. 6W).

### SPP1 expression in glaucomatous human and macaque retinas

**Fig. 7.**
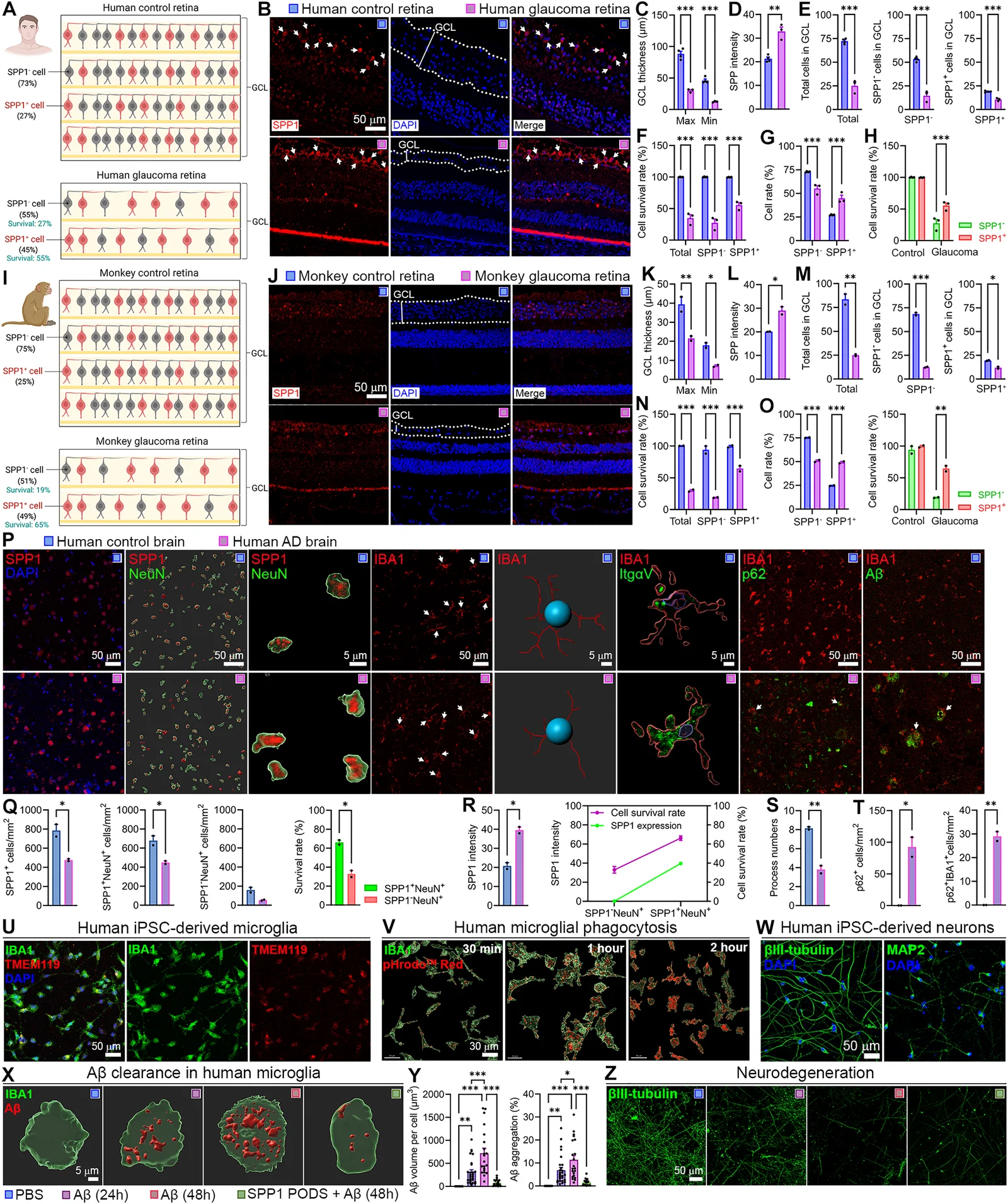
SPP1 signaling correlates with glaucomatous and Alzheimer’s pathology in human and nonhuman primates. (**A** and **B**) Schematic (A) and representative SPP1 immunostaining (B) in retinas from control donors and patients with glaucoma. (**C** to **H**) Quantification of ganglion cell layer (GCL) thickness (C), SPP1 levels (D), total and SPP1^+^/SPP1^−^ cell numbers (E), survival rates (F), SPP1^+^ cell proportion (G), and survival comparison (H) (n = 4 control and 3 glaucoma donors). (**I** and **J**) Schematic (I) and SPP1 staining (J) in nonhuman primate (NHP) control and experimental glaucomatous (EG) retinas. (**K** to **O**) Quantification of GCL thickness (K), SPP1 expression (L), cell numbers (M), survival rates (N), and SPP1^+^ versus SPP1^−^ survival (O) in NHPs (*n* = 2 control and 2 EG NHPs). (**P** to **T**) Immunostaining and quantification in human frontal cortex from control and Alzheimer’s disease (AD) donors (P), including SPP1, NeuN, IBA1, ItgαV, p62, and Aβ, SPP1^+^ cell and neuronal densities, survival of SPP1^+^/SPP1^−^ neurons (Q), SPP1 expression (R), microglial process number (S), and p62^+^ cell densities (T) (*n* = 2 control and 2 AD donors). (**U** to **W**) Characterization of human iPSC-derived microglia (U), their phagocytic activity (V), and neurons (W). (**X** and **Y**) iPSC-derived microglia treated with Aβ ± SPP1 PODS (50 ng/ml), with 3D reconstruction (X) and quantification of intracellular Aβ volume and aggregation (Y) (n = 15–27 cells). (**Z**) Co-culture of treated microglia with iPSC-derived neurons, followed by neuronal βIII-tubulin staining. Statistical analyses: two-way ANOVA [(C), (F) to (H), (K), (N), and (O)] unpaired two-tailed t-test [(D), (E), (L), (M), and (Q) to (T)] and one-way ANOVA (Y). *P < 0.05, **P < 0.01, ***P < 0.001. Data are mean ± SEM. Diagram (A) was created in BioRender. Li, S. (2026) https://BioRender.com/eoojgxk. Diagram (I) was created in BioRender. Li, S. (2026) https://BioRender.com/ipzu0f9.

SPP1^+^ ganglion cells are more numerous in human retinas than they are in the mouse (Fig. 7, A and B). Possibly, these cells are parasol ganglion cells, the equivalent of the αRGCs in the mouse and Y cells in the cat retina (*52*–*54*). The thickness of the ganglion cell layer is reduced in human glaucomatous retinas (Fig. 7C and fig. S15A), and, similar to mouse RGCs, the intensity of SPP1 immunolabeling is increased (Fig. 7D). SPP1 labeling does not colocalize with astrocyte or microglia markers, but SPP1^+^ cells also express the neuronal marker NeuN, indicating that in human as in mouse retinas, SPP1 expression is restricted to ganglion cells (fig. S15, B to D). Retinal microglia show immunolabeling for the SPP1 receptor ItgαV (fig. S15E). The total number of cells in the ganglion cell layer was reduced, as observed for both SPP1^+^ and SPP1^−^ cells (Fig. 7, E and F). However, the SPP1^+^ RGCs appeared more resistant to degeneration. This led to an increase in the relative abundance of SPP1^+^ RGCs in glaucomatous retinas (Fig. 7G) and a higher cell survival rate (Fig. 7H). We measured the soma size of IBA1^+^ microglia as a morphological sign of activation. There was a clear negative correlation between SPP1^+^ cell survival rate and microglia activation (fig. S15, G to H), suggesting that, similarly to the situation in mice, SPP1 may inhibit microglia activation in human retinas.

Macaque retinas, anatomically very similar to those of humans, also have numerous SPP1^+^ cells in the ganglion cell layer (Fig. 7, I and J and fig. S15I). In a laser-induced experimental glaucoma model, the macaque retinas also show a decrease in ganglion cell layer thickness (Fig. 7K) and an increase in SPP1 expression (Fig. 7L). Both SPP1^+^ and SPP1^−^ cells were decreased in the macaque retinas with experimental glaucoma, while SPP1^+^ cells had a higher survival rate than SPP1^−^ cells (Fig. 7, M to P), a similar finding to human glaucoma retinas.

### SPP1 signaling is preserved in the human Alzheimer’s disease brain

We next examined SPP1 signaling in the human brain with Alzheimer’s Disease (AD). Histological analysis of post-mortem frontal cortex samples from two patients with AD and age-matched controls revealed SPP1 colocalization with neurons (Fig. 7P). Both SPP1^+^ and SPP1^−^ neuron numbers were reduced in the AD frontal cortex (Fig. 7Q), but SPP1^+^ neurons exhibited higher survival rates (Fig. 7Q). SPP1 expression was upregulated by AD pathology and positively correlated with SPP1^+^ neuron survival (Fig. 7R). In the frontal cortex of control donors, microglia displayed thin, ramified processes characteristic of a resting state and expressed ItgαV (Fig. 7P). In contrast, AD microglia showed increased ItgαV staining and a globular, amoeboid morphology indicative of activation (Fig. 7S). AD cortex also exhibited elevated numbers of p62^+^ cells and p62^+^ microglia (Fig. 7T), consistent with impaired microglial autophagy, and microglial density was higher near Aβ deposits (Fig. 7P). These findings are preliminary due to the small sample sizes, but they suggest that AD pathology may induce neuronal SPP1 expression and microglial ItgαV upregulation, possibly implicating SPP1 signaling in human AD pathogenesis.

### SPP1 promotes microglial Aβ clearance and neuroprotection in human iPSC models

To directly test the role of SPP1 in human microglia, we employed co-cultures of human iPSC-derived microglia and neurons that model the glaucomatous and AD environment (Fig. 7U). Human iPSC-derived microglia exhibited typical ramified morphology, expressed IBA1 and TMEM119, and displayed normal phagocytic activity (Fig. 7, U and V). Human iPSC-derived neurons showed extensive neurite outgrowth and expressed pan-neuronal markers βIII-tubulin and MAP2 (Fig. 7W). Exposure of microglia to Aβ resulted in intracellular Aβ accumulation (Fig. 7, X and Y), consistent with our in vivo observations. Treatment with SPP1 PODS markedly enhanced Aβ clearance in human microglia (Fig. 7, X and Y). In neuron–microglia co-cultures, Aβ-loaded microglia induced neuronal degeneration, evidenced by shortened neurites, whereas SPP1 PODS treatment prevented this degeneration (Fig. 7Z and fig. S15J). Thus, SPP1 promotes microglial clearance of Aβ aggregates and preserves neuronal integrity in human systems, highlighting a conserved neuroprotective role for SPP1 signaling.

## DISCUSSION

**Fig. 8.**
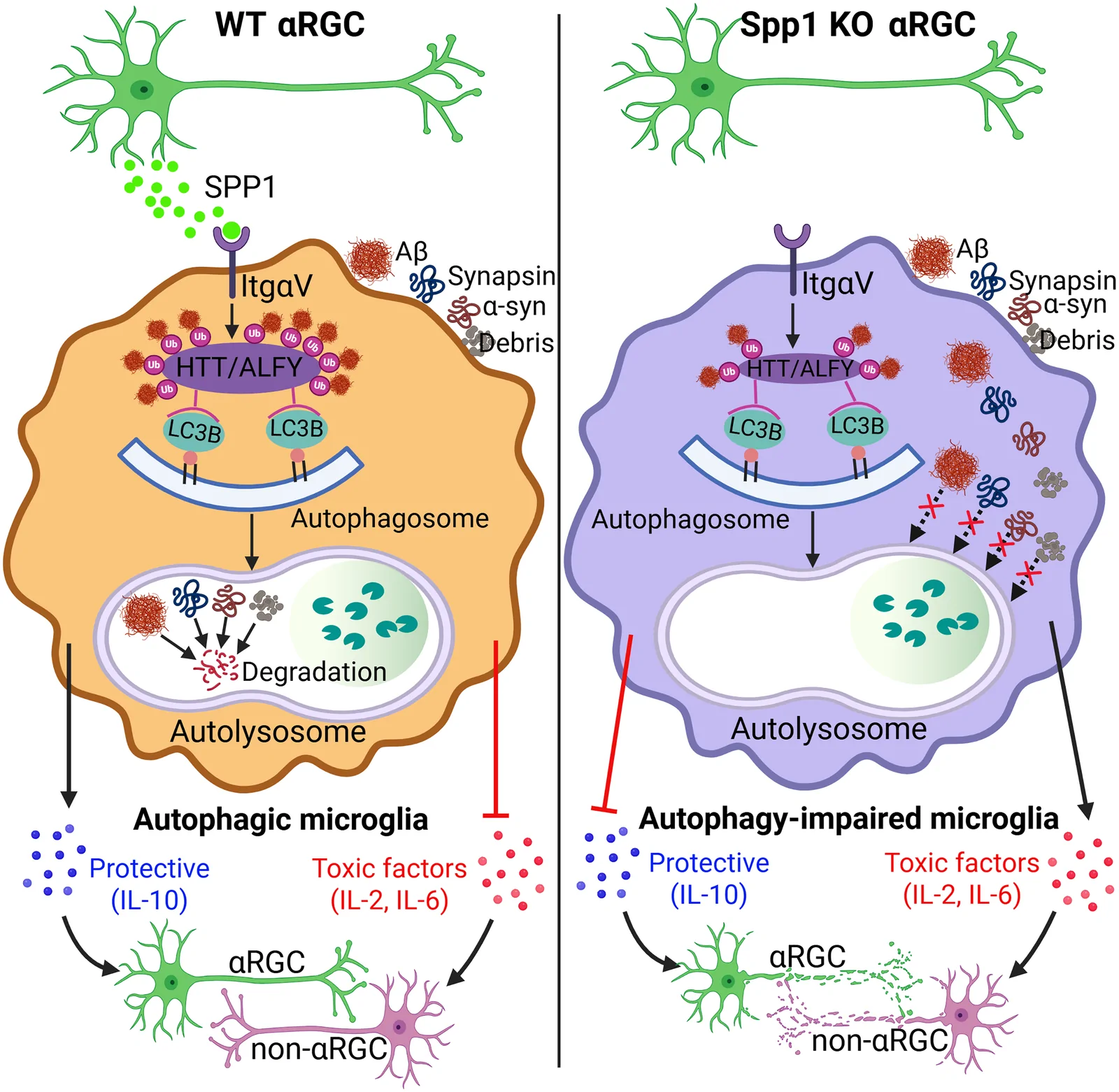
αRGC-derived SPP1 instructs microglial autophagy to promote neuroprotection. Following neurodegenerative injury, αRGCs upregulate and secrete secreted phosphoprotein 1 (SPP1), which signals through microglial ItgαV to promote transcriptional and morphological reprogramming. In wild-type conditions, SPP1 enhances microglial autophagy by promoting ubiquitin-dependent cargo recognition through HTT/ALFY and LC3B-associated autophagosome formation. This pathway supports the intracellular processing and lysosomal degradation of internalized protein aggregates and neuronal debris, including Aβ-containing cargo, α-synuclein, and synapsin. The resulting autophagic microglial state is associated with increased neuroprotective mediators, such as IL-10, and reduced pro-inflammatory factors, such as IL-2 and IL-6, thereby preserving neuronal function and limiting neurodegeneration. In contrast, loss of SPP1 impairs microglial autophagy, leading to stalled cargo processing, defective degradation of aggregates and debris, and accumulation of internalized neuronal cargo. This autophagy-impaired state promotes toxic factor release and diminishes neuroprotection, ultimately contributing to neuronal dysfunction and degeneration. The image was created in BioRender. Li, S. (2026) https://BioRender.com/e7hhfl9.

Alpha RGCs are present in all mammalian retinas (*26*, *55*–*57*), where they are involved in detecting changes in luminance and approaching (or looming) objects rather than mediating high-acuity vision (*58*). Due to their rapid response characteristics and fast axonal conduction, they may mediate an alarm response or direct visual attention towards an approaching threat (*59*). Here we present evidence that αRGCs, in addition to their function in vision, also play a role in protecting the retina against injury. This response is dependent on SPP1, but it protects not only the αRGCs themselves but also neighboring RGCs that are not intrinsically positive for SPP1. Microglia are necessary for this neuroprotective effect, as both the conditional deletion of Spp1 from αRGCs or the deletion of one of its receptors, ItgαV, from microglia disrupts it, establishing a neuron-to-microglia signaling axis critical for injury defense. A schematic of the proposed mechanism is given in Fig. 8. None of this is to say that this is the only mechanism by which SPP1 protects RGCs. Cell-autonomous effects have been demonstrated (*18*), and as a secretory protein, SPP1 may act on other neuronal or glial cells in the retina or the optic nerve by mechanisms independent of the one we described here.

The loss of neuronal cell populations is the central feature of neurodegenerative diseases. In glaucoma, the visual dysfunction is caused by the loss of retinal ganglion cells and their axons (*8*). As in other neurodegenerative diseases, inflammation, mitochondrial dysfunction, oxidative stress, or growth factor deprivation were found to contribute to RGC degeneration (*60*). In some forms of glaucoma, especially those associated with mutations in myocilin, optineurin, and lysyl oxidase 1, protein misfolding and endoplasmic reticulum stress are involved in the pathology (*61*, *62*). Though plaques are not observed in glaucoma, amyloid beta and amyloid precursor protein are increased in the retina and the visual pathway in human glaucoma and animal models (*38*, *39*, *63*, *64*). It is yet unclear whether they are causally involved in the pathology. Extracellular Aβ, and other potentially neurotoxic proteins, such as α-synuclein and synapsin, are readily taken up and degraded by retinal microglia (*65*). In contrast to astrocytes, where SPP1 facilitates phagocytic activity (*23*), microglia showed no phagocytosis defect in the absence of SPP1. Within the limits of our assays, the phagocytosis of Aβ and other debris occurred as readily in microglia from Spp1 KO mice as it did in wild-type cells. After extracellular Aβ is internalized by microglia through endocytic or phagocytic pathways, it resides within membrane-bound endosomal/phagosomal compartments. Because selective autophagy receptors function on the cytosolic side of intracellular membranes, they would be unlikely to directly recognize luminal Aβ unless vesicular membrane breakdown exposes cargo to the cytosol or generates ubiquitinated compartmental membranes accessible to cytosolic receptors (*65*–*67*). Once Aβ-containing cargo or associated compartmental membranes become ubiquitinated and accessible to the cytosol, selective autophagy receptors such as p62/SQSTM1, optineurin (OPTN), and NBR1 can recognize ubiquitin tags and bridge cargo to LC3-positive autophagosomal membranes through LC3-interacting regions, promoting lysosomal delivery (*68*–*70*). In this context, we interpret SPP1-ItgaV signaling as enhancing the intracellular autophagic processing of Aβ-containing phagocytic/endocytic compartments rather than promoting direct receptor binding to extracellular Aβ. Phagocytosed or endocytosed cargo can engage noncanonical LC3-dependent pathways, including LC3-associated phagocytosis and LC3-associated endocytosis, in which LC3 is conjugated to single-membrane phagosomal or endosomal compartments to promote cargo maturation, trafficking, receptor recycling, and lysosomal degradation (*71*, *72*). In microglia, LC3-associated endocytosis has been shown to facilitate Aβ clearance and limit neurodegenerative pathology (*72*, *73*). Thus, SPP1-dependent upregulation of HTT/ALFY-associated receptor activity may facilitate the processing of LC3- and/or ubiquitin-marked Aβ-containing compartments after Aβ internalization. In Spp1 KO microglia, impaired activation of this pathway may leave Aβ-containing LC3-positive compartments stalled, reducing autolysosomal clearance and contributing to the accumulation of Aβ-associated cargo and a more toxic inflammatory state.

The complete loss of the selective autophagy receptors, especially Htt, tends to result in severe phenotypes (*74*–*78*). The phenotype in Spp1 KO mice is less severe. In young mice, global deletion of Spp1 does not cause observable neurological or visual deficits, though aging Spp1 KO mice lose retinal ganglion cells faster than wild-type mice (*23*, *79*). In the Spp1 KO mice, microglial expression of selective autophagy receptors was not completely abrogated. However, the upregulation of Htt, Wdfy3, and optineurin that usually occurs after injury to retinal ganglion cells was absent in Spp1 KO mice. This suggests that SPP1 may not play a significant role in the retina under physiological conditions but is mobilized explicitly as a defense against injury.

After an injury, SPP1 secretion from αRGCs may alert microglia to expect an increased need for debris removal. If this signal is missing, microglia fail to upregulate autophagy receptors and accumulate intracellular debris that is broken down only slowly. The cells respond with morphological signs of activation and a change in the gene expression profile. Aβ-treated microglia, especially those derived from Spp1 KO mice, inhibited ganglion cell growth and dendrite ramification in co-cultures of microglia and retinal ganglion cells. This suggests that under these conditions, microglia release soluble neurotoxic factors. A slow-release form of the SPP1 protein (SPP1 PODS) was associated with significantly reduced expression of several potential mediators of neurotoxicity in microglia containing Aβ aggregation, including IL-1β, IL-2, TNFα, IL-6, Ccl5, C3, and NOS.

Neuroprotective effects of SPP1 have been reported by several groups studying different conditions, including glaucoma, Alzheimer’s disease, and cerebral ischemia (*18*, *23*, *80*–*84*). However, it should be noted that the opposite outcome has also been observed (*85*–*88*). What may account for this discrepancy? Several studies have identified SPP1 as a biomarker because it is increased in the spinal fluid or the aqueous humor in the presence of Alzheimer’s disease or glaucoma, respectively (*89*–*92*). However, the elevation of a biomarker, useful though it is for diagnostic purposes, does not speak to the function of the biomarker molecule itself and should, therefore, not be interpreted as evidence for the detrimental role of SPP1 in these conditions. Other studies used knock-out mice or neutralizing antibodies to study the role of SPP1. Qiu and colleagues found that a small subset of disease-associated CD11c^+^/SPP1^+^ microglia in 5xFAD mice produces pro-inflammatory cytokines and does not efficiently clear Aβ. Genetic deletion or neutralizing antibodies improved cognitive function and neuronal survival in these mice (*86*). The main difference with our study is that, in contrast to brain microglia (*93*, *94*), we found no evidence that the retinal microglia themselves express SPP1, either before or after glaucomatous injury. Microglia are heterogeneous in terms of brain region, age, and the type and duration of a disease (*95*), and the most parsimonious explanation for the difference may be that CD11c^+^/SPP1^+^ disease-associated microglia are not a feature of glaucomatous neurodegeneration. Another recent study implicated SPP1 in microglia synapse engulfment in a murine model of Alzheimer’s disease (*88*). We did not observe a stimulatory effect of SPP1 on microglia phagocytosis; however, a limitation of our study is that we did not directly address microglial synapse pruning or count remaining RGC synapses in glaucomatous retinas from SPP1 KO animals. Given the heterogeneity of microglia in various disease stages and CNS regions, a careful analysis of microglia subtypes at different disease stages is necessary.

In human and macaque retinas, the number of SPP1^+^ ganglion cells is considerably larger than in the mouse. It is presently not known whether they all belong to the same type of retinal ganglion cells, but many of them are likely parasol cells, which share physiological similarities with the alpha cells observed in mice (*52*, *53*). In glaucomatous human and macaque eyes, retinal ganglion cell numbers were decreased compared to healthy controls; however, the cell survival rate was higher for SPP1^+^ expressing cells than SPP1^−^ ganglion cells. We found that the same may be true for tissue sections from two Alzheimer’s disease patients compared to the same number of controls. In both cases, SPP^+^ neurons appeared to be more resilient. However, due to the small number of samples from human retina and brain, these results are preliminary and require further study.

Spp1 itself has not been reported to be associated with human glaucoma in GWAS; however, one of its target genes, optineurin, has been linked to ALS and glaucoma (*96*, *97*). Glaucoma-relevant mutations of optineurin were shown to affect autophagy and lead to ganglion cell death even in the absence of elevated intraocular pressure (*98*, *99*). This suggests that SPP1 and its downstream cellular targets in ganglion cells and retinal microglia act independently of IOP. From a therapeutic standpoint, this is an advantage. All current drugs for glaucoma aim at lowering IOP, which makes SPP1 an attractive candidate for a combination therapy approach.

## MATERIALS AND METHODS

### Human samples

Human retina paraffin-embedded slides were obtained from the Lions Gift of Sight eye bank (University of Minnesota). Frontal cortex tissues from patients with Alzheimer’s disease (AD) and age-matched cognitively normal control donors were provided by the Massachusetts Alzheimer’s Disease Research Center Brain Bank. All AD cases met criteria for probable AD established by the National Institute of Neurological and Communicative Disorders and Stroke–Alzheimer’s Disease and Related Disorders Associations, as well as the National Institute on Aging–Reagan Institute criteria for high likelihood of AD. Retinal and cortical sections were processed for immunofluorescence following deparaffinization and antigen retrieval. Briefly, tissues were deparaffinized in xylene, rehydrated through a graded ethanol series, and subjected to antigen retrieval in Tris-EDTA buffer (10 mM Tris, 1 mM EDTA, 0.05% Tween 20, pH 9.0) for 20 min at 95°C, followed by cooling to room temperature. Sections were blocked in 5% bovine serum albumin in PBS for 1 hour at room temperature and then incubated with the primary antibodies overnight at 4°C. The following primary antibodies were used: anti-SPP1 (R&D Systems, AF1433), anti-ItgαV (Thermo Fisher Scientific, 14–0512-82), anti-GFAP (Abcam, ab4674), anti-NeuN (Cell Signaling Technology, 24307), anti-IBA1 (Abcam, ab5076), anti-p62 (Millipore, P0067), and Alexa Fluor 488–conjugated anti-β-Amyloid (BioLegend, 803013) (table S1). Donkey anti-goat IgG (Alexa Fluor 594), donkey anti-rat IgG (Alexa Fluor 488), donkey anti-chicken IgG (Alexa Fluor 488), donkey anti-rabbit IgG (Alexa Fluor 488), and donkey anti-rabbit IgG (Alexa Fluor 594) were used for the detection of SPP1, ItgαV, GFAP, NeuN, p62, and IBA1, respectively. Nuclei were counterstained with DAPI, and slides were mounted using VECTASHIELD Antifade Mounting Medium (Vector Laboratories, H-1000). Confocal images were acquired using a Leica SP8 microscope (Leica Microsystems, Wetzlar, Germany). 3D reconstruction of neurons, microglia, and intracellular proteins were performed by Imaris software.

### Nonhuman primate samples

Nonhuman primate (NHP) retina paraffin-embedded slides were kindly provided by Priya Chaudhary and Claude Burgoyne (Devers Eye Institute). The NHP retina tissues were collected from NHP experimental glaucoma models that used serial laser treatment to the trabecular meshwork of one eye of NHP rhesus macaques (*Macaca mulatta*) to induce IOP elevation, as described in detail (*100*, *101*). Tissues were deparaffinized with xylene and rehydrated with a decreasing ethanol gradient. Tissues were blocked in 5% bovine serum albumin (BSA) in PBS for 1 hour and incubated with the anti-SPP1 antibody (MyBioSource, MBS4506382) solution at 4°C for 24 hours. A donkey anti-goat IgG (Alexa Fluor 594) was used for SPP1 detection, and nuclei were counterstained with DAPI. Slides were imaged using a Leica SP8 confocal microscope (Leica, Wetzlar, Germany).

### Animals

Wild-type C57BL/6 J, Spp1 KO (B6.129S6(Cg)-Spp1^tm1Blh^/J), Ai14 (B6.Cg-Gt(ROSA)26Sor^tm14(CAGtdTomato)Hze^/J), Atg7-flox (C57BL/6 J-*Atg7^em1Lutzy^*/J), Cx3cr1-cre (B6J.B6N(Cg)-*Cx3cr1^tm1.1(cre)Jung^*/J), CX3CR1-GFP (B6.129P2(Cg)-*Cx3cr1^tm1Litt^*/J), Kcng4-cre (B6.129(SJL)-*Kcng4^tm1.1(cre)Jrs^*/J), Gfap-cre (B6.Cg-Tg(Gfap-cre)77.6Mvs/2 J), and Thy1-YFP (Tg(Thy1-cre/ERT2,-EYFP)HGfng/PyngJ) mice were purchased from The Jackson Laboratory. The strain, Spp1^GFPfl/fl^ (B6.Spp1^fl-EGFP-stop-tdTomato^) for Spp1 conditional deletion, was generated by Cyagen (Santa Clara, California) and was characterized in detail in a previous publication (*23*). The strain, ItgαV-flox (B6.ItgαV^flox/flox^), was a gift from Adam Lacy-Hulbert (Benaroya Research Institute, Seattle, Washington) (*102*). Age- and strain-matched mice were used for the study. Male and female mice were used in equal numbers. Animals received an ear tag for later identification and were randomized into experimental groups. The experimenter applying the ear tag and the experimenter conducting the experiment were different. From our previous experience with typical inter-group differences and standard deviations, 5 animals per group would provide 90% power to detect differences at α = 0.01 in pERG (the test with the highest variability). To maintain a balanced 1:1 male-to-female ratio and account for potential attrition, we used at least 6 mice per group. The experimental designs followed the ARRIVE Essential 10. All animals were housed under standard conditions (12-hour light/dark cycle) and received water and standard food *ad libitum*. At the end of the experiments, mice were euthanized by CO_2_ inhalation in compliance with the Guide for the Care and Use of Laboratory Animals of the AAALAC. All animal procedures were conducted following the guidelines of the Association for Research in Vision and Ophthalmology and were approved by the Institutional Animal Care and Use Committee at Schepens Eye Research Institute (animal protocol approval number 2021 N000075).

### Generation of experimental mouse lines

#### Spp1 cKO

Heterozygous transgenic Kcng4-cre mice were crossed with homozygous Spp1^GFPfl/fl^ (Spp1-flox) mice and further bred to generate offspring with homozygosity for the Spp1-flox allele to generate Kcng4-cre; Spp1-flox (Spp1^GFPfl/fl^Kcng4Cre) mice. Cre-negative Spp1^GFPfl/fl^ littermates were used as controls.

#### Kcng4-cre; Ai14

Heterozygous transgenic Kcng4-cre mice were crossed with homozygous reporter strain Ai14 to produce Kcng4-cre; Ai14 (Kcng4-tdTomato) mice.

#### Kcng4-cre; Ai14; Thy1-YFP

Mice were generated by breeding Kcng4-tdTomato with Thy1-YFP mice, producing Kcng4-cre; Ai14; Thy1-YFP.

#### GFAP-cre; Ai14

Heterozygous transgenic GFAP-cre mice were crossed with homozygous reporter strain Ai14 to produce GFAP-cre; Ai14 mice.

#### ItgαV cKO

Heterozygous transgenic Cx3cr1-cre mice were bred with homozygous ItgαV-flox mice and further bred to generate offspring with homozygosity for the ItgαV-flox allele to generate Cx3cr1-cre; ItgαV-flox mice. Cre-negative ItgαV-flox littermates were used as controls.

#### Spp1 KO; CX3CR1-GFP

Heterozygous transgenic CX3CR1-GFP mice were crossed with homozygous Spp1 KO mice and further bred to generate homozygosity for mutant Spp1 allele to produce Spp1 KO; CX3CR1-GFP mice.

#### Spp1 cKO; CX3CR1-GFP

Heterozygous transgenic CX3CR1-GFP mice were crossed with homozygous Spp1^GFPfl/fl^ (Spp1-flox) and further bred to generate homozygosity for the Spp1-flox allele to generate CX3CR1-GFP; Spp1-flox mice. CX3CR1-GFP; Spp1-flox mice were further bred with Spp1 cKO (Kcng4-cre; Spp1-flox) mice to generate offspring with homozygosity for the Spp1-flox allele to generate Kcng4-cre; Spp1-flox; CX3CR1-GFP mice.

#### Kcng4-tdTomato; CX3CR1-GFP

Heterozygous transgenic CX3CR1-GFP mice were crossed with Kcng4-tdTomato mice to generate Kcng4-cre; Ai14; CX3CR1-GFP mice.

#### Atg7^cKO^

Heterozygous transgenic Cx3cr1-cre mice were bred with homozygous Atg7-flox mice and further bred to generate offspring with homozygosity for the Atg7-flox allele to generate Cx3cr1-cre; Atg7-flox mice. Cre-negative Atg7-flox littermates were used as controls.

### Primary microglia culture

Primary cultured microglia were obtained from the mixed glial culture from the retinas of neonatal pups of 1–3 days old, as described previously (*35*). Briefly, retinas were isolated from either CX3CR1-GFP or Spp1 KO; CX3CR1-GFP pups and dissociated into single-cell suspensions using 0.25% trypsin and 0.01% DNase in DMEM. Cell suspensions were then filtered through 52-μm nylon mesh filters, centrifuged, resuspended, and plated on polylysine-coated cell culture plates in DMEM/F12 with 10% FBS for 2 weeks. Floating microglia were detached from the astrocyte layer by shaking at 200 rpm and collected from the plates. Microglia were filtered through the 52-μm nylon mesh filters to remove cell debris and then plated onto polylysine-coated culture dishes or coverslips in DMEM/F12 with 10% FBS 5% CO_2_ at 37°C incubator. Cell culture medium was changed every 3–4 days.

### Primary cell co-culture of RGCs and microglia

Primary cultured RGCs were isolated from P1-P3 C57BL/6 pups, as described previously (*23*). Retinas were isolated and digested in pre-warmed papain solution (20 U/ml) containing DNase I (100 U/ml) at 37°C for 15 min. The reaction was stopped by adding an equal volume of ovomucoid (5 mg/ml). Lysates were triturated and centrifuged at 3000 rpm for 5 min at 4°C, followed by resuspension in 500 μl washing buffer (0.5% BSA, 2 mM EDTA in PBS). RGCs were isolated from the cell suspension using micro-magnetic beads conjugated with the Thy1.2 (CD90.2) antibody (Invitrogen, 11443D) via a MACS magnetic separation system (Miltenyi Biotec, 130–090-312), following the manufacturer’s instructions. RGCs were centrifuged at 3000 rpm for 5 minutes at 4°C, then resuspended in DMEM/F12 medium containing 10% FBS, 10 mM HEPES, 2 mM L-glutamine, and 100 U/ml Penicillin-Streptomycin. Purified and resuspended RGCs were plated on polylysine-coated coverslips. Three different microglia-neuron co-cultures were performed as described below. (1) For conditioned medium co-culture, primary cultured WT GFP^+^ microglia were treated with 0.5 μM Aβ protein (Aβ_1–42_-555) or PBS for 48 hours. Conditioned cell medium was collected from cultured microglia and transferred to primary cultured RGCs for 48 hours. (2) For microglia-neuron co-culture without physical contact, primary cultured WT GFP^+^ microglia were seeded on coverslip-1 and treated with 0.5 μM Aβ protein or PBS for 48 hours. Coverslip-1 with microglia and coverslip-2 with RGC growth were transferred to a bigger culture dish and co-cultured in fresh DMEM/F12 with 10% FBS medium for 48 hours. (3) For microglia-neuron co-culture with physical contact, primary cultured WT and Spp1 KO GFP^+^ microglia were treated with 0.5 μM Aβ protein or PBS for 48 hours. Cultured microglia were washed, resuspended, and transferred to RGC coverslips and co-cultured in fresh DMEM/F12 with 10% FBS medium for 48 hours. At the end of the co-cultures, RGCs were washed and fixed with 4% paraformaldehyde and then stained with β3-tubulin (Sigma-Aldrich, T2200). Images were taken using an SP8 confocal microscope, and a 3D reconstruction of RGC neurites was performed with Imaris software.

### Human iPSC-derived microglia

Human iPSC-derived microglia were obtained from Elixirgen Scientific (Quick-Glia Microglia, MG-SeV-CW50065) and cultured according to the manufacturer’s protocol. Tissue culture plates were coated with 10 μg/ml fibronectin (Corning, 354008) for 3 hours at room temperature. ScienCell Medium (ScienCell Research Laboratories, 1901) was prepared by supplementing Basal Medium with 5% fetal bovine serum, 1% Microglia Growth Supplement, and 1% penicillin–streptomycin. Microglia Medium was then prepared by combining ScienCell Medium with proprietary Components MG1, MG2, and MG3 and equilibrated to room temperature before use. Cryopreserved cells (>1 × 10^6^ viable cells per vial) were rapidly thawed in a 37°C water bath, gently diluted with ScienCell Medium, and centrifuged at 200 g for 4 min. The supernatant was removed, leaving ∼50 μl of medium to cover the pellet, which was gently resuspended in Microglia Medium MG3. Cells were counted and plated at 1 × 10^5^ cells/cm^2^ and evenly distributed by gentle movement. Cultures were maintained at 37°C in a humidified 5% CO_2_ incubator, with a complete Microglia Medium change on day 2 and half-medium changes every 2–3 days thereafter. By day 7, cells exhibited a characteristic ramified microglial morphology and expressed the canonical microglial markers IBA1 and TMEM119. Differentiated microglia were used for downstream assays or maintained in Microglia Medium for extended culture.

### Human iPSC-derived neurons

Human iPSC-derived neurons were obtained from Elixirgen Scientific (Quick-Neuron, EX-SeV-CW50065) and cultured according to the manufacturer’s instructions. Tissue culture plates were sequentially coated with 0.002% poly-L-ornithine (Sigma-Aldrich, P4957) and 1% Geltrex Basement Membrane Matrix (Thermo Fisher Scientific, A4000046801) at 37°C for 2 hours. Medium N was prepared using DMEM/F12 (Thermo Fisher Scientific, 21331020), Neurobasal medium (Thermo Fisher Scientific, 21103049), GlutaMAX (Thermo Fisher Scientific, 35050061), penicillin–streptomycin (Thermo Fisher Scientific, 15140122), and Elixirgen Component N. Medium N(G2P) was formulated by adding Components G2 and P (1:1000 and 1:2000, respectively) to Medium N and equilibrated to room temperature before use. Cryopreserved neurons (>1 × 10^6^ viable cells per vial) were rapidly thawed in a 37°C water bath, gently diluted with Medium N, centrifuged at 200 g for 4 min, and resuspended in Medium iN(G2P) containing 10 μM ROCK inhibitor Y-27632 (MedChemExpress, HY-10583). Cells were plated at a density of 5 × 10^4^ cells/cm^2^ on coated plates and cultured at 37°C in a humidified 5% CO_2_ incubator. On day 2, medium was fully replaced with fresh Medium N(G2P), followed by half-medium changes every 2–3 days. By day 7, neurons exhibited typical neurite outgrowth and expressed the pan-neuronal markers β3-tubulin and MAP2. Differentiated neurons were used for downstream assays or maintained in Maintenance Medium for extended culture.

### Co-cultures of human iPSC-derived microglia and neurons

Human iPSC-derived microglia and neurons were cultured separately as described above. Microglia were seeded on poly-L-lysine–coated coverslips (coverslip-1), and neurons were plated on poly-L-ornithine/laminin–coated coverslips (coverslip-2) and differentiated for 7 days in their respective medium. On day 7, mature microglia were treated with vehicle (PBS), 0.5 μM Aβ peptide, or 0.5 μM Aβ peptide combined with 50 ng/ml SPP1 PODS for 24 or 48 hours. Following treatment, coverslip-1 containing microglia and coverslip-2 containing neurons were transferred to a shared culture dish and co-cultured for 48 h in a 1:1 mixture of Neurobasal and Microglia Medium supplemented with all necessary growth components for both cell types. After co-culture, neuronal coverslips were washed with PBS, fixed with 4% paraformaldehyde, and immunostained for β3-tubulin. Confocal images were acquired using a Leica SP8 microscope, and neurite architecture was analyzed by 3D reconstruction using Imaris software.

### Fundus photography

Mice were anesthetized with an intraperitoneal injection of ketamine (100 mg/kg) and xylazine (20 mg/kg), followed by pupil dilation using 1% tropicamide. GenTeal gel eye drops (Alcon, Fort Worth, TX) were applied to maintain corneal moisture and clarity. Fundus images were obtained with a Micron III retinal imaging system (Phoenix Research Laboratories, Pleasanton, CA).

### Optical coherence tomography

Mice were anesthetized with ketamine (100 mg/kg) and xylazine (20 mg/kg), pupils were dilated with 1% tropicamide, and corneas were treated with GenTeal gel eye drops. Optical coherence tomography (OCT) images of the anterior and posterior segments were acquired with a spectral domain OCT system (Bioptigen, Morrisville, NC). Averaged single B-scans and volume scans were obtained, with imaging centered on the optic nerve head or the cornea.

### Electroretinography

Pattern electroretinograms (pERG) were recorded from mouse eyes using a Celeris small animal testing system (Diagnosys LLC) (*23*, *24*). Mice were anesthetized with ketamine/xylazine, and the pupils were dilated with one drop of 1% tropicamide. Artificial tears (GenTeal) were applied to the corneas to prevent drying. The mice were placed on a built-in warm plate to maintain their body temperature at 37°C. PERG was measured using a high-contrast horizontal grating (0.05 cycles/degree), with spatial phase reversal at 1 Hz and a mean luminance of 50 cd/m^2^. An electrode placed on the contralateral eye was used as a reference. Each eye underwent two repetitions of 300 complete contrast reversals during pERG testing. PERG amplitudes were measured as the difference between the P1 peak and N2. A conventional, light-adapted ERG was recorded directly afterward to ensure that reductions in PERG amplitudes were not due to unrelated ocular disease.

### Optomotor response

Visual acuity was assessed in awake mice using an OptoDrum device (Striatech GmbH, Germany). Mice were placed on a 2-inch-diameter platform located in the center of an arena surrounded by four computer monitors. A camera positioned above the arena recorded the mice’s behavior. Vertical black-and-white stripe pattern rotated clockwise or counterclockwise on the screens to stimulate the optomotor reflex on the left or right eye, respectively. The rotation speed (12°/s) and contrast (100%) remained constant. Head or body movements in the direction of the rotating stimulus were identified as positive tracking responses, which were automatically detected and analyzed by OptoDrum software. The stimulus pattern frequency was continuously and automatically adjusted until it reached the visual threshold. The highest frequency that still elicited a tracking response was recorded as the visual acuity threshold.

### Optic nerve crush

Mice were anesthetized, and the intraorbital optic nerve was exposed by dissection of the conjunctiva as previously described (*23*, *103*). The Dumont #5/45 Forceps (Fine Science Tools, Foster City, California) was used to clamp the exposed optic nerve for 10 seconds at about 1 mm distal to the lamina cribrosa. Only one optic nerve was crushed, and the other eye was not crushed. After the procedure, triple antibiotic ointment was applied to the injured eye, and mice received a subcutaneous injection of Ethiqa XR (3.5 mg/kg) for pain relief. At the end of the experiments, retinas were collected for cell sorting and immunohistochemistry.

### Microbead occlusion model

Chronic ocular hypertension was induced using the microbead occlusion model as previously described (*104*, *105*). Mice were anesthetized, and a small hole was made in the cornea of the right eye with a 30.5-gauge needle. Polystyrene microbeads (15 μm, FluoSpheres, Invitrogen) were suspended in sterile normal saline and injected into the anterior chamber using a beveled glass micropipette connected to a Hamilton syringe. Control mice received an injection of sterile normal saline. Intraocular pressure (IOP) was measured every 3 days for 4 weeks using a TonoLab rebound tonometer (ICare, Espoo, Finland) under 1.5% isoflurane anesthesia. IOP tests were performed at the same time each morning to minimize circadian variability. Each IOP was averaged from five consecutive measurements per eye. Signs of anterior chamber or corneal inflammation as a complication of the surgery or a failure to develop an IOP elevation were exclusion criteria for this study, however, no complications were observed, and no animals were actually excluded.

### Fluorescence-activated cell sorting

Retinal microglia were isolated from CX3CR1-GFP, Spp1 KO; CX3CR1-GFP, ItgαV, and ItgαV cKO as previously described (*23*). To obtain sufficient microglia for FACS-based analyses, retinas from multiple mice of the same genotype and condition were pooled before dissociation. Because cells were pooled before sorting, individual sorted microglia could not be assigned to a specific mouse. For single-cell analyses, we therefore report the number of microglia analyzed. Retinas were digested in Ca2^+^ and Mg^2+^-free HBSS (Gibco, 14185–052) containing 0.6 mg/ml papain (Worthington, LS003126), 0.012 mg/ml L-cysteine (Sigma, C7352) for 15 min at 37°C. Tissues were then centrifuged, papain/HBSS solution was discarded, and tissues were resuspended in HBSS containing 10% horse serum (Gibco, 26050–088) and 60 U/ml DNase I (Sigma, D-5025). Tissue dissociation was performed by gentle trituration with a heat-polished Pasteur pipette (World Precision Instruments, TW150–4) until no visible tissue chunks remained. The dissociated cells were centrifuged and subsequently resuspended in HBSS. Cell suspensions were filtered through a 35-μm cell strainer into 5 ml round-bottom polystyrene test tubes (Falcon, 352235). For cell sorting of GFP^+^ microglia from CX3CR1-GFP and Spp1 KO; CX3CR1-GFP mice, single-cell suspensions were sorted on a FACS Aria III cell sorter (BD Biosciences) to collect the live, singlet GFP^+^ cells as the microglia. To sort retinal microglia from ItgαV cKO mice, single-cell suspensions were incubated with 5 μg/ml PE-Cyanine7-anti-CD11b (eBioscience, 25–0112-82) and 5 μg/ml Alexa 488–anti-CD45 (BD Biosciences, 567377) antibodies for 30 min on ice. Cells were rinsed and centrifuged at 500 g for 5 min, resuspended in the IsoFlow Sheath Fluid (Beckman Coulter, 8547008), and sorted by CD11b and CD45 expression using a FACS Aria III cell sorter (BD Biosciences). Live, single CD11b^+^CD45^low^ cells were identified as microglia. FACS-sorted microglia were centrifuged, and the cell pellets were used for further RNA extraction and immunostaining.

### Quantitative PCR (qPCR)

RNA extraction and purification from primary cultured microglia and sorted microglial cell pellets were performed as previously described (*23*). Total RNA was extracted from microglia using the RNeasy Plus Micro Kit (Qiagen, 74034) according to the manufacturer’s instructions. The RNA purity was tested on the NanoDrop 2000 (Thermo Fisher Scientific). RNA integrity and concentration were assessed with Agilent RNA 6000 Pico kit (Agilent Technologies, 5067–1513) on the Agilent 2100 BioAnalyzer (Agilent Technologies, Santa Clara, CA). Only RNA samples with a 260/280 ratio above 1.8 and RIN greater than 5 were used for cDNA synthesis. cDNA was synthesized from the total RNA with High-Capacity cDNA Reverse Transcription Kit (Applied Biosystems, 4368814). The real-time PCRs were performed using SYBR Green PCR Master Mix (Applied Biosystems, 4309155) on the StepOnePlus qPCR thermocycler (Applied Biosystems, Foster City, CA) and using FastStart Essential DNA Green Master (Roche Diagnostics, 06402712001) on the Roche LightCycler 480 II (Roche Diagnostics, Indianapolis, IN). Primer sequences (Eurofins Genomics) are given in table S2. GAPDH was used as a reference gene.

### RNA sequencing

Total RNA was extracted from FACS-sorted microglia pellets from CX3CR1-GFP and Spp1 KO; CX3CR1-GFP mice using the RNeasy Plus Micro Kit (Qiagen, 74034). RNA purity, integrity, and concentration were assessed using the NanoPhotometer (IMPLEN), Agilent 2100 BioAnalyzer with Agilent RNA 6000 Pico kit (Agilent Technologies, 5067–1513), and Qubit 2.0 Fluorometer (Life Technologies) with Qubit RNA Assay Kit. cDNA libraries were prepared and sequenced on an Illumina NovaSeq 6000 instrument at Novogene (Sacramento, CA), using paired-end 150-bp sequencing.

### RNA-seq analysis

The RNA-seq raw read data were deposited in NCBI-GEO with accession number GSE304931. The alignment program salmon was used to map reads (fastqs) to the mouse reference genome (GRCm38) on the computer cluster of the Harvard Chan Bioinformatics Core (HBC). The R package DESeq2 was used to analyze differential gene expression with false-discovery-rate-adjusted *P* < 0.05 and absolute log2 (fold change) > 0.58. Gene expression was defined as upregulated when log2FC > 0.58 or downregulated when log2FC < −0.58, with FDR < 0.05. An additional analysis was performed with the Python package PyDESeq2 using the same criteria for differential expression. Both scripts are available in the Dataverse associated with this manuscript (https://doi.org/10.7910/DVN/WJ0MJI). Functional enrichment was analyzed using Ingenuity Pathway Analysis (Qiagen).

### Immunohistochemistry

Mouse eyes were obtained and fixed in 4% paraformaldehyde for 1 h. Retinas were dissected, placed ganglion cell side-up on nitrocellulose membranes, and stained with primary antibodies (see key resource table) at 4°C for 3 days (*106*). Some fixed retinas were embedded in OCT and sectioned at 12 μm in a Leica cryostat. Retinal sections were incubated with blocking/permeabilization buffer (5% donkey serum and 0.3% Triton X-100 in PBS) for 30 min at room temperature, followed by staining with primary antibodies for 1-3 hours at room temperature. Secondary antibodies conjugated with Alexa fluorophores (Jackson ImmunoResearch and Molecular Probes) were used to detect primary antibodies. Nuclei were counterstained with DAPI. Confocal images were captured using a SP8 confocal microscope (Leica, Wetzlar, Germany). Image analysis and quantification were performed with LAS X, Fiji, Matlab, and Imaris software.

### Immunocytochemistry

For primary cultured microglia and RGCs, cells on coverslips were fixed in 4% paraformaldehyde for 10 min at room temperature, incubated with blocking/permeabilization buffer (5% donkey serum and 0.3% Triton X-100 in PBS) for 30 min at room temperature, and stained with primary antibodies (see key resource table) for 1-3 hours at room temperature followed by secondary antibodies conjugated with Alexa fluorophores. For FACS-sorted single microglia, cells were plated on Cell Culture Inserts membranes (Millipore), centrifuged, and fixed in 4% paraformaldehyde for 10 min at room temperature. Cells were incubated with blocking/permeabilization buffer (5% donkey serum and 0.3% Triton X-100 in PBS) for 30 min at room temperature, then stained with primary antibodies overnight at 4°C, followed by staining with Alexa fluorophore-conjugated secondary antibodies. Nuclei were counterstained with DAPI. Fluorescent images were obtained with a SP8 confocal microscope (Leica, Wetzlar, Germany). Image analysis was performed with LAS X, Fiji, Matlab, and Imaris software.

### Proteome profiler array

Relative cytokine levels from cultured microglia were analyzed using the Proteome Profiler Mouse Cytokine Array Kit, Panel A (R&D Systems, ARY006) according to the manufacturer’s instructions (*36*, *37*). Primary microglial cultures were treated for 24 h under three conditions: wild-type (WT) microglia treated with 0.5 μM amyloid-β (Aβ), *Spp1* knockout (KO) microglia treated with 0.5 μM Aβ, and *Spp1* KO microglia treated with 0.5 μM Aβ in the presence of SPP1 PODS (50 ng/mL). Following treatment, cells were lysed in RIPA buffer (Sigma-Aldrich, R0278), and total protein concentrations were determined using the Micro BCA Protein Assay Kit (Thermo Scientific, 23235). Equal amounts of protein (200 μg per sample) were prepared in Array Buffer 4 and adjusted to a final volume of 1.5 ml with Array Buffer 6. Samples were then incubated with 15 μl of reconstituted Detection Antibody Cocktail for 1 hour at room temperature. The sample-antibody mixtures were applied to nitrocellulose membranes, previously blocked with Array Buffer 6, and incubated overnight at 4°C on a rocking platform shaker. After washing three times with 1X Wash Buffer, membranes were incubated with Streptavidin-HRP for 30 min. Following a final wash cycle, chemiluminescent detection was performed using the Chemi Reagent Mix. Chemiluminescent signals from three membranes were captured simultaneously at a constant exposure time using an iBright 1500 Imaging System (Invitrogen). Pixel densities of duplicate spots were quantified in Fiji (ImageJ) for each membrane to determine relative cytokine levels across experimental groups.

### Microglial phagocytosis assay

For measurement of microglial phagocytic activity, primary cultured GFP^+^ microglia were grown on polylysine-coated coverslips in 24-well plates. The pHrodo Red *E. coli* BioParticles Conjugate (Thermo Fisher Scientific, P35361) was resuspended in live imaging solution at 1 mg/ml. At an appropriate density, microglia were incubated with pHrodo Red *E. coli* BioParticles Conjugate (0.1 mg/ml) in DMEM/F12 complete medium for 15 min, 30 min, 1 hour, 2 hours, and 4 hours. After removing the cell culture medium and PBS washing, the microglia were fixed with 4% paraformaldehyde and imaged using a SP8 confocal microscope. 3D reconstruction of microglia was performed with Imaris software. Phagocytic activity was determined based on the percentage of bioparticles in microglia.

### Autophagic flux assay

Autophagic flux was assessed using the Autophagic Flux Assay Kit (Dojindo, A562) according to the manufacturer’s instructions. DALGreen and DAPRed were reconstituted in DMSO to generate stock solutions (1 mmol/liter and 0.1 mmol/liter, respectively); Bafilomycin A1 (Baf. A1) was similarly dissolved in DMSO and stored at −20°C, protected from light. Working solutions were freshly prepared on the day of the experiment by diluting DALGreen and DAPRed stocks in culture medium to final concentrations of 1.0 μmol/liter and 0.2 μmol/liter, respectively. Cells cultured in 24-well plates were washed twice with PBS and incubated with 200 μl of DALGreen/DAPRed working solution for 30 min at 37°C in a humidified 5% CO_2_ incubator. Following staining, cells were washed twice with PBS and subjected to three experimental conditions: (i) a control group incubated in complete medium for 2 hours 20 min; (ii) a starvation group incubated in amino acid-free medium (Fujifilm, 048–33575) for 2 hours 20 min to induce autophagy; and (iii) a lysosomal inhibition group, in which cells were incubated in amino acid-free medium for 2 hours followed by the addition of Baf. A1 (1:2000 dilution) for the final 20 min to block autophagosome-lysosome fusion and acidification. Fluorescence signals were acquired using a Leica SP8 confocal microscope. To prevent spectral bleed-through between overlapping fluorophores, images were captured in sequential scan mode between frames with the following settings: DALGreen (λex 405 nm, λem 500–550 nm), GFP (λex 488 nm, λem 500–550 nm), and DAPRed (λex 561 nm, λem 565–700 nm). Autophagic flux was quantified by calculating the change in DAPRed intensity (∆DAPRed) between starvation and starvation plus Baf. A1 conditions, representing the net accumulation of autophagic vacuoles.

### AAV2-mediated gene transfer

The Spp1 coding region, followed by a cleavable F2A linker and the EGFP gene, was synthesized by Integrated DNA Technologies (Coralville, Iowa). The synthesized SPP1-F2A-EGFP gene was then inserted into the AAV2 backbone plasmid, sequenced, and used to produce virus (1.4 × 10^12^ GC/ml, Viral Vector Core, MEEI). An AAV2 construct expressing only EGFP was used as a control. Both constructs used the CMV promoter for gene expression. The mice received an intravitreal injection of 1 μL AAV2-Spp1-EGFP or control virus AAV2-EGFP. The transfection efficiency of AAV2 in retinas was assessed by fundus photography and immunohistochemistry.

### SPP1 PODS treatment in vivo and in vitro

One microliter of 500 ng/ml slow-release SPP1 protein (SPP1 PODS) (Cell Guidance Systems, PPH319–250) was intravitreally injected into the eyes of mice, followed by microbead injection or optic nerve crush. Mice received SPP1 PODS weekly and were anesthetized to harvest the retinas at the end of the in vivo experiments. In vitro, 50 ng/ml SPP1 PODS were administered to primary cultured microglia and human iPSC-derived microglia and incubated for 24 or 48 hours. PBS served as the control.

### siRNA knockdown

Htt siRNA, Wdfy3 siRNA, Optn siRNA, Ubqln2 siRNA, CD44 siRNA, ItgαV siRNA, and negative control siRNA were purchased from Thermo Fisher Scientific. Primary cultured microglia were transfected with siRNA (50 nM) using Lipofectamine 3000 transfection reagent (Invitrogen, L3000001) according to the manufacturer’s instructions. Microglia were cultured in DMEM/F-12 complete media with siRNA and incubated for 24–48 hours post-treatment. The knockdown efficiency was first validated by qPCR.

### Live-cell time-lapse imaging

Primary cultured WT and Spp1 KO GFP^+^ microglia were isolated from retinas of CX3CR1-GFP and Spp1 KO; CX3CR1-GFP pups (P1-P3) and cultured in DMEM/F12 with 10% FBS in 12-well plates. After PBS washing several times, the primary cultured microglia were treated with 0.5 μM Aβ protein in fresh DMEM/F-12 without phenol red (Gibco, 11039021). PBS served as the control treatment. To test the effects of SPP1 PODS on microglial debris clearance, microglia were treated with 50 ng/ml SPP1 PODS, followed by treatment with 0.5 μM Aβ protein. The 12-well plates were transferred to a Leica DMi8 microscope to perform live-cell time-lapse imaging (*107*, *108*). In each well, 8–10 fields were selected and scanned at 10-minute intervals for 24 hours. Microglial debris clearance was analyzed by Aβ degradation latency, which was the time it took for the aggregate to disappear completely in live cultured microglia.

### 3D reconstruction of confocal images

Z-stack confocal images were obtained from retinas, primary cultured microglia, and primary cultured RGCs using a SP8 confocal microscope. 3D reconstruction of confocal images was performed remotely using Imaris software (Oxford Instruments, Abingdon, United Kingdom) on the computer of the Image Analysis Collaboratory at Harvard Medical School. 3D reconstruction of morphology and subcellular localization was processed using the ‘cell’, ‘filament’, ‘surface’, and ‘mask’ plug-ins in Imaris software. Images were analyzed and quantified by Imaris. For retinal microglia and cultured microglia, microglial volume was measured. For intracellular compartments, including apoptotic RGC debris and aggregated proteins (Aβ40, Aβ42, APP, α-synuclein, and synapsin), the numbers and volumes were measured. Aβ total volume per cell and Aβ aggregation percentage were also assessed. For RGC morphological analysis, the RGC neurite length, neurite volume, branch point, and terminal points were tested. For LC3B^+^ and P62^+^ puncta, the number, volume, total volume in microglia, and volume percentage were analyzed.

### Statistical analysis

Statistical data analysis was performed using GraphPad Prism 10. The data were provided as mean ± SEM, unless otherwise indicated. Box-and-whiskers plots indicate median, the 25th and 75th percentiles (box), and minimum and maximum values (whiskers). For comparisons between two groups, data were analyzed using an unpaired two-tailed t-test. For comparison between multiple groups, normally distributed data were analyzed using a one-way ANOVA with Tukey’s post-test. For two-factor comparisons, a two-way ANOVA with Bonferroni’s post-test was used. A *P*-value less than 0.05 was considered significant. *P < 0.05, **P < 0.01, and ***P < 0.001. Differential gene expression (DGE) analysis was done using R and RStudio and Python. Figures were prepared using Adobe Photoshop 2025. Schematic images were prepared using BioRender with publication licenses.

## References

[1] R. M. Ransohoff, D. Schafer, A. Vincent, N. E. Blachere, A. Bar-Or, Neuroinflammation: Ways in which the immune system affects the brain. Neurotherapeutics 12, 896–909 (2015). 26306439 10.1007/s13311-015-0385-3 PMC4604183

[2] M. Noda, Y. Doi, J. Liang, J. Kawanokuchi, Y. Sonobe, H. Takeuchi, T. Mizuno, A. Suzumura, Fractalkine attenuates excito-neurotoxicity via microglial clearance of damaged neurons and antioxidant enzyme heme oxygenase-1 expression. J. Biol. Chem. 286, 2308–2319 (2011). 21071446 10.1074/jbc.M110.169839 PMC3023525

[3] M. Noda, K. Takii, B. Parajuli, J. Kawanokuchi, Y. Sonobe, H. Takeuchi, T. Mizuno, A. Suzumura, FGF-2 released from degenerating neurons exerts microglial-induced neuroprotection via FGFR3-ERK signaling pathway. J. Neuroinflammation 11, 76 (2014). 24735639 10.1186/1742-2094-11-76 PMC4022102

[4] C. Xing, E. H. Lo, Help-me signaling: Non-cell autonomous mechanisms of neuroprotection and neurorecovery. Prog. Neurobiol. 152, 181–199 (2017). 27079786 10.1016/j.pneurobio.2016.04.004 PMC5064822

[5] A. Suzumura, Neuron-microglia interaction in neuroinflammation. Curr. Protein Pept. Sci. 14, 16–20 (2013). 23544747 10.2174/1389203711314010004

[6] J. E. Dowling, *The Retina: An approachable part of the brain* (Harvard Univ. Press, 2012).

[7] R. H. Masland, The neuronal organization of the retina. Neuron 76, 266–280 (2012). 23083731 10.1016/j.neuron.2012.10.002 PMC3714606

[8] H. A. Quigley, Glaucoma. Lancet 377, 1367–1377 (2011). 21453963 10.1016/S0140-6736(10)61423-7

[9] R. H. Masland, The fundamental plan of the retina. Nat. Neurosci. 4, 877–886 (2001). 11528418 10.1038/nn0901-877

[10] J. R. Sanes, R. H. Masland, The types of retinal ganglion cells: Current status and implications for neuronal classification. Annu. Rev. Neurosci. 38, 221–246 (2015). 25897874 10.1146/annurev-neuro-071714-034120

[11] B. A. Rheaume, A. Jereen, M. Bolisetty, M. S. Sajid, Y. Yang, K. Renna, L. Sun, P. Robson, E. F. Trakhtenberg, Single cell transcriptome profiling of retinal ganglion cells identifies cellular subtypes. Nat. Commun. 9, 2759 (2018). 30018341 10.1038/s41467-018-05134-3 PMC6050223

[12] Q. Cui, C. Ren, P. J. Sollars, G. E. Pickard, K. F. So, The injury resistant ability of melanopsin-expressing intrinsically photosensitive retinal ganglion cells. Neuroscience 284, 845–853 (2015). 25446359 10.1016/j.neuroscience.2014.11.002 PMC4637166

[13] Y. Ou, R. E. Jo, E. M. Ullian, R. O. Wong, L. Della Santina, Selective vulnerability of specific retinal ganglion cell types and synapses after transient ocular hypertension. J. Neurosci. 36, 9240–9252 (2016). 27581463 10.1523/JNEUROSCI.0940-16.2016 PMC5005727

[14] S. Honda, K. Namekata, A. Kimura, X. Guo, C. Harada, A. Murakami, A. Matsuda, T. Harada, Survival of alpha and intrinsically photosensitive retinal ganglion cells in NMDA-induced neurotoxicity and a mouse model of normal tension glaucoma. Invest. Ophthalmol. Vis. Sci. 60, 3696–3707 (2019). 31487370 10.1167/iovs.19-27145

[15] W. K. Ju, K. Y. Kim, J. H. Cha, I. B. Kim, M. Y. Lee, S. J. Oh, J. W. Chung, M. H. Chun, Ganglion cells of the rat retina show osteopontin-like immunoreactivity. Brain Res. 852, 217–220 (2000). 10661516 10.1016/s0006-8993(99)02140-x

[16] G. Chidlow, J. P. M. Wood, J. Manavis, N. N. Osborne, R. J. Casson, Expression of osteopontin in the rat retina: Effects of excitotoxic and ischemic injuries. Invest. Ophthalmol. Vis. Sci. 49, 762–771 (2008). 18235026 10.1167/iovs.07-0726

[17] X. Duan, M. Qiao, F. Bei, I. J. Kim, Z. He, J. R. Sanes, Subtype-specific regeneration of retinal ganglion cells following axotomy: Effects of osteopontin and mTOR signaling. Neuron 85, 1244–1256 (2015). 25754821 10.1016/j.neuron.2015.02.017 PMC4391013

[18] M. Zhao, K. Toma, B. Kinde, L. Li, A. K. Patel, K. Y. Wu, M. R. Lum, C. Tan, J. E. Hooper, A. R. Kriegstein, A. La Torre, Y. J. Liao, D. S. Welsbie, Y. Hu, Y. Han, X. Duan, Osteopontin drives retinal ganglion cell resiliency in glaucomatous optic neuropathy. Cell Rep. 42, 113038 (2023). 37624696 10.1016/j.celrep.2023.113038 PMC10591811

[19] M. Y. Lee, J. S. Choi, S. W. Lim, J. H. Cha, M. H. Chun, J. W. Chung, Expression of osteopontin mRNA in developing rat brainstem and cerebellum. Cell Tissue Res. 306, 179–185 (2001). 11702229 10.1007/s004410100456

[20] T. Yamamoto, S. Murayama, M. Takao, T. Isa, N. Higo, Expression of secreted phosphoprotein 1 (osteopontin) in human sensorimotor cortex and spinal cord: Changes in patients with amyotrophic lateral sclerosis. Brain Res. 1655, 168–175 (2017). 27823929 10.1016/j.brainres.2016.10.030

[21] Y. Sugiyama, T. Oishi, A. Yamashita, Y. Murata, T. Yamamoto, I. Takashima, T. Isa, N. Higo, Neuronal and microglial localization of secreted phosphoprotein 1 (osteopontin) in intact and damaged motor cortex of macaques. Brain Res. 1714, 52–64 (2019). 30790559 10.1016/j.brainres.2019.02.021

[22] F. J. Mahmud, Y. Du, E. Greif, T. Boucher, R. F. Dannals, W. B. Mathews, M. G. Pomper, P. Sysa-Shah, K. A. Metcalf Pate, C. Lyons, B. Carlson, M. Chacona, A. M. Brown, Osteopontin/secreted phosphoprotein-1 behaves as a molecular brake regulating the neuroinflammatory response to chronic viral infection. J. Neuroinflammation 17, 273 (2020). 32943056 10.1186/s12974-020-01949-4 PMC7499959

[23] S. Li, T. C. Jakobs, Secreted phosphoprotein 1 slows neurodegeneration and rescues visual function in mouse models of aging and glaucoma. Cell Rep. 41, 111880 (2022). 36577373 10.1016/j.celrep.2022.111880 PMC9847489

[24] S. Li, T. C. Jakobs, Vitamin C protects retinal ganglion cells via SPP1 in glaucoma and after optic nerve damage. Life Sci. Alliance 6, e202301976 (2023). 37160307 10.26508/lsa.202301976 PMC10172762

[25] U. C. Drager, D. L. Edwards, C. J. Barnstable, Antibodies against filamentous components in discrete cell types of the mouse retina. J. Neurosci. 4, 2025–2042 (1984). 6381660 10.1523/JNEUROSCI.04-08-02025.1984 PMC6564963

[26] B. Krieger, M. Qiao, D. L. Rousso, J. R. Sanes, M. Meister, Four alpha ganglion cell types in mouse retina: Function, structure, and molecular signatures. PLOS ONE 12, e0180091 (2017). 28753612 10.1371/journal.pone.0180091 PMC5533432

[27] A. Gallego-Ortega, M. Norte-Munoz, J. Di Pierdomenico, M. Aviles-Trigueros, P. de la Villa, F. J. Valiente-Soriano, M. Vidal-Sanz, Alpha retinal ganglion cells in pigmented mice retina: number and distribution. Front. Neuroanat. 16, 1054849 (2022). 36530520 10.3389/fnana.2022.1054849 PMC9751430

[28] R. P. Kirwan, M. O. Leonard, M. Murphy, A. F. Clark, C. J. O’Brien, Transforming growth factor-β-regulated gene transcription and protein expression in human GFAP-negative lamina cribrosa cells. Glia 52, 309–324 (2005). 16078232 10.1002/glia.20247

[29] U. M. Malyankar, M. Almeida, R. J. Johnson, R. H. Pichler, C. M. Giachelli, Osteopontin regulation in cultured rat renal epithelial cells. Kidney Int. 51, 1766–1773 (1997). 9186865 10.1038/ki.1997.243

[30] A. Bosco, M. R. Steele, M. L. Vetter, Early microglia activation in a mouse model of chronic glaucoma. J. Comp. Neurol. 519, 599–620 (2011). 21246546 10.1002/cne.22516 PMC4169989

[31] A. Bosco, C. O. Romero, K. T. Breen, A. A. Chagovetz, M. R. Steele, B. K. Ambati, M. L. Vetter, Neurodegeneration severity can be predicted from early microglia alterations monitored in vivo in a mouse model of chronic glaucoma. Dis. Model. Mech. 8, 443–455 (2015). 25755083 10.1242/dmm.018788 PMC4415894

[32] L. Liaw, M. P. Skinner, E. W. Raines, R. Ross, D. A. Cheresh, S. M. Schwartz, C. M. Giachelli, The adhesive and migratory effects of osteopontin are mediated via distinct cell surface integrins. Role of alpha v beta 3 in smooth muscle cell migration to osteopontin in vitro. J. Clin. Invest. 95, 713–724 (1995). 7532190 10.1172/JCI117718 PMC295539

[33] G. F. Weber, S. Ashkar, H. Cantor, Interaction between CD44 and osteopontin as a potential basis for metastasis formation. Proc. Assoc. Am. Physicians 109, 1–9 (1997). 9010911

[34] I. Choi, Y. Zhang, S. P. Seegobin, M. Pruvost, Q. Wang, K. Purtell, B. Zhang, Z. Yue, Microglia clear neuron-released α-synuclein via selective autophagy and prevent neurodegeneration. Nat. Commun. 11, 1386 (2020). 32170061 10.1038/s41467-020-15119-w PMC7069981

[35] I. Choi, M. Wang, S. Yoo, P. Xu, S. P. Seegobin, X. Li, X. Han, Q. Wang, J. Peng, B. Zhang, Z. Yue, Autophagy enables microglia to engage amyloid plaques and prevents microglial senescence. Nat. Cell Biol. 25, 963–974 (2023). 37231161 10.1038/s41556-023-01158-0 PMC10950302

[36] H. Iwashita, H. T. Sakurai, N. Nagahora, M. Ishiyama, K. Shioji, K. Sasamoto, K. Okuma, S. Shimizu, Y. Ueno, Small fluorescent molecules for monitoring autophagic flux. FEBS Lett. 592, 559–567 (2018). 29355929 10.1002/1873-3468.12979 PMC5947577

[37] H. T. Sakurai, H. Iwashita, S. Arakawa, A. Yikelamu, M. Kusaba, S. Kofuji, H. Nishina, M. Ishiyama, Y. Ueno, S. Shimizu, Development of small fluorescent probes for the analysis of autophagy kinetics. iScience 26, 107218 (2023). 37456828 10.1016/j.isci.2023.107218 PMC10339198

[38] D. Goldblum, A. Kipfer-Kauer, G. M. Sarra, S. Wolf, B. E. Frueh, Distribution of amyloid precursor protein and amyloid-beta immunoreactivity in DBA/2J glaucomatous mouse retinas. Invest. Ophthalmol. Vis. Sci. 48, 5085–5090 (2007). 17962460 10.1167/iovs.06-1249

[39] J. Pilotte, S. Khoury, A. Tafreshi, Z. T. Mandel, S. V. Sharma, P. W. Vanderklish, S. T. Sarraf, A. A. Sadun, R. N. Weinreb, A. S. Huang, Identification of retinal amyloid-beta in ex vivo human glaucoma eyes using a novel ocular tracer. J. Glaucoma 34, e4–e8 (2025). 39283690 10.1097/IJG.0000000000002496

[40] N. Mizushima, Autophagy: process and function. Genes Dev. 21, 2861–2873 (2007). 18006683 10.1101/gad.1599207

[41] R. Stanzione, D. Pietrangelo, M. Cotugno, M. Forte, S. Rubattu, Role of autophagy in ischemic stroke: Insights from animal models and preliminary evidence in the human disease. Front. Cell Dev. Biol. 12, 1360014 (2024). 38590779 10.3389/fcell.2024.1360014 PMC10999556

[42] J. Lowe, A. B. Piepho, C. K. Gomatam, P. Debell, M. N. Ballinger, J. A. Rafael-Fortney, Mineralocorticoid receptor antagonists reduce inflammatory signaling independent of myofiber mineralocorticoid receptor. Endocr. Metab. Sci. 19, 100266 (2025). 41497323 10.1016/j.endmts.2025.100266 PMC12768496

[43] A. Saviano, B. Apta, S. Tull, L. Pezhman, A. Fatima, M. Sevim, A. Mete, M. Chimen, A. Schettino, N. Marigliano, H. M. McGettrick, A. J. Iqbal, F. Maione, G. E. Rainger, PEPITEM, its tripeptide pharmacophores and their peptidomimetic analogues regulate the inflammatory response through parenteral and topical dosing in models of peritonitis and psoriasis. Pharmacol. Res. 213, 107624 (2025). 39855372 10.1016/j.phrs.2025.107624

[44] D.-S. Son, K. A. Done, J. Son, M. G. Izban, C. Virgous, E. S. Lee, S. E. Adunyah, Intermittent fasting attenuates obesity-induced triple-negative breast cancer progression by disrupting cell cycle, epithelial-mesenchymal transition, immune contexture, and proinflammatory signature. Nutrients 16, 2101 (2024). 38999849 10.3390/nu16132101 PMC11243652

[45] J. Tong, X. Yan, L. Yu, The late stage of autophagy: cellular events and molecular regulation. Protein Cell 1, 907–915 (2010). 21204017 10.1007/s13238-010-0121-z PMC4875124

[46] D. K. Sidibe, M. C. Vogel, S. Maday, Organization of the autophagy pathway in neurons. Curr. Opin. Neurobiol. 75, 102554 (2022). 35649324 10.1016/j.conb.2022.102554 PMC9990471

[47] F. Ahmadi-Dehlaghi, P. Mohammadi, E. Valipour, P. Pournaghi, S. Kiani, K. Mansouri, Autophagy: A challengeable paradox in cancer treatment. Cancer Med. 12, 11542–11569 (2023). 36760166 10.1002/cam4.5577 PMC10242330

[48] J. S. Park, D. H. Kim, S. Y. Yoon, Regulation of amyloid precursor protein processing by its KFERQ motif. BMB Rep. 49, 337–343 (2016). 26779997 10.5483/BMBRep.2016.49.6.212 PMC5070722

[49] R. Yao, J. Shen, Chaperone-mediated autophagy: Molecular mechanisms, biological functions, and diseases. MedComm 4, e347 (2023). 37655052 10.1002/mco2.347 PMC10466100

[50] Z. Deng, K. Purtell, V. Lachance, M. S. Wold, S. Chen, Z. Yue, Autophagy receptors and neurodegenerative diseases. Trends Cell Biol. 27, 491–504 (2017). 28169082 10.1016/j.tcb.2017.01.001

[51] N. Mizushima, T. Noda, T. Yoshimori, Y. Tanaka, T. Ishii, M. D. George, D. J. Klionsky, M. Ohsumi, Y. Ohsumi, A protein conjugation system essential for autophagy. Nature 395, 395–398 (1998). 9759731 10.1038/26506

[52] L. Peichl, H. Wassle, The structural correlate of the receptive field centre of alpha ganglion cells in the cat retina. J. Physiol. 341, 309–324 (1983). 6620182 10.1113/jphysiol.1983.sp014807 PMC1195336

[53] M. Watanabe, R. W. Rodieck, Parasol and midget ganglion cells of the primate retina. J. Comp. Neurol. 289, 434–454 (1989). 2808778 10.1002/cne.902890308

[54] J. D. Crook, B. B. Peterson, O. S. Packer, F. R. Robinson, J. B. Troy, D. M. Dacey, Y-cell receptive field and collicular projection of parasol ganglion cells in macaque monkey retina. J. Neurosci. 28, 11277–11291 (2008). 18971470 10.1523/JNEUROSCI.2982-08.2008 PMC2778053

[55] B. B. Boycott, H. Wassle, The morphological types of ganglion cells of the domestic cat’s retina. J. Physiol. 240, 397–419 (1974). 4422168 10.1113/jphysiol.1974.sp010616 PMC1331022

[56] L. Peichl, E. H. Buhl, B. B. Boycott, Alpha ganglion cells in the rabbit retina. J. Comp. Neurol. 263, 25–41 (1987). 2444630 10.1002/cne.902630103

[57] L. Peichl, Alpha ganglion cells in mammalian retinae: common properties, species differences, and some comments on other ganglion cells. Vis. Neurosci. 7, 155–169 (1991). 1931799 10.1017/s0952523800011020

[58] F. Wang, E. Li, L. De, Q. Wu, Y. Zhang, OFF-transient alpha RGCs mediate looming triggered innate defensive response. Curr. Biol. 31, 2263–2273.e3 (2021). 33798432 10.1016/j.cub.2021.03.025

[59] T. Lee, H. Weinberg-Wolf, T. E. Zapadka, A. Rudenko, J. B. Demb, I. J. Kim, Specific retinal neurons regulate context-dependent defensive responses to visual threat. PNAS Nexus 3, pgae423 (2024). 39359403 10.1093/pnasnexus/pgae423 PMC11443969

[60] N. Cui, J. Jia, Y. He, Glaucomatous retinal ganglion cells: death and protection. Int. J. Ophthalmol. 18, 160–167 (2025). 39829615 10.18240/ijo.2025.01.20 PMC11672089

[61] G. S. Zode, A. B. Sharma, X. Lin, C. C. Searby, K. Bugge, G. H. Kim, A. F. Clark, V. C. Sheffield, Ocular-specific ER stress reduction rescues glaucoma in murine glucocorticoid-induced glaucoma. J. Clin. Invest. 124, 1956–1965 (2014). 24691439 10.1172/JCI69774 PMC4001532

[62] A. Venkatesan, A. M. Bernstein, Protein misfolding and mitochondrial dysfunction in glaucoma. Front. Cell Dev. Biol. 13, 1595121 (2025). 40385286 10.3389/fcell.2025.1595121 PMC12083186

[63] Z. Yan, H. Liao, H. Chen, S. Deng, Y. Jia, C. Deng, J. Lin, J. Ge, Y. Zhuo, Elevated intraocular pressure induces amyloid-β deposition and tauopathy in the lateral geniculate nucleus in a monkey model of glaucoma. Invest. Ophthalmol. Vis. Sci. 58, 5434–5443 (2017). 29059309 10.1167/iovs.17-22312

[64] S. Madhoun, M. T. C. Martins, A. Korneva, T. V. Johnson, E. Kimball, S. Quillen, M. E. Pease, M. Edwards, H. Quigley, Effects of experimental glaucoma in Lama1(nmf223) mutant mice. Exp. Eye Res. 226, 109341 (2023). 36476399 10.1016/j.exer.2022.109341 PMC10204621

[65] M.-H. Cho, K. Cho, H.-J. Kang, E.-Y. Jeon, H.-S. Kim, H.-J. Kwon, H.-M. Kim, D.-H. Kim, S.-Y. Yoon, Autophagy in microglia degrades extracellular β-amyloid fibrils and regulates the NLRP3 inflammasome. Autophagy 10, 1761–1775 (2014). 25126727 10.4161/auto.29647 PMC4198361

[66] J.-H. Lee, D.-S. Yang, C. N. Goulbourne, E. Im, P. Stavrides, A. Pensalfini, H. Chan, C. Bouchet-Marquis, C. Bleiwas, M. J. Berg, C. Huo, J. Peddy, M. Pawlik, E. Levy, M. Rao, M. Staufenbiel, R. A. Nixon, Faulty autolysosome acidification in Alzheimer’s disease mouse models induces autophagic build-up of Aβ in neurons, yielding senile plaques. Nat. Neurosci. 25, 688–701 (2022). 35654956 10.1038/s41593-022-01084-8 PMC9174056

[67] S. Estfanous, K. P. Daily, M. Eltobgy, N. P. Deems, M. N. K. Anne, K. Krause, A. Badr, K. Hamilton, C. Carafice, A. Hegazi, A. Abu Khweek, H. Kelani, S. Nimjee, H. Awad, X. Zhang, E. Cormet-Boyaka, H. Haffez, S. Soror, A. Mikhail, G. Nuovo, R. M. Barrientos, M. A. Gavrilin, A. O. Amer, Elevated expression of MiR-17 in microglia of Alzheimer’s disease patients abrogates autophagy-mediated Amyloid-β degradation. Front. Immunol. 12, 705581 (2021). 34426734 10.3389/fimmu.2021.705581 PMC8379081

[68] S. Pankiv, T. H. Clausen, T. Lamark, A. Brech, J. A. Bruun, H. Outzen, A. Overvatn, G. Bjorkoy, T. Johansen, p62/SQSTM1 binds directly to Atg8/LC3 to facilitate degradation of ubiquitinated protein aggregates by autophagy. J. Biol. Chem. 282, 24131–24145 (2007). 17580304 10.1074/jbc.M702824200

[69] V. Kirkin, T. Lamark, Y. S. Sou, G. Bjorkoy, J. L. Nunn, J. A. Bruun, E. Shvets, D. G. McEwan, T. H. Clausen, P. Wild, I. Bilusic, J. P. Theurillat, A. Overvatn, T. Ishii, Z. Elazar, M. Komatsu, I. Dikic, T. Johansen, A role for NBR1 in autophagosomal degradation of ubiquitinated substrates. Mol. Cell 33, 505–516 (2009). 19250911 10.1016/j.molcel.2009.01.020

[70] P. Wild, H. Farhan, D. G. McEwan, S. Wagner, V. V. Rogov, N. R. Brady, B. Richter, J. Korac, O. Waidmann, C. Choudhary, V. Dotsch, D. Bumann, I. Dikic, Phosphorylation of the autophagy receptor optineurin restricts Salmonella growth. Science 333, 228–233 (2011). 21617041 10.1126/science.1205405 PMC3714538

[71] M. A. Sanjuan, C. P. Dillon, S. W. G. Tait, S. Moshiach, F. Dorsey, S. Connell, M. Komatsu, K. Tanaka, J. L. Cleveland, S. Withoff, D. R. Green, Toll-like receptor signalling in macrophages links the autophagy pathway to phagocytosis. Nature 450, 1253–1257 (2007). 18097414 10.1038/nature06421

[72] B. L. Heckmann, B. J. W. Teubner, B. Tummers, E. Boada-Romero, L. Harris, M. Yang, C. S. Guy, S. S. Zakharenko, D. R. Green, LC3-associated endocytosis facilitates β-amyloid clearance and mitigates neurodegeneration in murine Alzheimer’s disease. Cell 178, 536–551.e14 (2019). 31257024 10.1016/j.cell.2019.05.056 PMC6689199

[73] B. L. Heckmann, B. J. W. Teubner, E. Boada-Romero, B. Tummers, C. Guy, P. Fitzgerald, U. Mayer, S. Carding, S. S. Zakharenko, T. Wileman, D. R. Green, Noncanonical function of an autophagy protein prevents spontaneous Alzheimer’s disease. Sci. Adv. 6, eabb9036 (2020). 32851186 10.1126/sciadv.abb9036 PMC7428329

[74] M. P. Duyao, A. B. Auerbach, A. Ryan, F. Persichetti, G. T. Barnes, S. M. McNeil, P. Ge, J. P. Vonsattel, J. F. Gusella, A. L. Joyner, M. E. MacDonald, Inactivation of the mouse Huntington’s disease gene homolog Hdh. Science 269, 407–410 (1995). 7618107 10.1126/science.7618107

[75] R. M. Bragg, E. W. Mathews, A. Grindeland, J. P. Cantle, D. Howland, T. Vogt, J. B. Carroll, Global huntingtin knockout in adult mice leads to fatal neurodegeneration that spares the pancreas. Life Sci. Alliance 7, e202402571 (2024). 39054288 10.26508/lsa.202402571 PMC11272958

[76] L. A. Orosco, A. P. Ross, S. L. Cates, S. E. Scott, D. Wu, J. Sohn, D. Pleasure, S. J. Pleasure, I. E. Adamopoulos, K. S. Zarbalis, Loss of Wdfy3 in mice alters cerebral cortical neurogenesis reflecting aspects of the autism pathology. Nat. Commun. 5, 4692 (2014). 25198012 10.1038/ncomms5692 PMC4159772

[77] A. M. Whiteley, M. A. Prado, S. A. H. de Poot, J. A. Paulo, M. Ashton, S. Dominguez, M. Weber, H. Ngu, J. Szpyt, M. P. Jedrychowski, A. Easton, S. P. Gygi, T. Kurz, M. J. Monteiro, E. J. Brown, D. Finley, Global proteomics of Ubqln2-based murine models of ALS. J. Biol. Chem. 296, 100153 (2021). 33277362 10.1074/jbc.RA120.015960 PMC7873701

[78] K. Slowicka, L. Vereecke, C. Mc Guire, M. Sze, J. Maelfait, A. Kolpe, X. Saelens, R. Beyaert, G. van Loo, Optineurin deficiency in mice is associated with increased sensitivity to Salmonella but does not affect proinflammatory NF-κB signaling. Eur. J. Immunol. 46, 971–980 (2016). 26677802 10.1002/eji.201545863

[79] N. Ruzafa, X. Pereiro, P. Aspichueta, J. Araiz, E. Vecino, The retina of osteopontin deficient mice in aging. Mol. Neurobiol. 55, 213–221 (2018). 28866734 10.1007/s12035-017-0734-9 PMC5808060

[80] R. Meller, S. L. Stevens, M. Minami, J. A. Cameron, S. King, H. Rosenzweig, K. Doyle, N. S. Lessov, R. P. Simon, M. P. Stenzel-Poore, Neuroprotection by osteopontin in stroke. J. Cereb. Blood Flow Metab. 25, 217–225 (2005). 15678124 10.1038/sj.jcbfm.9600022

[81] K. P. Doyle, T. Yang, N. S. Lessov, T. M. Ciesielski, S. L. Stevens, R. P. Simon, J. S. King, M. P. Stenzel-Poore, Nasal administration of osteopontin peptide mimetics confers neuroprotection in stroke. J. Cereb. Blood Flow Metab. 28, 1235–1248 (2008). 18364727 10.1038/jcbfm.2008.17 PMC6015748

[82] C. Sun, B. Enkhjargal, C. Reis, T. Zhang, Q. Zhu, K. Zhou, Z. Xie, L. Wu, J. Tang, X. Jiang, J. H. Zhang, Osteopontin-enhanced autophagy attenuates early brain injury via FAK-ERK pathway and improves long-term outcome after subarachnoid hemorrhage in rats. Cells 8, 980 (2019). 31461955 10.3390/cells8090980 PMC6769958

[83] C. Sun, M. S. U. Rahman, B. Enkhjargal, J. Peng, K. Zhou, Z. Xie, L. Wu, T. Zhang, Q. Zhu, J. Tang, Y. Zeng, J. H. Zhang, S. Xu, Osteopontin modulates microglial activation states and attenuates inflammatory responses after subarachnoid hemorrhage in rats. Exp. Neurol. 371, 114585 (2024). 37884185 10.1016/j.expneurol.2023.114585

[84] A. Rentsendorj, J. Sheyn, D.-T. Fuchs, D. Daley, B. C. Salumbides, H. E. Schubloom, N. J. Hart, S. Li, E. Y. Hayden, D. B. Teplow, K. L. Black, Y. Koronyo, M. Koronyo-Hamaoui, A novel role for osteopontin in macrophage-mediated amyloid-β clearance in Alzheimer's models. Brain Behav. Immun. 67, 163–180 (2018). 28860067 10.1016/j.bbi.2017.08.019 PMC5865478

[85] Z. Azizan, M. Bazrgar, N. Bazgir, S. H. Moini, S. Ghaseminejad-Kermani, K. Safa, A. Eshaghian-Dorcheh, M. H. Harirchian, Osteopontin in Alzheimer’s disease: A double-edged sword in neurodegeneration and neuroprotection-A systematic review. CNS Neurosci. Ther. 31, e70269 (2025). 39957678 10.1111/cns.70269 PMC11831194

[86] Y. Qiu, X. Shen, O. Ravid, D. Atrakchi, D. Rand, A. E. Wight, H. J. Kim, S. Liraz-Zaltsman, I. Cooper, M. Schnaider Beeri, H. Cantor, Definition of the contribution of an Osteopontin-producing CD11c^+^ microglial subset to Alzheimer’s disease. Proc. Natl. Acad. Sci. U.S.A. 120, e2218915120 (2023). 36730200 10.1073/pnas.2218915120 PMC9963365

[87] H. Yu, H. Zhong, N. Li, K. Chen, J. Chen, J. Sun, L. Xu, J. Wang, M. Zhang, X. Liu, L. Deng, P. Huang, S. Huang, X. Shen, Y. Zhong, Osteopontin activates retinal microglia causing retinal ganglion cells loss via p38 MAPK signaling pathway in glaucoma. FASEB J. 35, e21405 (2021). 33559950 10.1096/fj.202002218R

[88] S. De Schepper, J. Z. Ge, G. Crowley, L. S. S. Ferreira, D. Garceau, C. E. Toomey, D. Sokolova, J. Rueda-Carrasco, S.-H. Shin, J. S. Kim, T. Childs, T. Lashley, J. J. Burden, M. Sasner, C. Sala Frigerio, S. Jung, S. Hong, Perivascular cells induce microglial phagocytic states and synaptic engulfment via SPP1 in mouse models of Alzheimer’s disease. Nat. Neurosci. 26, 406–415 (2023). 36747024 10.1038/s41593-023-01257-z PMC9991912

[89] Y. L. Chai, J. R. Chong, A. R. Raquib, X. Xu, S. Hilal, N. Venketasubramanian, B. Y. Tan, A. P. Kumar, G. Sethi, C. P. Chen, M. K. P. Lai, Plasma osteopontin as a biomarker of Alzheimer’s disease and vascular cognitive impairment. Sci. Rep. 11, 4010 (2021). 33597603 10.1038/s41598-021-83601-6 PMC7889621

[90] A. Dammak, J. Sanchez Naves, F. Huete-Toral, G. Carracedo, New biomarker combination related to oxidative stress and inflammation in primary open-angle glaucoma. Life 13, 1455 (2023). 37511830 10.3390/life13071455 PMC10381240

[91] S. Nikhalashree, R. George, B. Shantha, V. Lingam, W. Vidya, M. Panday, K. N. Sulochana, K. Coral, Detection of Proteins Associated with Extracellular Matrix Regulation in the Aqueous Humour of Patients with Primary Glaucoma. Curr. Eye Res. 44, 1018–1025 (2019). 30994369 10.1080/02713683.2019.1608261

[92] J. Temmerman, S. Engelborghs, M. Bjerke, M. D’Haeseleer, Cerebrospinal fluid inflammatory biomarkers for disease progression in Alzheimer’s disease and multiple sclerosis: a systematic review. Front. Immunol. 14, 1162340 (2023). 37520580 10.3389/fimmu.2023.1162340 PMC10374015

[93] A. Bedolla, E. Wegman, M. Weed, M. K. Stevens, K. Ware, A. Paranjpe, A. Alkhimovitch, I. Ifergan, A. Taranov, J. D. Peter, R. M. S. Gonzalez, J. E. Robinson, L. McClain, K. M. Roskin, N. H. Greig, Y. Luo, Adult microglial TGFβ1 is required for microglia homeostasis via an autocrine mechanism to maintain cognitive function in mice. Nat. Commun. 15, 5306 (2024). 38906887 10.1038/s41467-024-49596-0 PMC11192737

[94] G. L. McKinsey, N. Santander, X. Zhang, K. L. Kleemann, L. Tran, A. Katewa, K. Conant, M. Barraza, K. Waddell, C. O. Lizama, M. La Russa, J. H. Koo, H. Lee, D. Mukherjee, H. Paidassi, E. S. Anton, K. Atabai, D. Sheppard, O. Butovsky, T. D. Arnold, Radial glia integrin avb8 regulates cell autonomous microglial TGFβ1 signaling that is necessary for microglial identity. Nat. Commun. 16, 2840 (2025). 40121230 10.1038/s41467-025-57684-y PMC11929771

[95] T. Masuda, R. Sankowski, O. Staszewski, M. Prinz, Microglia heterogeneity in the single-cell era. Cell Rep. 30, 1271–1281 (2020). 32023447 10.1016/j.celrep.2020.01.010

[96] H. Maruyama, H. Morino, H. Ito, Y. Izumi, H. Kato, Y. Watanabe, Y. Kinoshita, M. Kamada, H. Nodera, H. Suzuki, O. Komure, S. Matsuura, K. Kobatake, N. Morimoto, K. Abe, N. Suzuki, M. Aoki, A. Kawata, T. Hirai, T. Kato, K. Ogasawara, A. Hirano, T. Takumi, H. Kusaka, K. Hagiwara, R. Kaji, H. Kawakami, Mutations of optineurin in amyotrophic lateral sclerosis. Nature 465, 223–226 (2010). 20428114 10.1038/nature08971

[97] T. Rezaie, A. Child, R. Hitchings, G. Brice, L. Miller, M. Coca-Prados, E. Heon, T. Krupin, R. Ritch, D. Kreutzer, R. P. Crick, M. Sarfarazi, Adult-onset primary open-angle glaucoma caused by mutations in optineurin. Science 295, 1077–1079 (2002). 11834836 10.1126/science.1066901

[98] Y. Minegishi, M. Nakayama, D. Iejima, K. Kawase, T. Iwata, Significance of optineurin mutations in glaucoma and other diseases. Prog. Retin. Eye Res. 55, 149–181 (2016). 27693724 10.1016/j.preteyeres.2016.08.002

[99] K. C. Huang, C. Gomes, Y. Shiga, N. Belforte, K. B. VanderWall, S. S. Lavekar, C. M. Fligor, J. Harkin, S. M. Hetzer, S. V. Patil, A. Di Polo, J. S. Meyer, Acquisition of neurodegenerative features in isogenic OPTN(E50K) human stem cell-derived retinal ganglion cells associated with autophagy disruption and mTORC1 signaling reduction. Acta Neuropathol. Commun. 12, 164 (2024). 39425218 10.1186/s40478-024-01872-2 PMC11487784

[100] C. F. Burgoyne, The non-human primate experimental glaucoma model. Exp. Eye Res. 141, 57–73 (2015). 26070984 10.1016/j.exer.2015.06.005 PMC4628879

[101] P. Chaudhary, C. Stowell, J. Reynaud, S. K. Gardiner, H. Yang, G. Williams, I. Williams, N. Marsh-Armstrong, C. F. Burgoyne, Optic nerve head myelin-related protein, GFAP, and Iba1 alterations in non-human primates with early to moderate experimental glaucoma. Invest. Ophthalmol. Vis. Sci. 63, 9 (2022). 10.1167/iovs.63.11.9 PMC9586137 36239974

[102] A. Lacy-Hulbert, A. M. Smith, H. Tissire, M. Barry, D. Crowley, R. T. Bronson, J. T. Roes, J. S. Savill, R. O. Hynes, Ulcerative colitis and autoimmunity induced by loss of myeloid alphav integrins. Proc. Natl. Acad. Sci. U.S.A. 104, 15823–15828 (2007). 17895374 10.1073/pnas.0707421104 PMC1994135

[103] D. Sun, M. Lye-Barthel, R. H. Masland, T. C. Jakobs, Structural remodeling of fibrous astrocytes after axonal injury. J. Neurosci. 30, 14008–14019 (2010). 20962222 10.1523/JNEUROSCI.3605-10.2010 PMC3124820

[104] R. M. Sappington, B. J. Carlson, S. D. Crish, D. J. Calkins, The microbead occlusion model: A paradigm for induced ocular hypertension in rats and mice. Invest. Ophthalmol. Vis. Sci. 51, 207–216 (2010). 19850836 10.1167/iovs.09-3947 PMC2869054

[105] H. Chen, X. Wei, K. S. Cho, G. Chen, R. Sappington, D. J. Calkins, D. F. Chen, Optic neuropathy due to microbead-induced elevated intraocular pressure in the mouse. Invest. Ophthalmol. Vis. Sci. 52, 36–44 (2011). 20702815 10.1167/iovs.09-5115 PMC3053285

[106] Y. Zhu, R. Wang, A. C. Pappas, P. Seifert, A. Savol, R. I. Sadreyev, D. Sun, T. C. Jakobs, Astrocytes in the optic nerve are heterogeneous in their reactivity to glaucomatous injury. Cells 12, 2131 (2023). 37681863 10.3390/cells12172131 PMC10486930

[107] M. Parekh, A. Miall, A. Chou, L. Buhl, N. Deshpande, M. O. Price, F. W. Price, U. V. Jurkunas, Enhanced migration of Fuchs corneal endothelial cells by Rho kinase inhibition: A novel ex vivo descemet’s stripping only model. Cells 13, 1218 (2024). 39056800 10.3390/cells13141218 PMC11274477

[108] H. Alemi, S. Wang, T. Blanco, F. Kahale, R. B. Singh, G. Ortiz, A. Musayeva, E. Yuksel, K. Pang, N. Deshpande, T. H. Dohlman, U. V. Jurkunas, J. Yin, R. Dana, The neuropeptide α-melanocyte-stimulating hormone prevents persistent corneal edema following injury. Am. J. Pathol. 194, 150–164 (2024). 37827217 10.1016/j.ajpath.2023.09.007 PMC10768537

