# Supplementary material for: Neuron-derived SPP1 instructs microglia to limit degeneration
Source: Sci Adv. 2026 Aug 28;12(35):eaee4940. doi: 10.1126/sciadv.aee4940 (PMC13524033; doi:10.1126/sciadv.aee4940)
Supplement: Supplementary file 1 [file sciadv.aee4940_sm.pdf]

Science Advances
AAAS

Supplementary Materials for

Neuron-derived SPP1 instructs microglia to limit degeneration

Song Li and Tatjana C. Jakobs

Corresponding author: Song Li, song_li@meei.harvard.edu; Tatjana C. Jakobs, tatjana_jakobs@meei.harvard.edu

Sci. Adv. **12**, eaee4940 (2026)
DOI: 10.1126/sciadv.aee4940

The PDF file includes:

Figs. S1 to S15
Tables S1 and S2
Legends for movies S1 to S6

Other Supplementary Material for this manuscript includes the following:

Movies S1 to S6

Fig. S1. SPP1 is selectively expressed in αRGCs but not in other retinal cell types.

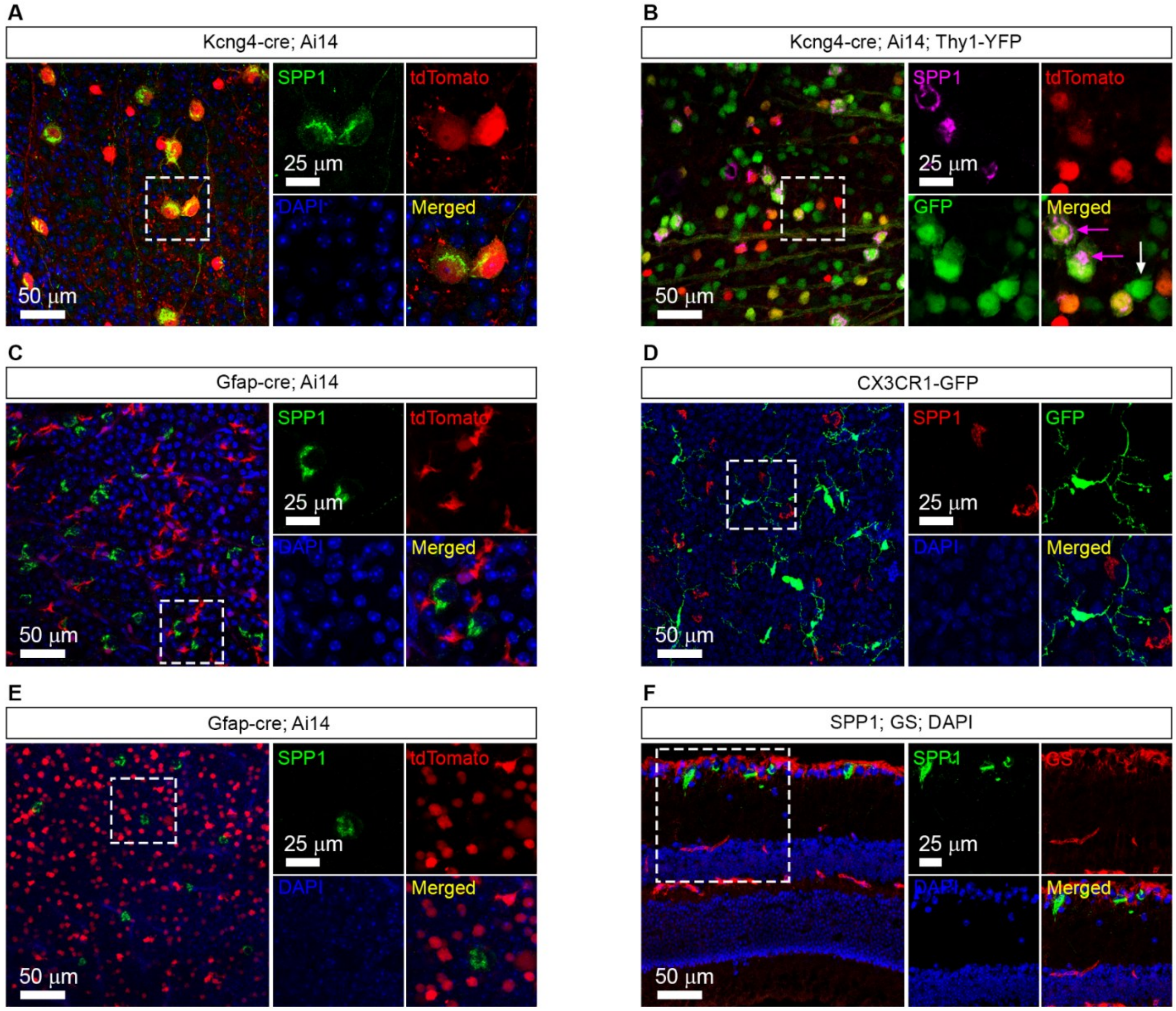

Fig. S1. SPP1 is selectively expressed in αRGCs but not in other retinal cell types.

(**A**) Kcng4-cre mice were crossed with the Ai14 reporter line to generate Kcng4-cre; Ai14 mice, in which αRGCs were labeled with tdTomato. Immunostaining showed strong co-localization of SPP1 with tdTomato^{+} αRGCs. (**B**) Kcng4-cre; Ai14 mice were crossed with Thy1-YFP mice to generate Kcng4-cre; Ai14; Thy1-YFP mice in which αRGCs were labeled with tdTomato and GFP, and non-αRGCs expressed GFP only. SPP1 co-localized with tdTomato^{+}GFP^{+} αRGCs but not with tdTomato^{-}GFP^{+} non-αRGCs. (**C**) To test for glial expression, GFAP-cre mice were crossed with Ai14 mice to generate GFAP-cre; Ai14 mice, in which astrocytes were labeled with tdTomato. SPP1 did not co-localize with tdTomato^{+} astrocytes. (**D**) Retinas from CX3CR1-GFP mice, in which microglia were labeled with GFP, showed no co-localization between SPP1 and GFP^{+} microglia. (**E**) In GFAP-cre; Ai14 mice, tdTomato^{+} Müller glia were observed in the inner nuclear layer; SPP1 staining showed no overlap with these tdTomato^{+} Müller cells. (**F**) Retina sections from C57BL/6 mice were stained with SPP1 and Müller cells marker glutamine synthetase (GS), confirming that SPP1 was not expressed in GS^{+} Müller cells.

Fig. S2. Conditional deletion of Spp1 in αRGCs of Spp1 cKO mice.

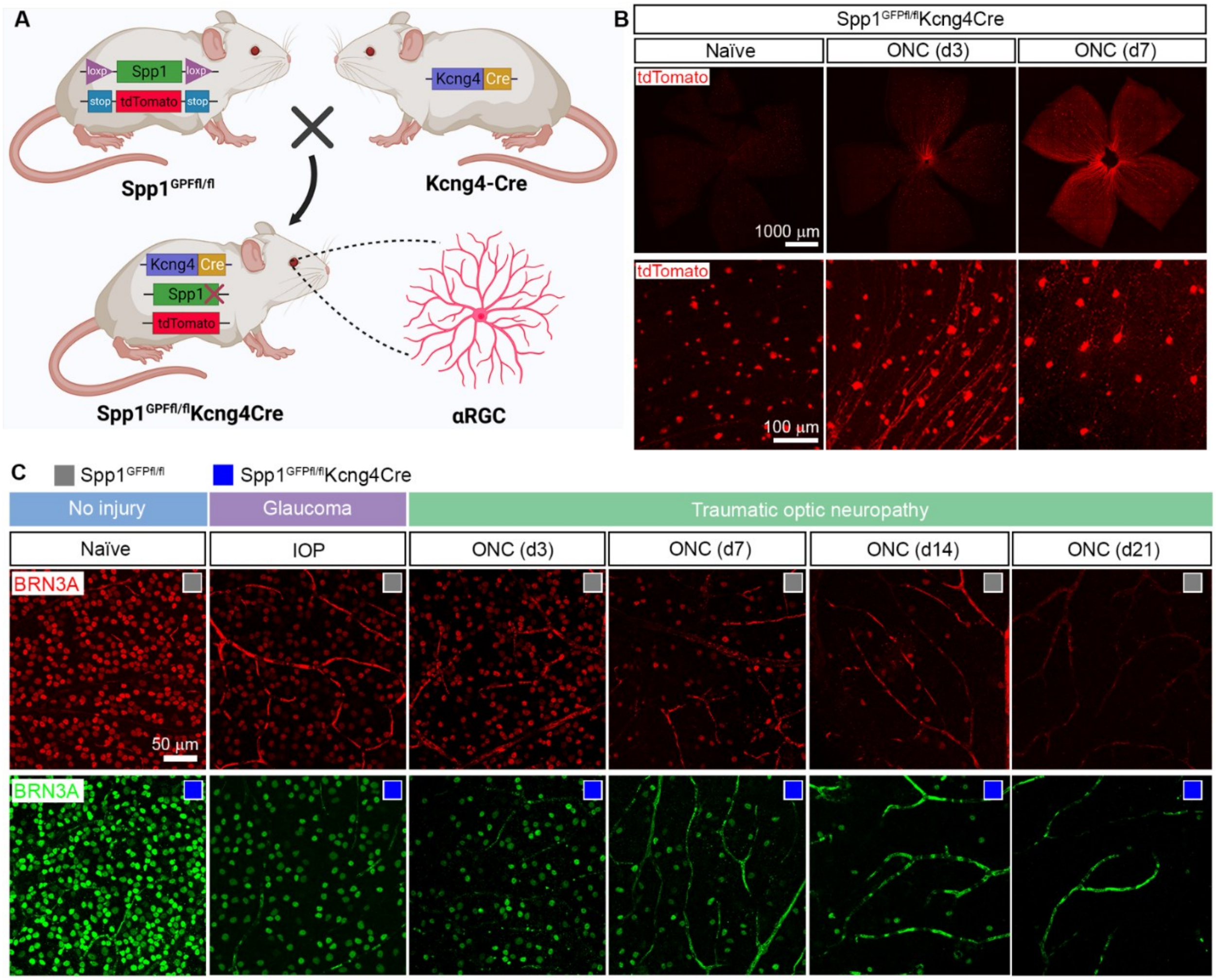

Fig. S2. Conditional deletion of Spp1 in αRGCs of Spp1 cKO mice.

(**A**) Schematic of the Spp1 conditional knockout (Spp1$^{GFPfl/fl}$Kcng4Cre) mouse line, generated by crossing Spp1$^{GFPfl/fl}$ mice with Kcng4-cre mice. In Spp1 cKO mice, Cre recombinase drives Spp1 deletion selectively in αRGCs, which are labeled with tdTomato fluorescence. (**B**) Representative confocal image showing tdTomato^{+} αRGCs in the retina of Spp1 cKO mice, confirming specific Cre activity in αRGCs. (**C**) Immunostaining for the non-αRGC marker BRN3A in retinas from Spp1$^{GFPfl/fl}$ (control) and Spp1$^{GFPfl/fl}$Kcng4Cre (Spp1 cKO) mice under naïve conditions, 4 weeks after intraocular pressure (IOP) elevation, and at days 3, 7, 14, and 21 after optic nerve crush (ONC). Diagram (A) was created in BioRender. Li, S. (2026) https://BioRender.com/bwb1e0v.

Fig. S3. Global Spp1 deletion aggravates microglial activation in glaucoma and optic nerve injury.

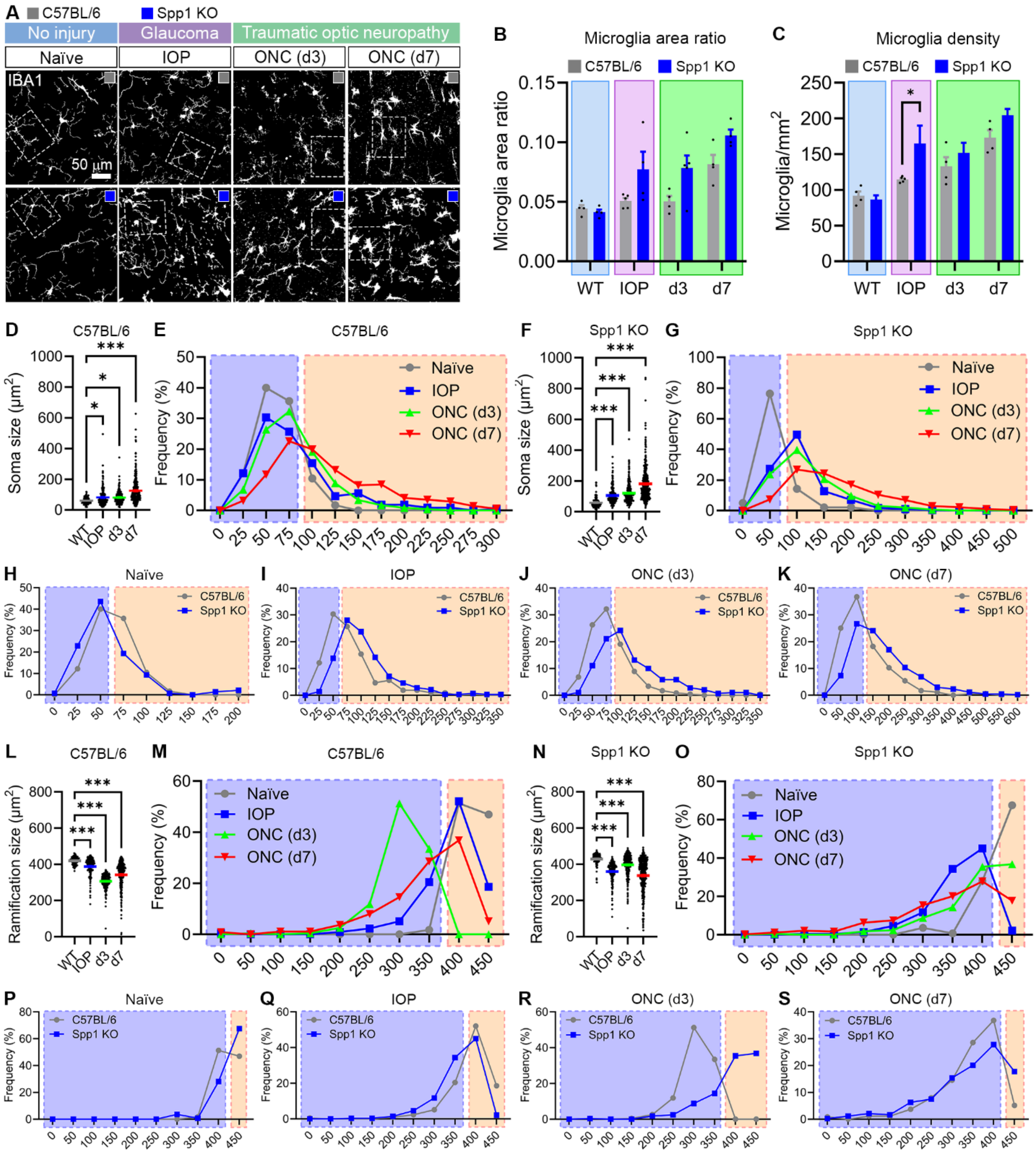

Fig. S3. Global Spp1 deletion aggravates microglial activation in glaucoma and optic nerve injury.

(A) Representative images showing IBA1⁺ microglia in retinas of C57BL/6 (WT) and Spp1 KO mice under naïve conditions, 4 weeks after IOP elevation, and on days 3 and 7 after ONC (n = 4–5 mice). Note: The region labeled with the dashed white box indicates the area shown at higher magnification in Fig. 2A. **(B** and **C)** Microglial area ratio (B) and density (C) in WT and Spp1 KO retinas under the same conditions (n = 4–5 mice). **(D** and **E)** Quantification (D) and Scholl analysis (E) of microglial soma size in WT retinas under naïve, IOP, and ONC conditions (n = 115–352 cells). **(F** and **G)** Quantification (F) and Scholl analysis (G) of microglial soma size in Spp1 KO retinas across same conditions (n = 140–438 cells). **(H–K)** Scholl analysis of microglial soma size in WT and Spp1 KO retinas under naïve conditions (H), after IOP elevation (I), and on days 3 (J) and 7 (K) after ONC (n = 115–438 cells). **(L** and **M)** Quantification (L) and Scholl analysis (M) of microglial ramification size in WT retinas under naïve, IOP, and ONC conditions (n = 115–352 cells). **(N** and **O)** Quantification (N) and Scholl analysis (O) of microglial ramification size in Spp1 KO retinas under same conditions (n = 140–438 cells). **(P–S)** Scholl analysis of microglial ramification size in WT and Spp1 KO retinas under naïve conditions (P), after IOP elevation (Q), and on days 3 (R) and 7 (S) after ONC (n = 115–438 cells). The *P* values were calculated by two-way ANOVA (B and C) or one-way ANOVA (D, F, L, and N); $*p < 0.05$, $**p < 0.01$, $***p < 0.001$. Data are mean ± SEM.

Fig. S4. Conditional deletion of Spp1 in αRGCs increases microglial activation in glaucoma and optic nerve injury.

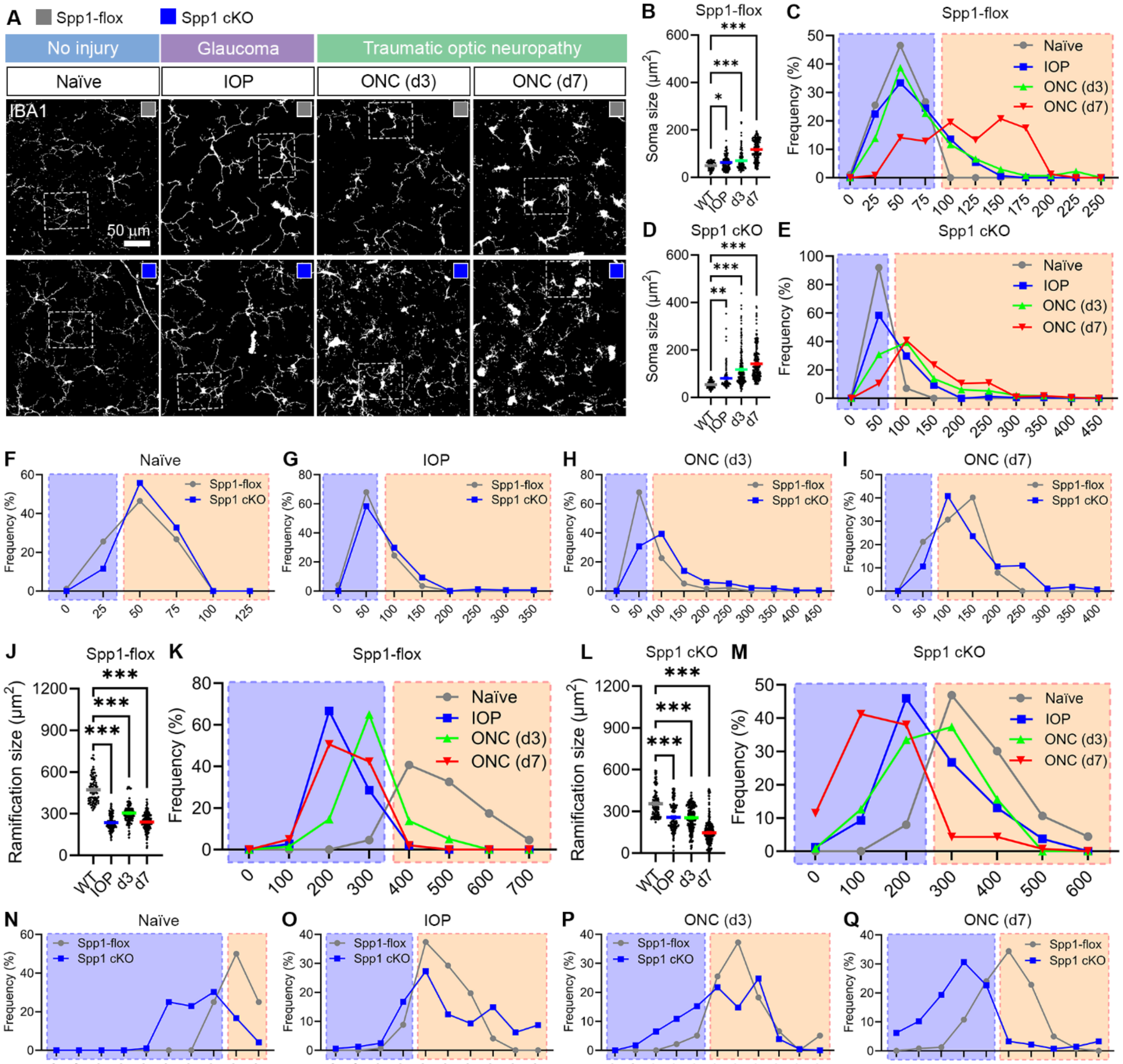

Fig. S4. Conditional deletion of Spp1 in αRGCs increases microglial activation in glaucoma and optic nerve injury.

(A) Representative images showing IBA1$^+$ microglia in retinas of Spp1-flox and Spp1 cKO mice 4 weeks after IOP elevation and on days 3 and 7 after ONC (n = 3–4 mice). Note: The region labeled with the dashed white box indicates the area shown at higher magnification in Fig. 2F. **(B and C)** Quantification (B) and Scholl analysis (C) of microglial soma size in Spp1-flox retinas 4 weeks after IOP elevation and on days 3 and 7 after ONC (n = 86–241 cells). **(D and E)** Quantification (D) and Scholl analysis (E) of microglial soma size in Spp1 cKO retinas across the same conditions (n = 113–284 cells). **(F–I)** Scholl analysis of microglial soma size in Spp1-flox and Spp1 cKO retinas under naïve conditions (F), after IOP elevation (G), and on day 3 (H) and day 7 (I) after ONC (n = 86–284 cells). **(J and K)** Quantification (J) and Scholl analysis (K) of microglial ramification size in Spp1-flox retinas under the same conditions (n = 86–241 cells). **(L and M)** Quantification (L) and Scholl analysis (M) of microglial ramification size in Spp1 cKO retinas under the same conditions (n = 113–284 cells). **(N–Q)** Scholl analysis of microglial ramification size in Spp1-flox and Spp1 cKO retinas under naïve conditions (N), after IOP elevation (O), and on day 3 (P) and day 7 (Q) after ONC (n = 86–284 cells). The *P* values were calculated by one-way ANOVA (B, D, J, and L); *$p < 0.05$, **$p < 0.01$, ***$p < 0.001$. Data are mean ± SEM.

Fig. S5. Microglial ItgαV deficiency exacerbates microglial activation and RGC loss in glaucoma and traumatic optic injury.

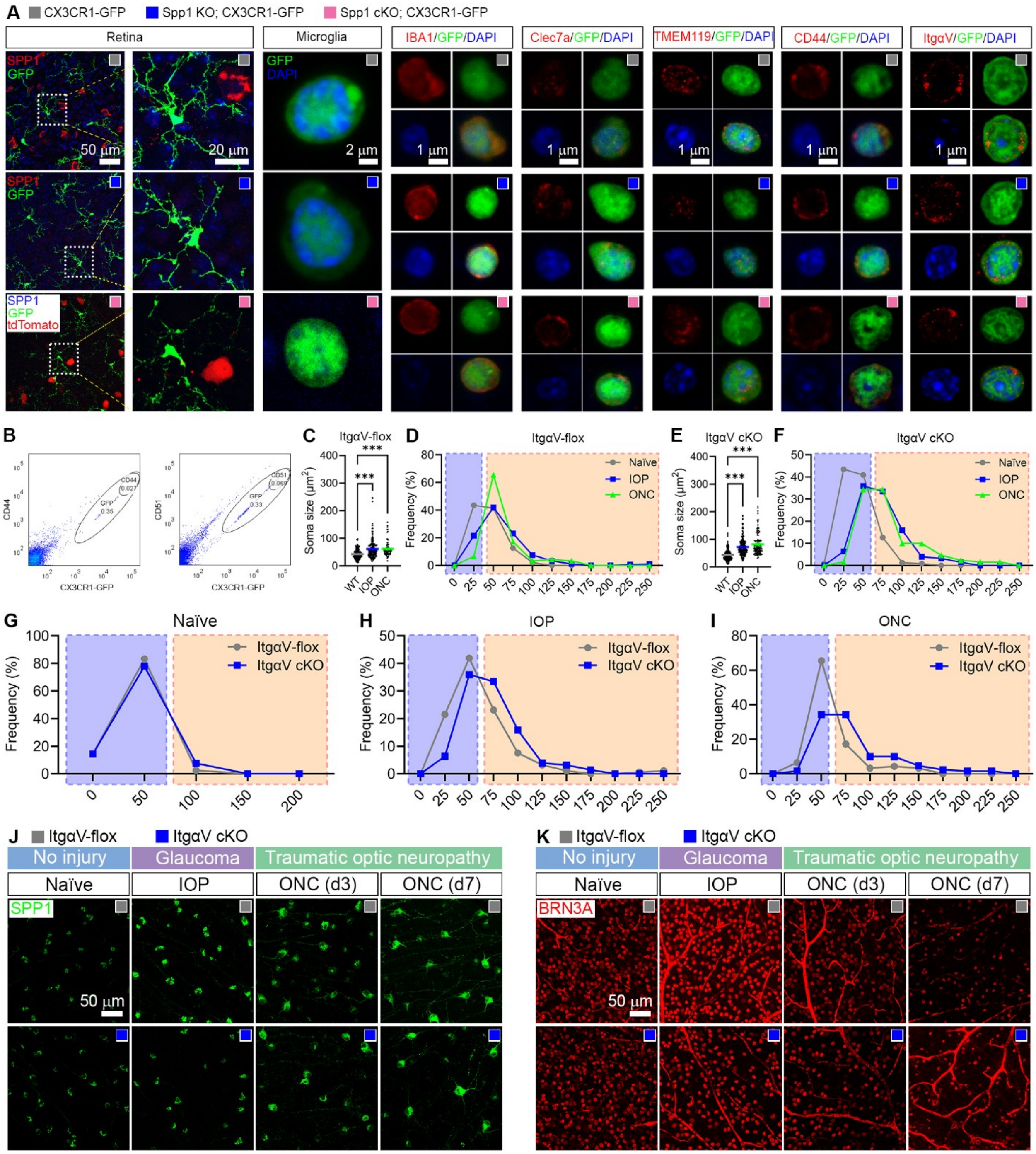

Fig. S5. Microglial ItgαV deficiency exacerbates microglial activation and RGC loss in glaucoma and traumatic optic injury.

(A) Representative images showing GFP^+ microglia in retinas from CX3CR1-GFP, Spp1 KO; CX3CR1-GFP, and Spp1 cKO; CX3CR1-GFP mice. Retinas were stained with SPP1. GFP^+ microglia were sorted by FACS and stained for IBA1, Clec7a, and TMEM119, as well as the SPP1 receptors CD44 and ItgαV (n = 6–10 cells for GFP, 6–12 for IBA1, 5–8 for Clec7a, 5–6 for TMEM119, 3–6 for CD44, and 3–45 for ItgαV). **(B)** FACS sorting of $CD44^+GFP^+$ and $ItgαV^+GFP^+$ microglia from CX3CR1-GFP retinas. **(C and D)** Quantification (C) and Scholl analysis (D) of microglial soma size in ItgαV-flox retinas 4 weeks after IOP elevation and on day 3 after ONC (n = 93–239 cells). **(E and F)** Quantification (E) and Scholl analysis (F) of microglial soma size in ItgαV cKO retinas under the same conditions (n = 131–284 cells). **(G–I)** Scholl analysis of microglial soma size in ItgαV-flox and ItgαV cKO retinas under naïve conditions (G), after IOP elevation (H), and on day 3 after ONC (I) (n = 93–284 cells). **(J and K)** Representative images of SPP1-labeled αRGCs (J) and BRN3A-labeled non-RGCs (K) in ItgαV-flox and ItgαV cKO retinas 4 weeks after IOP elevation and on days 3 and 7 after ONC (n = 6–9 mice). The *P* values were calculated by one-way ANOVA (B and D); $***p < 0.001$. Data are mean ± SEM.

Fig. S6. Microglial phagocytosis is unaffected by Spp1 deletion.

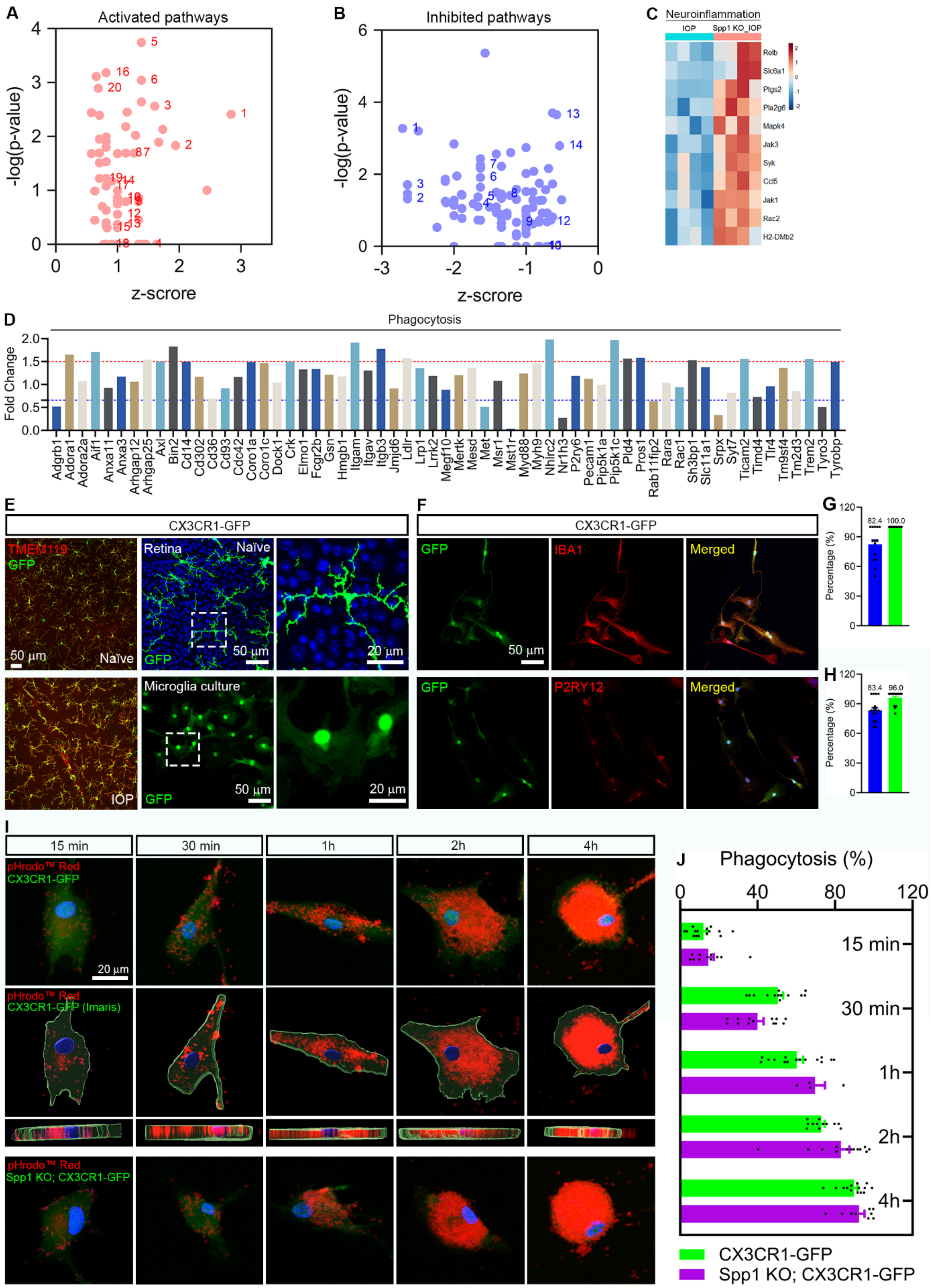

Fig. S6. Microglial phagocytosis is unaffected by Spp1 deletion.

(**A** and **B**) Volcano plots showing the activated (A) and inhibited (B) pathways in Spp1 KO microglia at 4 weeks after IOP elevation compared to WT microglia. Numbered pathways indicated the neuroinflammation-related pathways in A or autophagy-related pathways in B. (**C**) Heat map of neuroinflammation genes in Spp1 KO versus WT microglia after IOP elevation. (**D**) Expression of phagocytosis-related genes in Spp1 KO versus WT microglia after IOP elevation. (**E**) TMEM119 immunostaining in adult CX3CR1-GFP mouse retinas under naïve and high IOP conditions. Primary cultured GFP^+ microglia from retinas of P1-P3 neonatal CX3CR1-GFP mice. **(F–H)** Immunostaining of GFP^+ microglia with IBA1 and P2RY12 (F) and quantification of $IBA1^+$ (G) and $P2RY12^+$ (H) percentages (n = 14–18 wells). (**I**) Phagocytosis analysis of cultured WT and Spp1 KO microglia incubated with pHrodo red BioParticles for 15 min, 30 min, 1h, 2h, and 4h. 3D reconstructions were generated using Imaris. (**J**) Quantification of phagocytosis function in WT and Spp1 KO microglia (n = 4–15 cells).

Fig. S7. Autophagosome formation is impaired by Spp1 or ItgαV deletion in glaucoma and traumatic optic injury.

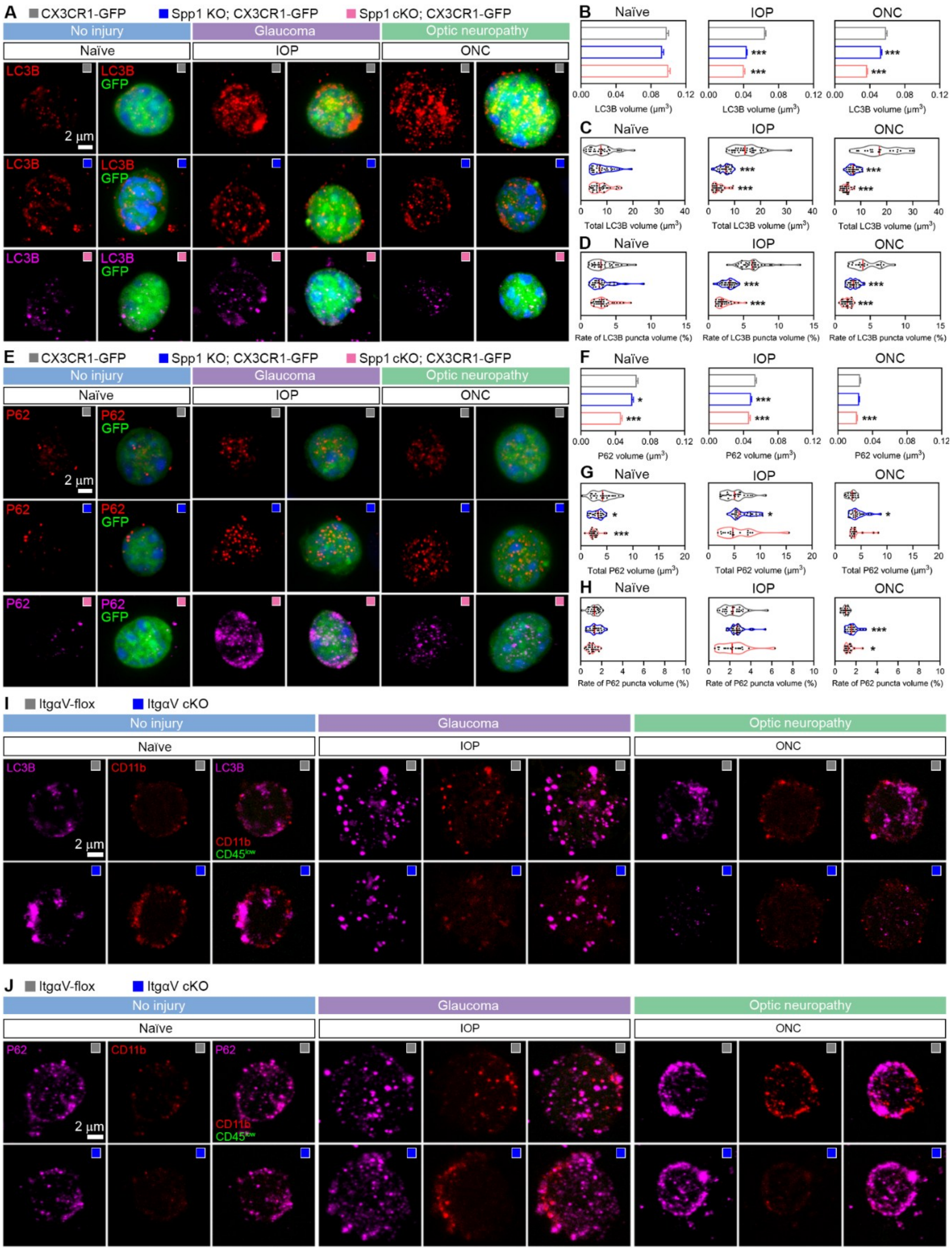

Fig. S7. Autophagosome formation is impaired by Spp1 or ItgαV deletion in glaucoma and traumatic optic injury.

(**A**) LC3B immunostaining in FACS-sorted GFP$^+$ microglia from CX3CR1-GFP, Spp1 KO; CX3CR1-GFP, and Spp1 cKO; CX3CR1-GFP mice under naïve condition, after 4 weeks of IOP elevation, and on day 7 post-ONC. (**B–D**) Quantification of LC3B puncta volume (B), total volume per cell (C), and volume percentage (D) (n = 2442–8017 puncta in B; n = 21–36 cells in C and D). (**E**) P62 immunostaining in GFP$^+$ microglia from CX3CR1-GFP, Spp1 KO; CX3CR1-GFP, and Spp1 cKO; CX3CR1-GFP mice under naïve condition, after 4 weeks of IOP elevation, and on day 7 post-ONC. **(F–H)** Quantification of P62 puncta volume (F), total volume (G), and volume percentage (H) (n = 1298–4102 puncta in F; n = 18–30 cells in G and H). (**I** and **J**) LC3B (I) and P62 (J) staining in FACS-sorted CD11b$^+$CD45^{low} microglia from ItgαV-flox and Itgα cKO retinas under naïve condition, after 4 weeks of IOP elevation, and on day 7 post-ONC. The *P* values were calculated by one-way ANOVA (B–D and F–H); **p* < 0.05, ***p* < 0.01, ****p* < 0.001. Data are mean ± SEM.

Fig. S8. Spp1 deletion impairs microglial clearance of Aβ in vivo and in vitro.

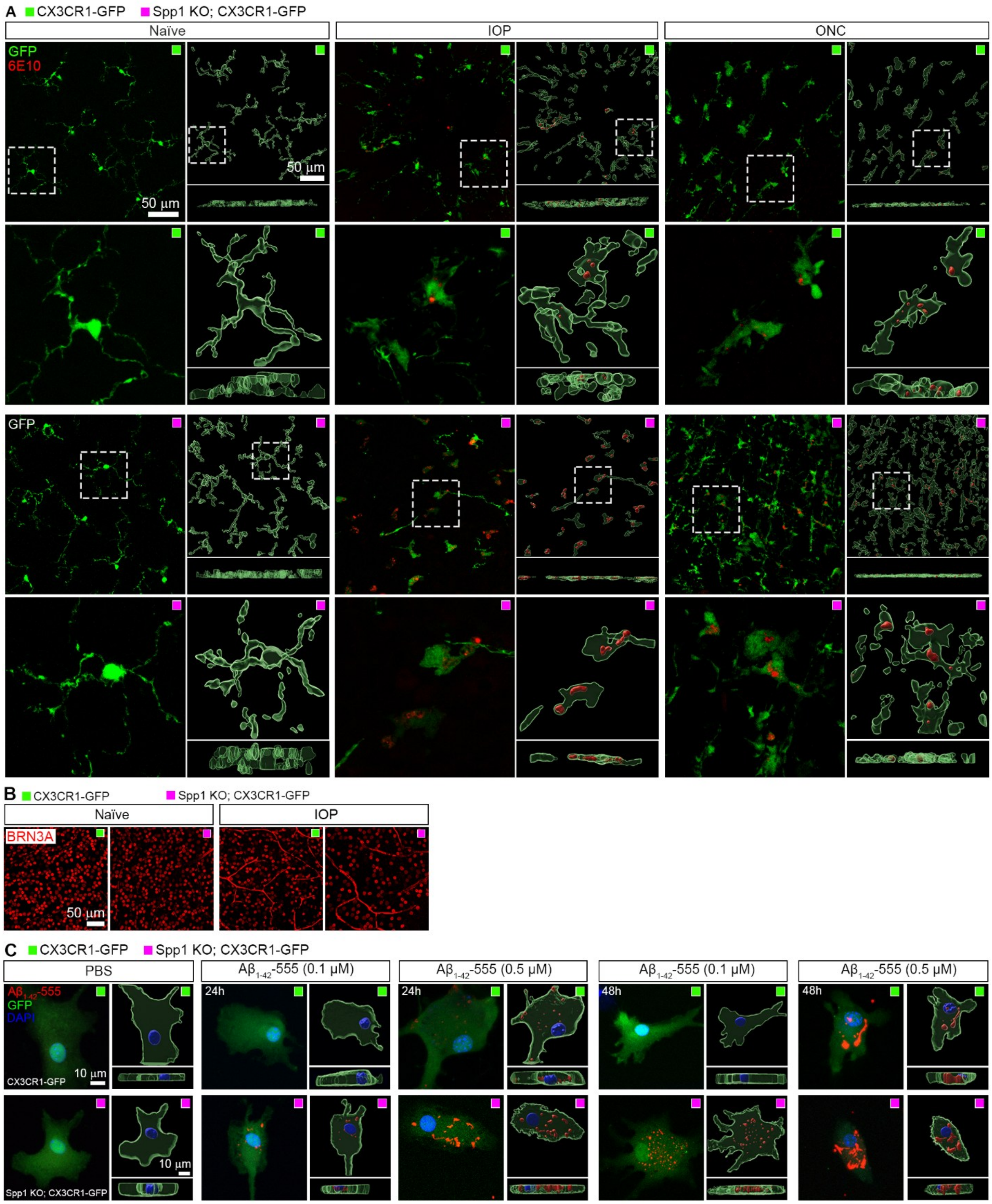

Fig. S8. Spp1 deletion impairs microglial clearance of Aβ in vivo and in vitro.

(**A**) GFP^+ microglia with Aβ accumulation (6E10 staining) in CX3CR1-GFP and Spp1 KO; CX3CR1-GFP retinas at 4 weeks after IOP elevation and on day 7 after ONC. GFP^+ microglia and Aβ aggregates were 3D-reconstructed using Imaris. (**B**) BRN3A-labeled RGCs in retinas at 4 weeks after IOP elevation. (**C**) Cultured WT and Spp1 KO GFP^+ microglia treated with Aβ protein ($Aβ_{1-42}$-555) at 0.1 μM and 0.5 μM for 24h or 48h and reconstructed by Imaris.

Fig. S9. Spp1 deletion causes microglial debris accumulation in glaucoma.

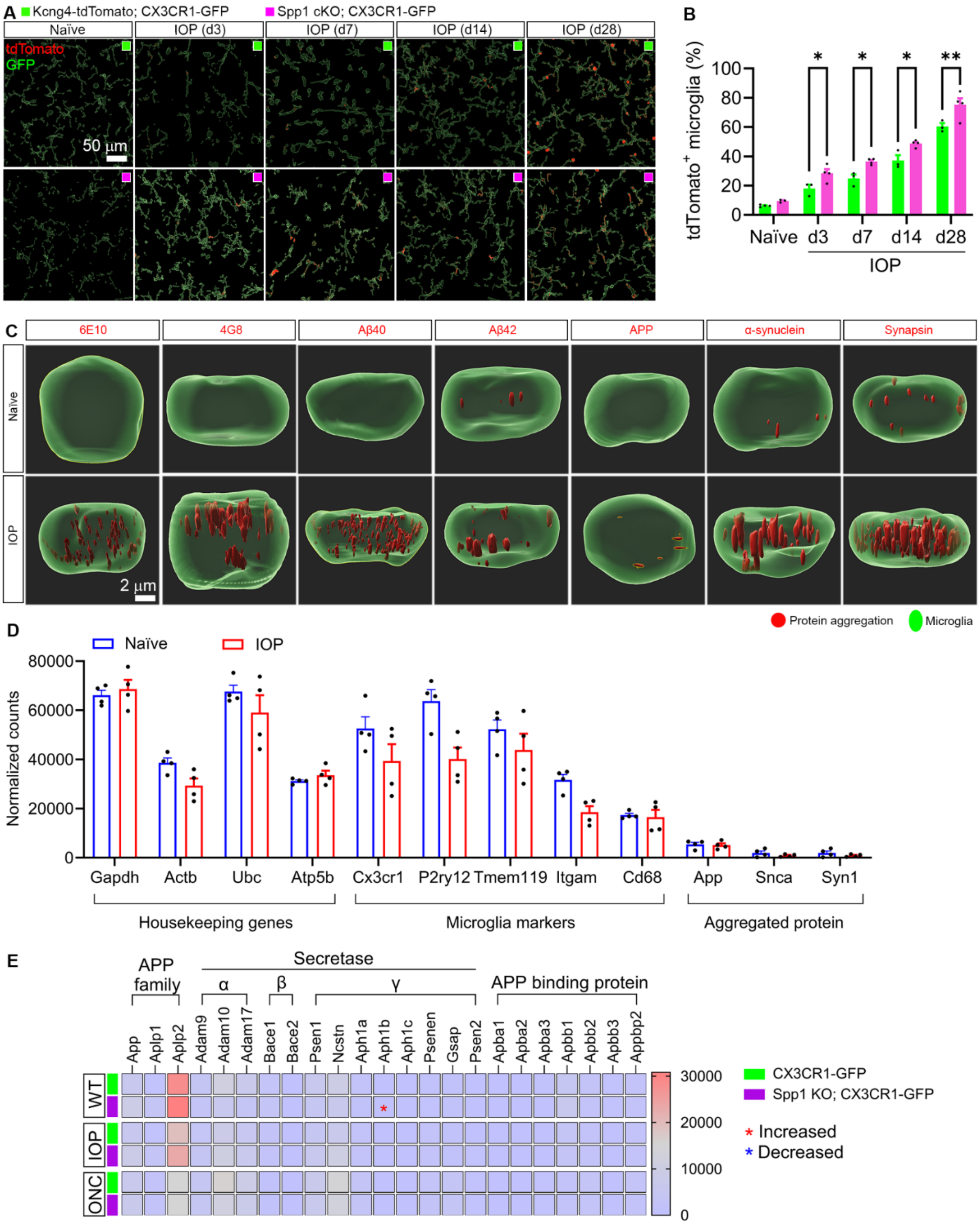

Fig. S9. Spp1 deletion causes microglial debris accumulation in glaucoma.

(A) 3D-reconstructed GFP^+ microglia from Kcng4-cre; Ai14; CX3CR1-GFP and Spp1 cKO; CX3CR1-GFP retinas under naïve conditions and on days 3, 7, 14, and 28 after IOP elevation. $tdTomato^+$ debris within microglia represents apoptotic RGCs. (**B**) Analysis of $tdTomato^+$ microglia density (n = 3–4 mice). (**C**) At 4 weeks after IOP elevation, GFP^+ microglia were FACS-sorted from CX3CR1-GFP retinas and stained with antibodies that identify specific forms, including 6E10 (Aβ40 and Aβ42), 4G8 (Aβ40 and Aβ42), Aβ40, Aβ42, APP (amyloid precursor protein), α-synuclein, and synapsin, followed by 3D reconstruction. (**D**) RNA-seq analysis of housekeeping genes (Gapdh, Actb, Ubc, and Atp5b), microglial markers (Cx3cr1, P2ry12, Tmem119, Itgam, and Cd68), APP, α-synuclein (Snca), and synapsin (Syn1) in retinal microglia from CX3CR1-GFP mice at 4 weeks after IOP elevation. (**E**) Heat map of genes related to Aβ production and processing, including APP and secretase pathways, in WT and Spp1 KO microglia at 4 weeks after IOP elevation and on day 7 after ONC. The *P* values were calculated by two-way ANOVA (B); $*p < 0.05$, $**p < 0.01$. Data are mean ± SEM.

Fig. S10. SPP1 modulates the microglial cytokine proteomic profile.

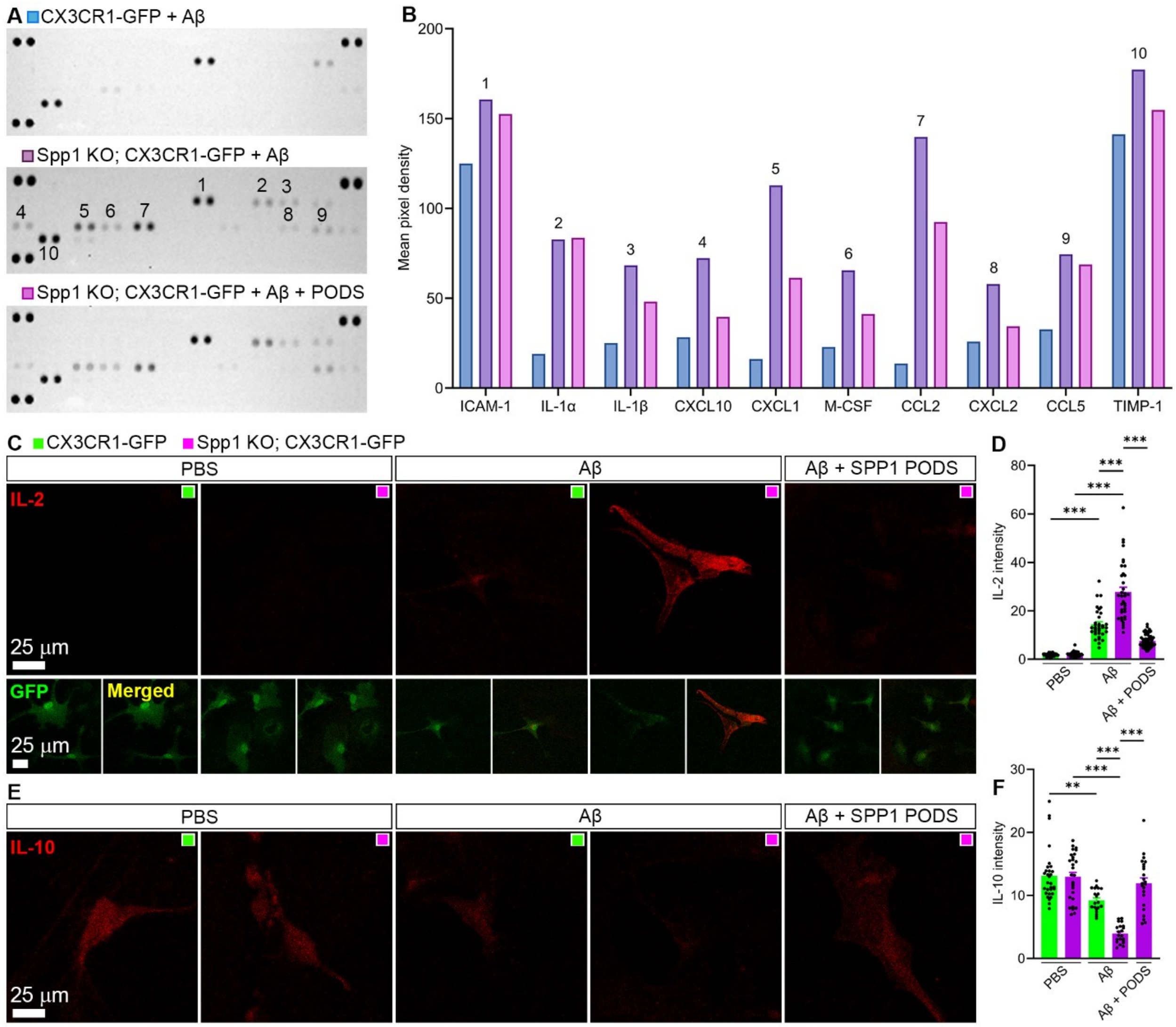

Fig. S10. SPP1 modulates the microglial cytokine proteomic profile.

(**A**) Representative membrane images from the proteome profiler mouse cytokine array showing cytokine expression in WT microglia treated with Aβ (0.5 μM), Spp1 KO microglia treated with Aβ (0.5 μM), and Spp1 KO microglia treated with Aβ (0.5 μM) plus SPP1 PODS (50 ng/ml) for 24 h. (**B**) Quantification of selected cytokines from the array in A. Numbered proteins indicated differential changes in pro-inflammatory mediators in A. (**C** and **D**) Immunostaining (C) and quantification (C) of IL-2 expression in cultured WT microglia and Spp1 KO microglia treated with PBS, Aβ (0.5 μM), or Aβ (0.5 μM) plus SPP1 PODS (50 ng/ml) for 24 h (n = 34–73 cells). (**E** and **F**) Immunostaining (E) and quantification (F) of IL-10 expression in cultured microglia (n = 20–30 cells). The *P* values were calculated by one-way ANOVA (D and F); $^{**}p < 0.01$, $^{***}p < 0.001$. Data are mean ± SEM.

Fig. S11. Pathway screening identifies macroautophagy dysfunction in Spp1-deficient microglia.

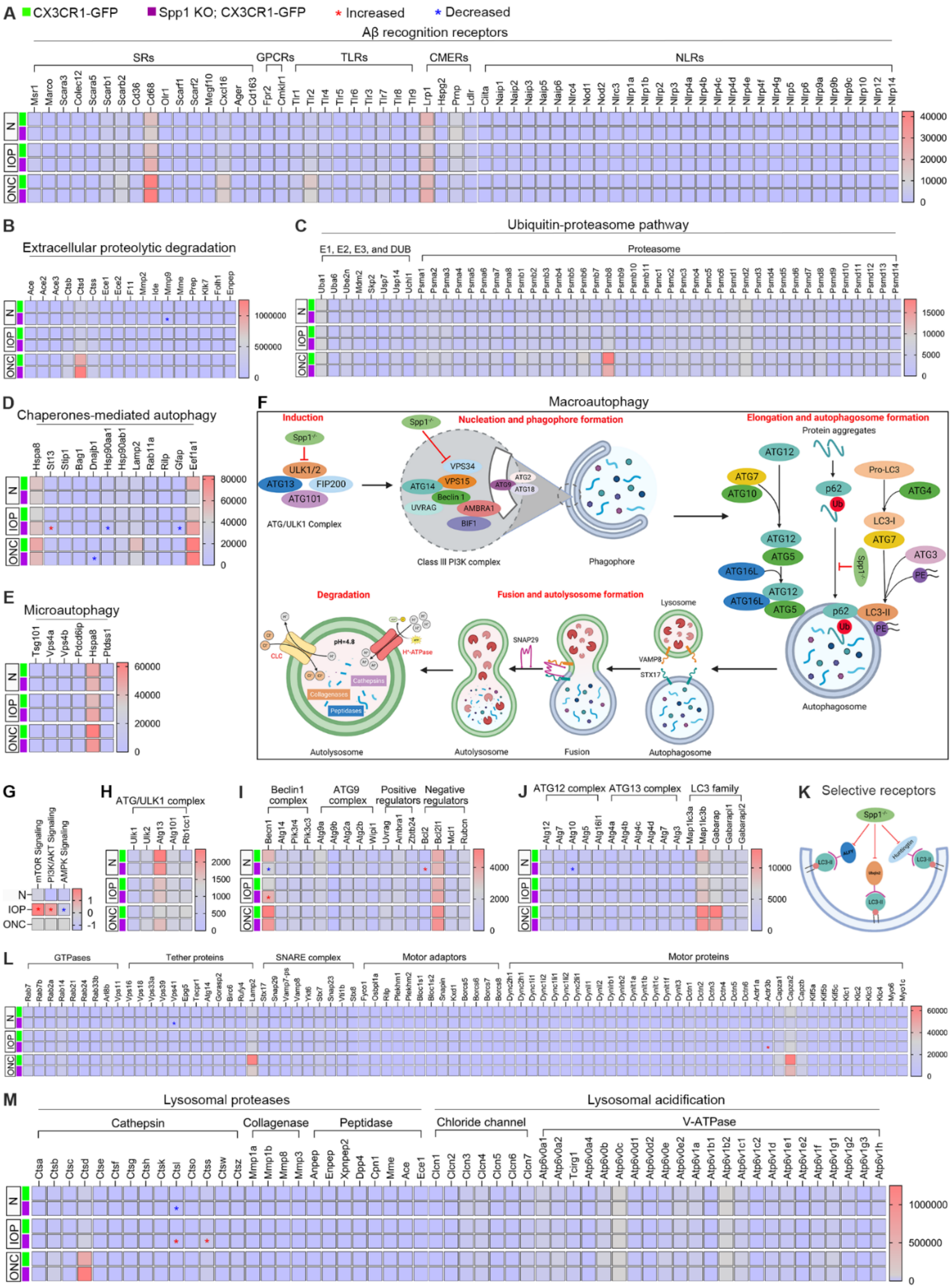

Fig. S11. Pathway screening identifies macroautophagy dysfunction in Spp1-deficient microglia.

(**A**) RNA-seq analysis of genes encoding Aβ recognition receptors, including scavenger receptors (SRs), G protein-coupled receptors (GPCRs), Toll-like receptors (TLRs), Clathrin-mediated endocytosis receptors (CMERs), and Nod-like receptors (NLRs), in WT and Spp1 KO microglia sorted from CX3CR1-GFP and Spp1 KO; CX3CR1-GFP retinas at 4 weeks after IOP elevation and on day 7 after ONC. **(B–E)** Heat maps of genes related to extracellular proteolytic degradation (B), ubiquitin–proteasome pathways (C), chaperone-mediated autophagy (D), and microautophagy (E) in microglia. (**F**) Schematic illustrating SPP1 deficiency disrupting macroautophagy in retinal microglia in glaucoma. (**G** and **H**) Heat map of signaling pathways (G) and ATG/ULK1 complex genes (H) for autophagy induction. (**I**) Heat map of genes for nucleation and phagophore formation, including Beclin1 complex, ATG9 complex, positive regulators, and negative regulators. (**J**) Heat map of elongation and autophagosome-formation genes, including ATG12, ATG13, and LC3 families. (**K**) Schematic of SPP1 deficiency effects on autophagy-selective receptors. (**L**) Heat map of genes for fusion and autolysosome formation (GTPases, Tether proteins, SNARE complex, motor adaptors, and motor proteins. (**M**) Heat map of degradation-stage genes (cathepsin, collagenases, peptidases, chloride channels, and V-ATPases). Diagram (F) was created in BioRender. Li, S. (2026) https://BioRender.com/pywxwqx. Diagram (K) was created in BioRender. Li, S. (2026) https://BioRender.com/oixjajp.

Fig. S12. Autophagy-selective receptors are downregulated in microglia.

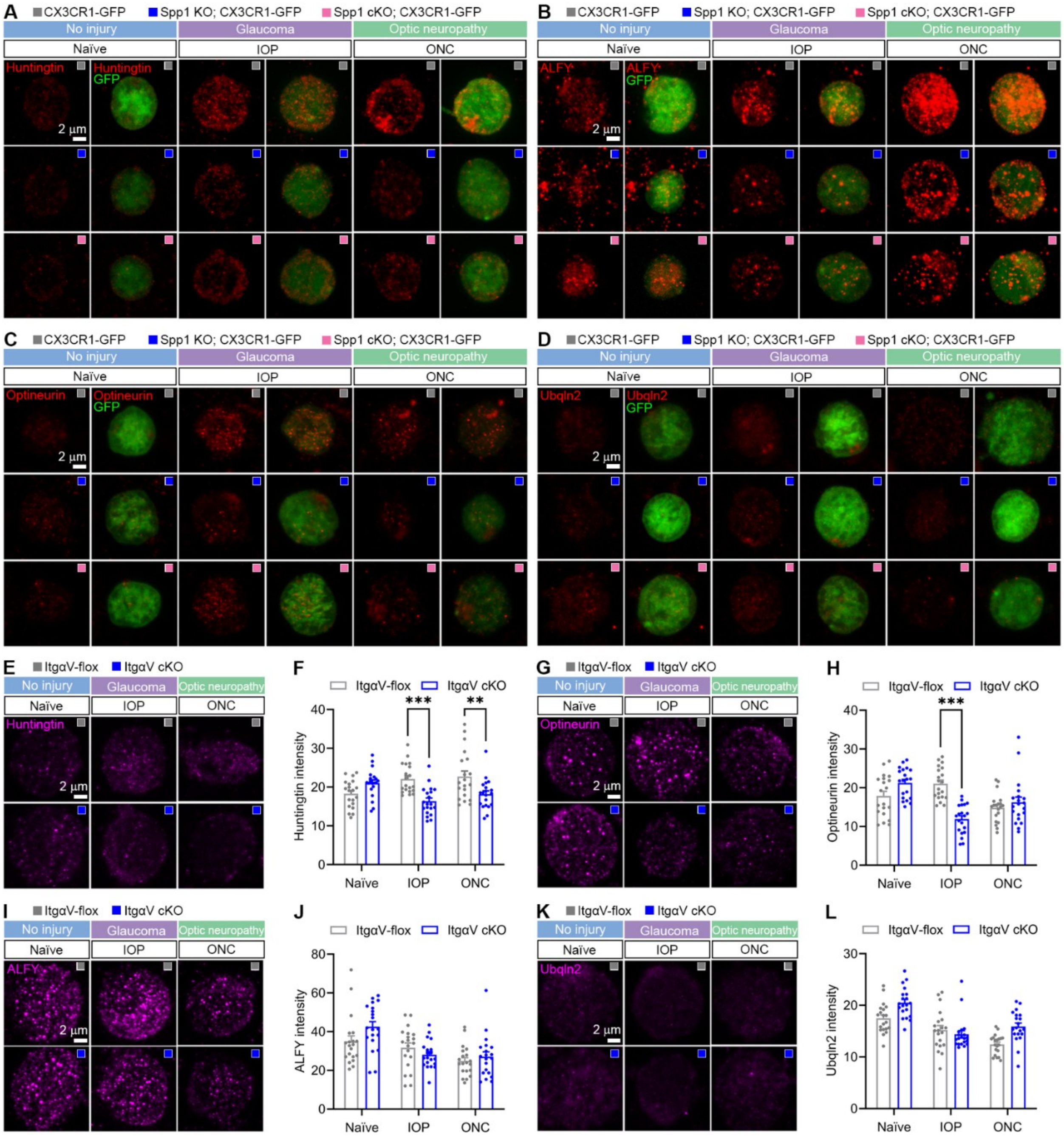

Fig. S12. Autophagy-selective receptors are downregulated in microglia.

(**A–D**) Representative images of Huntingtin (A), ALFY (B), Optineurin (C), and Ubqln2 (D) immunostaining in FACS-sorted single GFP^+ microglia from retinas of CX3CR1-GFP, Spp1 KO; CX3CR1-GFP, and Spp1 cKO; CX3CR1-GFP mice under naïve conditions, at 4 weeks after IOP elevation, and on day 7 after ONC. (**E–L**) Huntingtin immunostaining (E) and protein levels (F), Optineurin immunostaining (G) and protein levels (H), ALFY immunostaining (I) and protein levels (J), and Ubqln2 immunostaining (K) and protein levels (L) in FACS-sorted single $CD11b^+$ $CD45^{low}$ microglia from ItgαV-flox and Itgα cKO retinas under naïve conditions, at 4 weeks after IOP elevation, and on day 7 after ONC (n = 20 cells). The *P* values were calculated by two-way ANOVA (F and H); $^{**}p < 0.01$, $^{***}p < 0.001$. Data are mean ± SEM.

Fig. S13. Inhibition of autophagy-selective receptors impairs debris clearance in microglia.

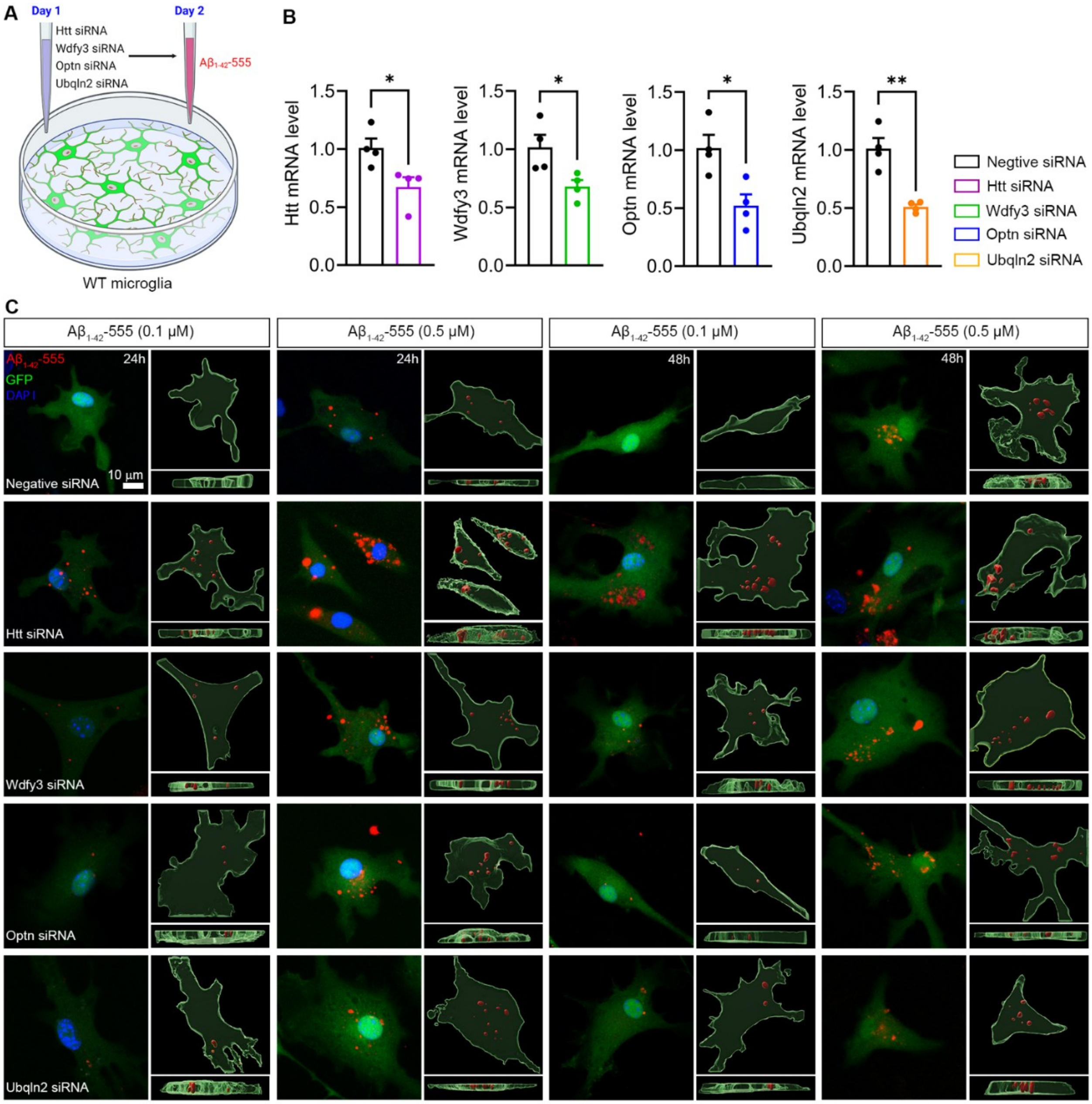

Fig. S13. Inhibition of autophagy-selective receptors impairs debris clearance in microglia.

(**A**) Experimental scheme showing cultured GFP^+ microglia from CX3CR1-GFP retinas transfected with Htt, Wdfy3, Optn, or Ubqln2 siRNA for 24h, followed by treatment with 0.1 μM or 0.5 μM Aβ protein ($A\beta_{1-42}$-555) for 24h and 48h. (**B**) qPCR validation of Htt, Wdfy3, Optn, and Ubqln2 knockdown in siRNA-treated microglia (n = 4). (**C**) Representative 3D-reconstructed images of GFP^+ microglia from CX3CR1-GFP retinas treated with negative, Htt, Wdfy3, Optn, or Ubqln2 siRNA for 24h, followed by treatment with 0.1 μM or 0.5 μM $A\beta_{1-42}$-555 for 24h and 48h. Microglial intracellular Aβ aggregates were visualized using Imaris. The *P* values were calculated by unpaired two-tailed t-test (B); *$p < 0.05$, **$p < 0.01$. Data are mean ± SEM. Diagram (A) was created in BioRender. Li, S. (2026) https://BioRender.com/ihrlxze.

Fig. S14. SPP1 overexpression reduces microglial activation and enhances debris degradation.

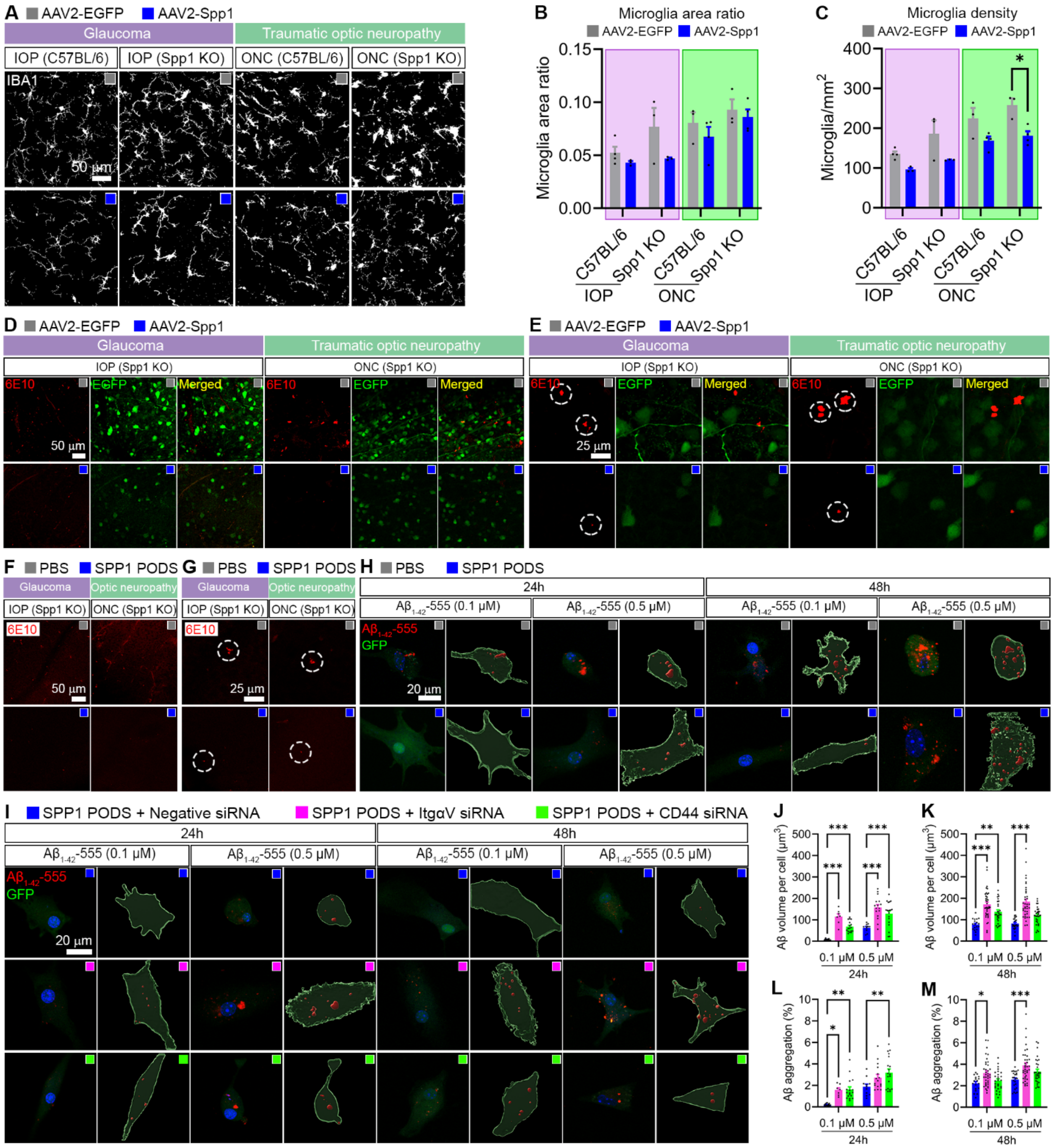

Fig. S14. SPP1 overexpression reduces microglial activation and enhances debris degradation.

(**A**) IBA1 immunostaining of retinal microglia in WT and Spp1 KO mice treated with AAV2-Spp1 or AAV2-EGFP following IOP elevation and ONC. (**B** and **C**) Quantification of microglia area ratio (B) and density (C) in WT and Spp1 KO mice with SPP1 overexpression (n = 3–4 mice). (**D** and **E**) Representative images of 6E10-labeled Aβ plaque number (**D**) and size (**E**) in retinal microglia in WT and Spp1 KO mice treated with AAV2-Spp1 or AAV2-EGFP following IOP elevation and ONC. (**F** and **G**) 6E10-labeled Aβ plaque density (F) and size (G) in retinal microglia in Spp1 KO mice treated with SPP1 PODS or PBS following IOP elevation and ONC. (**H**) Aβ accumulation in cultured Spp1 KO GFP^+ microglia treated with SPP1 PODS (50 ng/ml) and $A\beta_{1-42}$-555 (0.1 μM, 0.5 μM) for 24h and 48h. (**I**) Aβ accumulation in cultured Spp1 KO GFP^+ microglia treated with SPP1 PODS (50 ng/ml) plus control, ItgαV, or CD44 siRNA (50 nM), followed by treatment with $A\beta_{1-42}$-555 (0.1 μM, 0.5 μM) for 24h and 48h. (**J** and **K**) Quantification of intracellular Aβ volume in cultured Spp1 KO GFP^+ microglia treated with SPP1 PODS plus siRNA, followed by $A\beta_{1-42}$-555 treatment for 24h (J) and 48h (K) (n = 7–19 cells for 24h; 18–37 cells for 48h). (**L** and **M**) Quantification of Aβ aggregation in cultured Spp1 KO GFP^+ microglia treated with SPP1 PODS plus siRNA, followed by $A\beta_{1-42}$-555 treatment for 24h (L) and 48h (M) (n = 7–19 cells for 24h; 18–37 cells for 48h). The *P* values were calculated by two-way ANOVA (B, C, and J–M); $*p < 0.05$, $**p < 0.01$, $***p < 0.001$. Data are mean ± SEM.

Fig. S15. SPP1 signaling and microglial activation in human and nonhuman primate glaucomatous retinas.

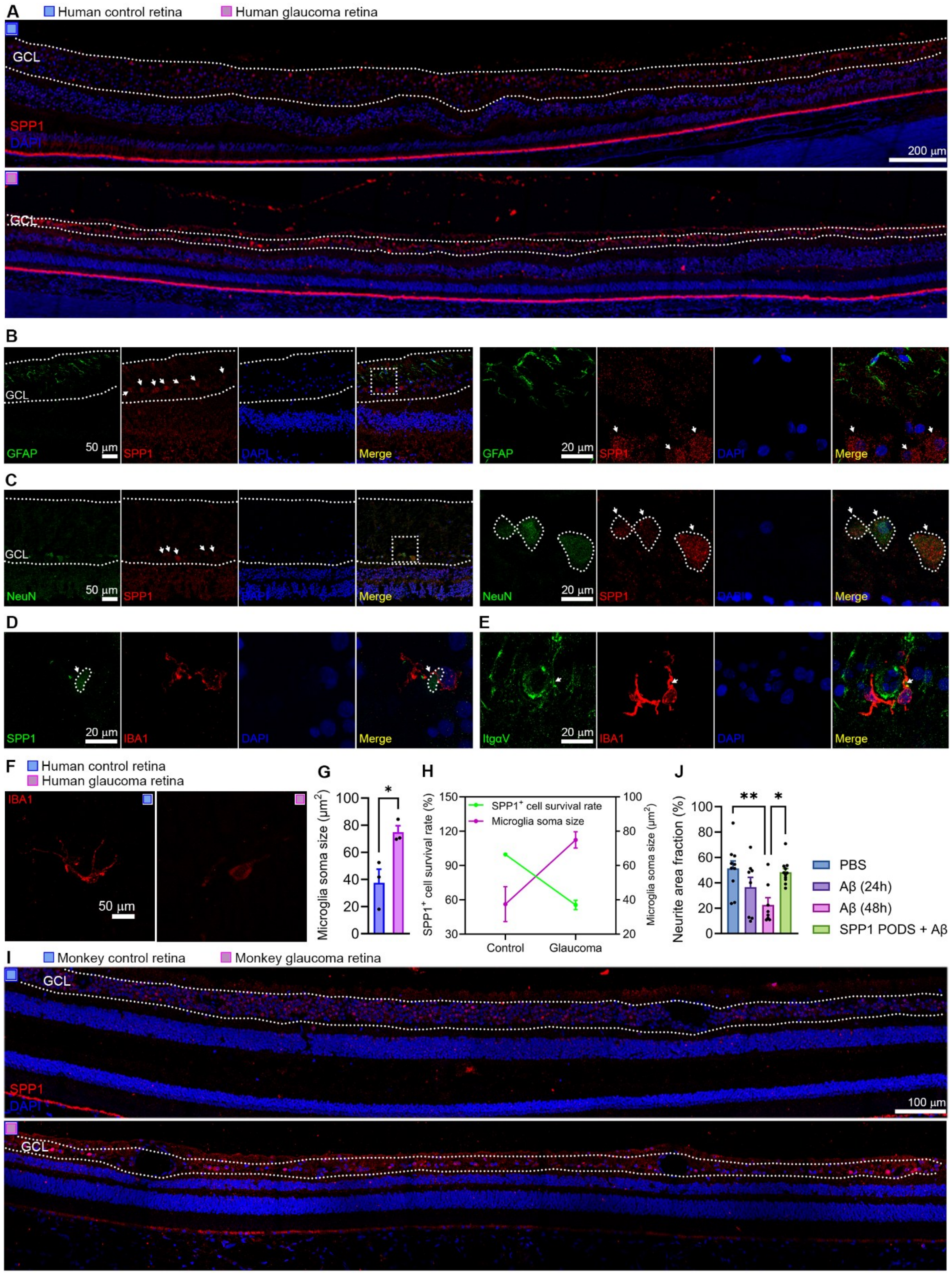

Fig. S15. SPP1 signaling and microglial activation in human and nonhuman primate glaucomatous retinas.

(**A**) SPP1 immunostaining in retinas from control donors and individuals with glaucoma. (**B–E**) Co-localization of SPP1 with $GFAP^+$ astrocytes (B), $NeuN^+$ neurons (C), $IBA1^+$ microglia (D), and the co-localization of ItgαV with $IBA1^+$ microglia (E) in control human retinas. (**F**) IBA1-labeled microglia in retinas from control donors and glaucoma patients. (**G**) Quantification of microglial soma size in control and glaucomatous retinas (n = 3 control and 3 glaucoma donors). (**H**) Correlation of $SPP1^+$ cell survival rate and microglial soma size in control and glaucomatous retinas (n = 3 control and 3 glaucoma donors). (**I**) SPP1 immunostaining in control and glaucomatous non-human primate retinas. (**J**) Quantification of neurite density in human iPSC-derived neurons co-cultured with treated microglia. Human iPSC-derived neurons were co-cultured with human microglia that had been pre-treated with control (PBS) conditions or Aβ for 24 hours or 48 hours, in the presence or absence of SPP1 PODS co-treatment. Neurite network density was evaluated by measuring the βIII-tubulin area fraction (%) (the percentage of the field of view occupied by neurites), demonstrating that SPP1 PODS protect against neurite degeneration induced by Aβ-treated microglia (n = 8–11 fields of view per condition). The *P* values were determined by unpaired two-tailed t-test (G) and one-way ANOVA (J); $*p < 0.05$, $**p < 0.01$. Data are mean ± SEM.

Table S1. Key resource.

**Antibodies**	**Source**	**Identifier**
Mouse anti-BRN3A	Millipore	Cat# MAB1585; RRID: AB_94166
Mouse anti-Neurofilament H (SMI32)	BioLegend	Cat# 801701; RRID: AB_2564642
Goat anti-SPP1	R&D Systems	Cat# AF808; RRID: AB_2194992
Goat anti-human SPP1	R&D Systems	Cat# AF1433; RRID: AB_354791
Rabbit anti-SPP1	MyBioSource	Cat# MBS4506382
Mouse anti-Glutamine Synthetase (GS)	Abcam	Cat# ab64613; RRID: AB_1140869
Rabbit anti-IBA1	Fujifilm Wako	Cat# 019-19741; RRID: AB_839504
Goat anti-IBA1	Abcam	Cat# AB5076; RRID: AB_2224402
Rabbit anti-IBA1	Proteintech	Cat# 10904-1-AP; RRID: AB_2224377
Mouse anti-CD44	Novus Biologicals	Cat# NBP1-47386; RRID: AB_10010339
Rat anti-ItgαV	Thermo Fisher Scientific	Cat# 14-0512-82; RRID: AB_467296
Chicken anti-GFAP	Abcam	Cat# ab4674; RRID: AB_304558
Rabbit anti-Clec7a	Novus Biologicals	Cat# NBP2-41170; RRID: AB_3301714
Rabbit anti-TMEM119	Abcam	Cat# ab209064; RRID: AB_2800343
Rabbit anti-P2RY12	Novus Biologicals	Cat# NBP1-78249; RRID: AB_11015520
Rabbit anti-NeuN	Cell Signaling Technology	Cat# 24307; RRID: AB_2651140
Rat anti-C3	GeneTex	Cat# GTX11862; RRID: AB_367355
Rabbit anti-LC3B	Novus Biologicals	Cat# NB100-2220SS; RRID: AB_791015

Rabbit anti-P62	Millipore	Cat# P0067; RRID: AB_1841064
Rat anti-CD11b (PE-Cyanine7)	eBioscience	Cat# 25-0112-82; RRID: AB_469588
Rat Anti-Mouse CD45 (Alexa Fluor™ 488)	BD Biosciences	Cat# 567377; RRID: AB_2916575
Rabbit anti-β3-tubulin	Sigma-Aldrich	Cat# T2200; RRID: AB_262133
Rabbit anti-MAP2	ABclonal	Cat# A22205; RRID: AB_3698579
Mouse anti-β-Amyloid, 6E10	BioLegend	Cat# 803004; RRID: AB_2715854
Mouse anti-β-Amyloid, 6E10 (Alexa Fluor® 488)	BioLegend	Cat# 803013; RRID: AB_2564765
Mouse anti-β-Amyloid, 6E10 (Alexa Fluor® 594)	BioLegend	Cat# 803019; RRID: AB_2734552
Mouse anti-β-Amyloid, 6E10 (Alexa Fluor® 647)	BioLegend	Cat# 803020; RRID: AB_2783373
Mouse anti-β-Amyloid, 4G8 (Alexa Fluor® 594)	BioLegend	Cat# 800715; RRID: AB_2721291
Mouse anti-β-Amyloid, 1-40	BioLegend	Cat# 805409; RRID: AB_2734555
Mouse anti-β-Amyloid, 1-42	BioLegend	Cat# 805509; RRID: AB_2783381
Mouse anti-APP, C-Terminal Fragment	BioLegend	Cat# 802803; RRID: AB_2715853
Mouse anti-α-synuclein	R&D Systems	Cat# MAB13381SP; RRID: AB_3657625
Rabbit anti-Synapsin	Chemicon	Cat# MAB355
Rabbit anti-Huntingtin	Cell Signaling Technology	Cat# 5656; RRID: AB_10827977
Rabbit anti-Wdfy3	Proteintech	Cat# 55009-1-AP; RRID: AB_10863126
Rabbit anti-Optineurin	Proteintech	Cat# 10837-1-AP; RRID: AB_2156665
Mouse anti-Ubqln2	Novus Biologicals	Cat# NBP2-25164; RRID: AB_2885154
Rabbit anti-IL-2	Proteintech	Cat# 26156-1-AP; RRID: AB_2880407

Mouse anti-IL-10	Abcam	Cat# ab25073; RRID: AB_448588
**Bacterial and virus strains**		
AAV2-Spp1-EGFP	This study	N/A
AAV2-EGFP	This study	N/A
**Biological samples**		
Mouse eye tissue	Mouse strain is listed in the "Experimental Models: Organisms/Strains"	N/A
Human eye tissue	Lions Gift of Sight	N/A
Human brain tissue	Massachusetts Alzheimer's Disease Research Center	N/A
Nonhuman primate eye tissue	Devers Eye Institute	N/A
Human iPSC-derived microglia	Elixirgen Scientific	MG-SeV-CW50065
Human iPSC-derived neurons	Elixirgen Scientific	EX-SeV-CW50065
WT microglia	This study	N/A
Spp1 KO	This study	N/A
C57BL/6 RGCs	This study	N/A
**Chemicals, peptides, and recombinant proteins**		
TGF-β1	R&D Systems	Cat# 7666-MB/CF
SB431542 (TGF-b 1 receptor inhibitor)	Tocris Bioscience	Cat# 1614
RO5-3335 (Runx1 inhibitor)	Tocris Bioscience	Cat# 4694
HLM006474 (E2F1 inhibitor)	Tocris Bioscience	Cat# 5283
Silencer™ Negative Control #1 siRNA	Thermo Fisher Scientific	Cat# AM4611
CD44 siRNA	Thermo Fisher Scientific	Cat# 165814

ItgαV siRNA	Thermo Fisher Scientific	Cat# AM16708
Htt siRNA	Thermo Fisher Scientific	Cat# 164755
Wdfy3 siRNA	Thermo Fisher Scientific	Cat# 178783
Optn siRNA	Thermo Fisher Scientific	Cat# 179088
Ubqln2 siRNA	Thermo Fisher Scientific	Cat# 73439
SPP1 PODS	Cell Guidance Systems	Cat# PPH319-250
pHrodo™Red E. coli BioParticles™ Conjugates	Thermo Fisher Scientific	Cat# P35361
Beta-Amyloid (1-42), HiLyte™ Fluor 555-labeled	AnaSpec	Cat# AS-60480-01
DMEM/F-12	Gibco	Cat# 12500062
DMEM/F-12 (no phenol red)	Gibco	Cat# 11039021
Amino acid-free medium	Fujifilm	Cat# 048-33575
Human fibronectin	Corning	Cat# 354008
Poly-L-Ornithine	Sigma-Aldrich	Cat# P4957
Geltrex Basement Membrane Matrix	Thermo Fisher Scientific	Cat# A4000046801
Neurobasal Medium	Thermo Fisher Scientific	Cat# 21103049
GlutaMAX	Thermo Fisher Scientific	Cat# 35050061
Penicillin-Streptomycin	Thermo Fisher Scientific	Cat# 15140122
ROCK inhibitor Y27632	MedChemExpress	Cat# HY-10583
**Critical commercial assays**		
Dynabeads™ Mouse Pan T (Thy1.2)	Invitrogen	Cat# 11443D
MiniMACS™ Starting Kit	Miltenyi Biotec	Cat# 130-090-312
RNeasy Plus Micro Kit	Qiagen	Cat# 74034
High-Capacity cDNA Reverse Transcription Kit	Applied Biosystems	Cat# 4368814

SYBR Green PCR Master Mix	Applied Biosystems	Cat# 4309155
FastStart Essential DNA Green Master	Roche Diagnostics	Cat# 06402712001
REDExtract-N-Amp™ Tissue PCR Kit	Sigma-Aldrich	Cat# XNAT-100RXN
DreamTaq PCR Master Mixes	Thermo Fisher Scientific	Cat# K1081
Agilent RNA 6000 Pico Kit	Agilent Technologies	Cat# 5067-1513
Lipofectamine™ 3000 Transfection Kit	Invitrogen	Cat# L3000001
ScienCell Microglia Medium Kit	ScienCell Research Laboratories	Cat# 1901
Autophagic Flux Assay Kit	Dojindo Molecular Technologies	Cat# A562
Proteome Profiler Mouse Cytokine Array Kit	R&D Systems	Cat# ARY006
**Experimental Models: Organisms/Strains**		
Mouse: C57BL/6J (WT)	Jackson Laboratory	Cat# 000664
Mouse: B6.129S6(Cg)-Spp1^{tm1Blh}/J	Jackson Laboratory	Cat# 004936
Mouse: B6.Spp1$^{fl\text{-}EGFP\text{-}stop\text{-}tdTomato}$	Produced by Cyagen, deposited at Jackson Laboratory	Cat# 038709
Mouse: B6.Cg-Gt(ROSA)26Sor$^{tm14(CAGtdTomato)Hze}$/J	Jackson Laboratory	Cat# 007914
Mouse: C57BL/6J-*Atg7*^{em1Lutzy}/J	Jackson Laboratory	Cat# 034429
Mouse: B6J.B6N(Cg)-*Cx3cr1*$^{tm1.1(cre)Jung}$/J	Jackson Laboratory	Cat# 025524
Mouse: B6.129P2(Cg)-*Cx3cr1*^{tm1Litt}/J	Jackson Laboratory	Cat# 005582
Mouse: B6.ItgαV$^{flox/flox}$	a gift from Adam Lacy-Hulbert, Benaroya Research Institute	N/A
Mouse: B6.129(SJL)-*Kcng4*$^{tm1.1(cre)Jrs}$/J	Jackson Laboratory	Cat# 029414

Mouse: B6.Cg-Tg(Gfap-cre)77.6Mvs/2J	Jackson Laboratory	Cat# 024098
Mouse: Tg(Thy1-cre/ERT2,-EYFP)HGfng/PyngJ	Jackson Laboratory	Cat# 012708
**Deposited data**		
RNA-seq raw data	This study	NCBI GEO: GSE304931
**Software and algorithms**		
GraphPad Prism 10	https://www.graphpad.com/	RRID: SCR_002798
Fiji (ImageJ)	https://fiji.sc/	RRID: SCR_002285
Adobe Photoshop 2025	https://www.adobe.com/products/photoshop.html	RRID: SCR_014199
RStudio	https://www.rstudio.com/	RRID: SCR_000432
Matlab	https://www.mathworks.com/products/matlab	RRID: SCR_001622
Python 3.10	https://python.org	RRID: SCR_008394
Imaris	https://imaris.oxinst.com/	RRID: SCR_007370
LAS X	https://www.leica-microsystems.com/	RRID: SCR_013673
FlowJo	https://www.flowjo.com/	RRID: SCR_008520
Biorender	https://www.biorender.com/	RRID: SCR_018361

Table S2. List of oligonucleotides.

**Primers used for mouse genotyping**		
**Name**	**Forward (5'-3')**	**Reverse (5'-3')**
Mouse B6.129S6(Cg)-Spp1^{tm1Blh}/J	GTCTGGAGAACATGGGTGCT	GGGTGCAGGCTGTAAAGCTA
Mouse B6.Spp1$^{fl-EGFP-stop-tdTomato}$	AGTGTAGAGCGTCATTGCAGTTT	TCGGCTACATCCTAAATCCCATT
Mouse B6.Cg-Tg(Gfap-cre)77.6Mvs/2J	TCCATAAAGGCCCTGACATC	TGCGAACCTCATCACTCGT
Mouse B6.Cg-Gt(ROSA)26Sor$^{tm14(CAG-tdTomato)Hze}$/J (Wild type)	AAGGGAGCTGCAGTGGAGTA	CCGAAAATCTGTGGGAAGTC
Mouse B6.Cg-Gt(ROSA)26Sor$^{tm14(CAG-tdTomato)Hze}$/J (Mutant)	CTGTTCCTGTACGGCATGG	GGCATTAAAGCAGCGTATCC
Mouse C57BL/6J-Atg7^{em1Lutzy}/J	CTTCTGCAAGGCCCACTAAC	CCCGGAGAAGTCTGAGTCTG
Mouse B6.ItgαV$^{flox/flox}$	GGTGACTCAATCTGTGACCTTCAGC	CACAAATCAAGGATGACCAAACTGAG
Mouse B6.129(SJL)-Kcng4$^{tm1.1(cre)Jrs}$/J (Wild type)	GATCCCTGGCCTGACAGTCT	TGGCCACCCACATTCACT
Mouse B6.129(SJL)-Kcng4$^{tm1.1(cre)Jrs}$/J (Mutant)	CAGATTTTTCCTCCTCTCCTGA	GTGTATCTGGGCTGCCTTGT
Mouse Tg(Thy1-cre/ERT2,-EYFP)HGfng/PyngJ (Transgene)	TCTGAGTGGCAAAGGACCTTAGG	CGCTGAACTTGTGGCCGTTTACG
Mouse Tg(Thy1-cre/ERT2,-EYFP)HGfng/PyngJ (Positive control)	CAAATGTTGCTTGTCTGGTG	GTCAGTCGAGTGCACAGTTT
Mouse B6J.B6N(Cg)-Cx3cr1$^{tm1.1(cre)Jung}$/J (Wild type)	CCTCAGTGTGACGGAGACAG	
Mouse B6J.B6N(Cg)-Cx3cr1$^{tm1.1(cre)Jung}$/J (Common)	GCAGGGAAATCTGATGCAAG	
Mouse B6J.B6N(Cg)-Cx3cr1$^{tm1.1(cre)Jung}$/J (Mutant)	GACATTTGCCTTGCTGGAC	

Mouse B6.129P2(Cg)-Cx3cr1^{tm1Litt}/J (Wild type)	GTCTTCACGTTCGGTCTGGT	
Mouse B6.129P2(Cg)-Cx3cr1^{tm1Litt}/J (Common)	CCCAGACACTCGTTGTCCTT	
Mouse B6.129P2(Cg)-Cx3cr1^{tm1Litt}/J (Mutant)	CTCCCCCTGAACCTGAAA C	
**Primers used for qRT-PCR**		
**Name**	**Sequence (5'-3')**	
	**Forward**	**Reverse**
Spp1	AGCAAGAAACTCTTCCAAGCAA	GTGAGATTCGTCAGATTCATCCG
Cd44	CCACAGCCTCCTTTCAATAACC	GGAGTCTTCGCTTGGGGTA
ItgαV	CCGTGGACTTCTTCGAGCC	CTGTTGAATCAAACTCAATGGGC
IL-18	GACTCTTGCGTCAACTTCAAGG	CAGGCTGTCTTTTGTCAACGA
NOS	TGACGGCAAACATGACTTCAG	GCGTACCGGATGAGCTGTG
A2M	GTGACCGCCTTGGAGTCAAT	TATGCCGCTCGTTCATACAG
IL-6	TTCCATCCAGTTGCCTTCTTG	GAAGGCCGTGGTTGTCACC
Ccl5	GCTGCTTTGCCTACCTCTCC	TCGAGTGACAAACACGACTGC
VEGF	GAAAATCACTGTGAGCCTTGTTC	TGCAAGTACGTTCGTTTAACTCA
IL-13	CTTGCTTGCCTTGGTGGTCT	CACAGGGGAGTCTGGTCTTG
Arg1	CTCCAAGCCAAAGTCCTTAGAG	AGGAGCTGTCATTAGGGACATC
IL-2	GTCAACAGCGCACCCACTT	TGCTTCCGCTGTAGAGCTTG
IL-10	CAGTACAGCCGGGAAGACAA	CCTGGGGCATCACTTCTACC
TNF-α	ATGCTGGGACAGTGACCTGG	CCTTGATGGTGGTGCATGAG
IL-1β	TGCCACCTTTTGACAGTGATG	TGATGTGCTGCTGCGAGATT
Sqstm1	AGGATGGGGACTTGGTTGC	TCACAGATCACATTGGGGTGC
Optn	ATGTCCCATCAACCTCTGAGC	TCAAATCGCCCTTTCATAGCTTG
Ubqln2	GCCGAGCCCAAAATCATCAAA	ATCTTTCCGGCGAAAATCAGC
Nbr1	GGAAATCAGCTACAGATGCAAGT	ATCCCAAGACTCTCACCAGTG
Wdfy3	GAGGCTCTGGAGTGTGATTACG	GTGGCCGTCTCCTTCAGTG
Calcoco2	GCCCCATACCTACCTTGCTG	TCGAGGGATGAACTTTTCAGTG
Htt	CTCCATGCTGTTACGACTCATC	AGGGAGGAAGGAGCCAAAATC
C3	CCAGCTCCCCATTAGCTCTG	GCACTTGCCTCTTTAGGAAGTC
Gapdh	GGTTGTCTCCTGCGACTTCAA	CCTGTTGCTGTAGCCGTATTCAT

Supplementary movies

Movie S1. Live-cell time-lapse imaging of Aβ degradation in wild-type microglia treated with PBS. The video shows Aβ degradation latency in cultured wild-type microglia treated with PBS. Live-cell imaging was taken for 24 hours.

Movie S2. Live-cell time-lapse imaging of Aβ degradation in Spp1 KO microglia treated with PBS. The video shows Aβ degradation latency in cultured Spp1 KO microglia treated with PBS. Live-cell imaging was taken for 24 hours.

Movie S3. Live-cell time-lapse imaging of Aβ degradation in wild-type microglia treated with Aβ. The video shows Aβ degradation latency in cultured wild-type microglia treated with 0.5 μM Aβ. Live-cell imaging was taken for 24 hours.

Movie S4. Live-cell time-lapse imaging of Aβ degradation in Spp1 KO microglia treated with Aβ. The video shows Aβ degradation latency in cultured Spp1 KO microglia treated with 0.5 μM Aβ. Live-cell imaging was taken for 24 hours.

Movie S5. Live-cell time-lapse imaging of Aβ degradation in wild-type microglia treated with Aβ plus SPP1 PODS. The video shows Aβ degradation latency in cultured wild-type microglia treated with Aβ (0.5 μM) and SPP1 PODS (50 ng/ml). Live-cell imaging was taken for 24 hours.

Movie S6. Live-cell time-lapse imaging of Aβ degradation in Spp1 KO microglia treated with Aβ plus SPP1 PODS. The video shows Aβ degradation latency in cultured Spp1 KO microglia treated with Aβ (0.5 μM) and SPP1 PODS (50 ng/ml). Live-cell imaging was taken for 24 hours.
